# Biosynthesis, herbivore induction, and defensive role of phenylacetaldoxime glucoside

**DOI:** 10.1093/plphys/kiad448

**Published:** 2023-08-16

**Authors:** Andrea T Müller, Yoko Nakamura, Michael Reichelt, Katrin Luck, Eric Cosio, Nathalie D Lackus, Jonathan Gershenzon, Axel Mithöfer, Tobias G Köllner

**Affiliations:** Research Group Plant Defense Physiology, Max Planck Institute for Chemical Ecology, D-07745 Jena, Germany; Department of Biochemistry, Max Planck Institute for Chemical Ecology, D-07745 Jena, Germany; Pontifical Catholic University of Peru, Institute for Nature Earth and Energy (INTE-PUCP), San Miguel 15088, Lima, Peru; Research Group Biosynthesis/NMR, Max Planck Institute for Chemical Ecology, D-07745 Jena, Germany; Department of Natural Product Research, Max Planck Institute for Chemical Ecology, D-07745 Jena, Germany; Department of Biochemistry, Max Planck Institute for Chemical Ecology, D-07745 Jena, Germany; Department of Biochemistry, Max Planck Institute for Chemical Ecology, D-07745 Jena, Germany; Pontifical Catholic University of Peru, Institute for Nature Earth and Energy (INTE-PUCP), San Miguel 15088, Lima, Peru; Department of Biochemistry, Max Planck Institute for Chemical Ecology, D-07745 Jena, Germany; Department of Biochemistry, Max Planck Institute for Chemical Ecology, D-07745 Jena, Germany; Research Group Plant Defense Physiology, Max Planck Institute for Chemical Ecology, D-07745 Jena, Germany; Department of Natural Product Research, Max Planck Institute for Chemical Ecology, D-07745 Jena, Germany

## Abstract

Aldoximes are well-known metabolic precursors for plant defense compounds such as cyanogenic glycosides, glucosinolates, and volatile nitriles. They are also defenses themselves produced in response to herbivory; however, it is unclear whether aldoximes can be stored over a longer term as defense compounds and how plants protect themselves against the potential autotoxic effects of aldoximes. Here, we show that the Neotropical myrmecophyte tococa (*Tococa quadrialata*, recently renamed *Miconia microphysca*) accumulates phenylacetaldoxime glucoside (PAOx-Glc) in response to leaf herbivory. Sequence comparison, transcriptomic analysis, and heterologous expression revealed that 2 cytochrome P450 enzymes, CYP79A206 and CYP79A207, and the UDP-glucosyltransferase UGT85A123 are involved in the formation of PAOx-Glc in tococa. Another P450, CYP71E76, was shown to convert PAOx to the volatile defense compound benzyl cyanide. The formation of PAOx-Glc and PAOx in leaves is a very local response to herbivory but does not appear to be regulated by jasmonic acid signaling. In contrast to PAOx, which was only detectable during herbivory, PAOx-Glc levels remained high for at least 3 d after insect feeding. This, together with the fact that gut protein extracts of 3 insect herbivore species exhibited hydrolytic activity toward PAOx-Glc, suggests that the glucoside is a stable storage form of a defense compound that may provide rapid protection against future herbivory. Moreover, the finding that herbivory or pathogen elicitor treatment also led to the accumulation of PAOx-Glc in 3 other phylogenetically distant plant species suggests that the formation and storage of aldoxime glucosides may represent a widespread plant defense response.

## Teaser

The formation and glucosylation of a highly reactive aldoxime are part of the plant defense system against herbivores and pathogens.

## Introduction

Aldoximes are widely distributed in plants and function as biosynthetic precursors for several classes of defense compounds. They have long been described as precursors of cyanogenic glycosides (Tapper et al. 1967; Andersen et al. 2000) and glucosinolates (Underhill 1967; Wittstock and Halkier 2000), 2 classes of phytoanticipins that can be activated during leaf damage to release hydrogen cyanide, isothiocyanates, thiocyanates, nitriles, and other toxic products (Dewick 1984). In addition, aldoximes act as precursors for the formation of the phytoalexin camalexin (Glawischnig 2007) and volatile nitriles (Irmisch et al. 2014). Such nitriles are often important constituents of the volatile blend of herbivore-damaged leaves and contribute to direct and indirect plant defense against insect herbivores (Clavijo McCormick et al. 2014; Irmisch et al. 2014). In addition to being rapidly metabolized to other defense compounds, some semivolatile aldoximes, such as (*E*)- and (*Z*)-phenylacetaldoxime (PAOx), can accumulate as toxins during herbivory (Irmisch et al. 2013), while other more volatile aldoximes such as 2- and 3-methylbutyraldoxime or (*E*)- and (*Z*)-isobutyraldoxime are released from herbivore-damaged plants to attract natural enemies of herbivores (Clavijo McCormick et al. 2014).

Several cytochrome P450s (CYPs) have been described to mediate the formation and metabolism of aldoximes in angiosperms and gymnosperms (Sorensen et al. 2018). P450s from the CYP79 family have been shown to catalyze the formation of aldoximes from amino acids or amino acid derivatives such as phenylalanine, tyrosine, tryptophan, valine, leucine, isoleucine, and chain-elongated forms of methionine (Sorensen et al. 2018). Mechanistically, this reaction involves 2 consecutive *N*-hydroxylations, followed by a dehydration and a decarboxylation step to release the corresponding oxime (Halkier et al. 1995; Sibbesen et al. 1995). In Brassicales, CYP83 enzymes convert aldoximes to *S*-alkylthiohydroximates, resulting eventually in glucosinolates (Naur et al. 2003; Zhu et al. 2012). Alternatively, CYP71/736 enzymes found in different plant families use aldoximes as substrates to form nitriles (Irmisch et al. 2014; Yamaguchi et al. 2016; Hansen et al. 2018) or hydroxynitriles (Bak et al. 1998; Jørgensen et al. 2011; Takos et al. 2011; Yamaguchi et al. 2014; Knoch et al. 2016). The latter are further converted to cyanogenic glycosides (α-hydroxynitriles) or, in rare cases, to noncyanogenic glycosides (β- or γ-hydroxynitrile glycosides) via a glycosylation reaction catalyzed by UDP-glycosyltransferases of the family 85 (UGT85) (Jones et al. 1999; Takos et al. 2011; Knoch et al. 2016). Nitriles, on the other hand, are either emitted as volatiles or further metabolized to their respective acids or amides by the action of nitrilases (Irmisch et al. 2014; Günther et al. 2018). Unlike glucosinolates and cyanogenic glycosides, which are preformed and activated only upon herbivory or pathogen infection, volatile aldoximes and nitriles are often formed in response to biotic stressors, and their formation is closely linked to the expression of the corresponding P450 enzymes (Irmisch et al. 2013, 2014; Luck et al. 2016).

Tococa (*Tococa quadrialata*, recently renamed *Miconia microphysca* [Michelang.]), is a Neotropical shrub species that lives in close association with symbiotic ants. As a so-called ant-plant, tococa provides ants with preformed nesting sites—hollow structures at the base of the leaf blade called domatia—and in return is defended by the ants against herbivores, pathogens, and encroaching vines (Morawetz et al. 1992; Michelangeli 2003; Dejean et al. 2006; Gonzalez-Teuber et al. 2014). While the role of the ants in this symbiotic plant–insect system has been extensively studied, not much is known about the chemical defenses of tococa. Knowledge about natural predators of tococa is also limited. Only leafcutter ants and unspecified beetle and caterpillar species are mentioned in the literature (Alvarez et al. 2001; Michelangeli 2003; Dejean et al. 2006). In a recent study (Müller et al. 2022), we investigated the response of tococa to herbivory by a generalist *Spodoptera* species under field conditions. A number of herbivore-induced metabolites were detected, including benzyl cyanide, and a previously unknown metabolite, identified in the present study as PAOx glucoside (PAOx-Glc). Here, we aimed to investigate the biosynthesis and biological role of PAOx and PAOx-Glc in tococa. We used transcriptomics and heterologous expression in *Escherichia coli*, *Saccharomyces cerevisiae*, and *Nicotiana benthamiana* to identify enzymes involved in the formation and metabolism of aldoximes. Our studies on the occurrence, inducibility, and turnover of PAOx-Glc and PAOx suggest that the formation of these compounds is a very local response to tissue damage. These findings, together with the results from glucosidase assays with insect gut protein extracts and growth inhibition assays with various pathogens, suggest that aldoximes are involved in protecting the wound site. In addition, PAOx-Glc as a stable storage form of PAOx may provide rapid protection against subsequent herbivory.

## Results

### Identification of PAOx-Glc in wounded tococa leaves

**Figure 1.**
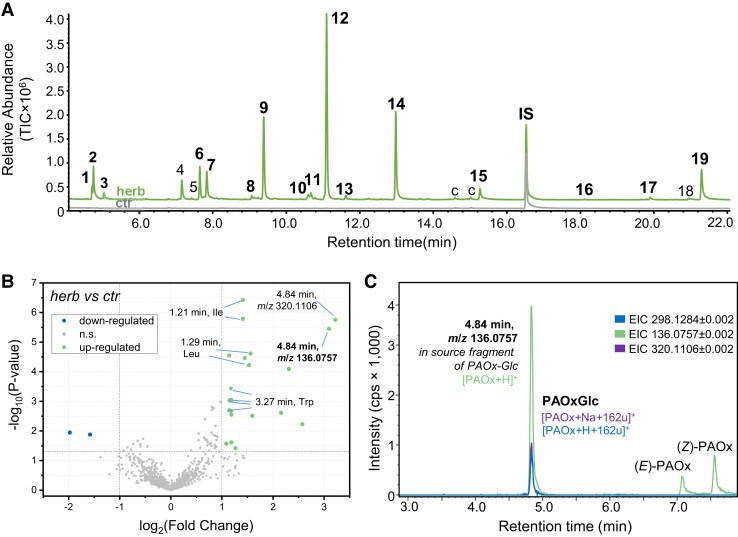
Herbivore-induced volatile and nonvolatile compounds in *T. quadrialata*. **A)** Comparison of the volatile bouquets of herbivore-damaged leaves (herb) and undamaged leaves (ctr). *S. littoralis* larvae were feeding on the leaves for 24 h, and volatiles were collected simultaneously. Compounds were analyzed using GC–MS and the total ion chromatograms (TIC) are shown. IS, internal standard; c, contamination; 1, 2-hexenal*; 2, 3-hexenol*, 3, 1-hexanol*; 4, 4-oxo-hex-2-enal and benzaldehyde; 5, unidentified; 6, 1-octen-3-ol*; 7, octan-3-one*; 8, benzyl alcohol*; 9, β-ocimene*; 10, linalool*; 11, nonanal*; 12, (*E*)-4,8-dimethyl-nonatriene (DMNT)*; 13, benzyl cyanide*; 14, methyl salicylate*; 15, indole*; 16, (*E*)-β-caryophyllene*; 17, α-farnesene*; 18, nerolidol; 19, (*E*,*E*)-4,8,12-trimethyl-1,3,7,11-tridecatetraene (TMTT)*. All compounds marked with an asterisk were identified using authentic standards, while the remaining compounds were tentatively identified using the NIST17 mass spectra library. **B)** Methanolic extracts of herbivore-treated (herb) and undamaged control (ctr) leaves were analyzed with high-resolution LC-qTOF-MS operating in positive ionization mode and the relative abundances of the features compared. The volcano plot visualizes the metabolic differences, where the bright green color marks features that accumulate upon herbivory (FC > 2, *P* < 0.05, *n* = 12). Important features are annotated as retention time and mass-to-charge ratio (*m*/*z*) or compound name. **C)** Extracted ion chromatograms (EIC) of a methanolic tococa leaf extract from *S. littoralis*–damaged leaves analyzed by LC-qTOF-MS. Identified compounds and detected ions are annotated. cps, counts per second (electron multiplier).

**Figure 2.**
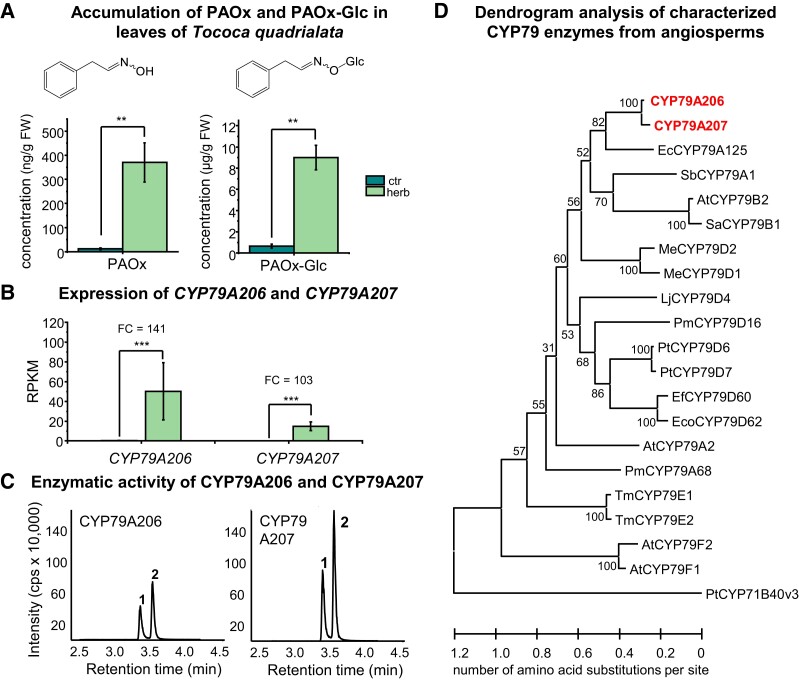
Two CYP79 enzymes are involved in the herbivore-induced formation of PAOx and its glucoside (PAOx-Glc) in leaves of *T. quadrialata*. **A)** Accumulation of PAOx and PAOx-Glc in *S. littoralis*–damaged leaves (herb, 24-h treatment) and undamaged control leaves (ctr). Compounds were extracted with methanol and analyzed using targeted LC–MS/MS. Means ± SEM are shown; *n* = 6. Wilcoxon rank sum test: **P < 0.01. FW, fresh weight. **B)** Expression of *CYP79A206* and *CYP79A207* in herbivore-damaged and undamaged leaves. The leaf transcriptome was sequenced, de novo assembled, and reads mapped to the assembly. FC and *P*-value were calculated via EDGE. Means ± SEM are shown; *n* = 3. EDGE test: ***P < 0.001. RPKM, reads per kilo base per million mapped reads. **C)** Enzymatic activity of CYP79A206 and CYP79A207 with L-phenylalanine. The 2 genes were heterologously expressed in *S. cerevisiae*, and microsomes containing the recombinant enzymes were incubated with NADPH and L-phenylalanine. Products were extracted with methanol and detected using targeted LC–MS/MS. cps, counts per second (electron multiplier); 1, (*E*)-PAOx; 2, (*Z*)-PAOx. **D)** Dendrogram analysis of CYP79A206 and CYP79A207 with characterized CYP79 enzymes from other angiosperms. The tree was inferred with the maximum likelihood method and *n* = 1,000 replicates for bootstrapping. Bootstrap values are shown next to each node. PtCYP71B40v3 was used as an outgroup. Tococa CYP79s are shown in red and bold. At, *A. thaliana*; Ec, *Eucalyptus cladocalyx*; Eco, *Erythroxylum coca*; Ef, *E. fischeri*; Pm, *Prunus mume*; Lj, *L. japonicus*; Me, *Manihot esculenta*; Pt, *Populus trichocarpa*; Sa, *Sinapis alba*; Sb, *Sorghum bicolor*; Tm, *Triglochin maritima*.

Targeted and untargeted metabolome analyses of herbivore-damaged and undamaged leaves of tococa plants grown either in the field or in the greenhouse showed that herbivory by *Spodoptera* spp. led to an induced emission of volatiles and the accumulation of a number of nonvolatile polar compounds (Müller et al. 2022; Fig. 1, A and B; Supplemental Fig. S1). Among the latter metabolites, one of the highest fold changes (FC) was repeatedly found for a feature with *m*/*z* 136.0757 [M+H]^+^. The accurate mass allowed the prediction of the sum formula C_8_H_9_NO (exact mass [M+H]^+^ 136.0757). This formula corresponds, among others, to the molecular formulae of PAOx and phenylacetamide. However, a more detailed analysis of the mass spectrum revealed that this feature likely represents an in-source fragment of a compound with a mass of 297 (*m/z* 298.1284 [M+H]^+^) (Fig. 1C). The mass difference of 162 corresponds to C_6_H_10_O_5_, a neutral loss typical for glucosides (Cabrera 2006). Indeed, incubation of the methanol-extractable fraction of tococa leaf metabolites with a commercially available β-glucosidase resulted in a decrease of the unknown compound and an increase of PAOx (Supplemental Fig. S2C), suggesting that the unknown compound is likely a glucoside of PAOx. Synthesis of PAOx-Glc (Supplemental Figs. S3 to S12) and a comparison of its retention time and fragmentation pattern with those of the unknown compound (Supplemental Fig. S2, A and B) confirmed the latter as PAOx-Glc, a previously undescribed natural product. The synthetic standard further allowed the quantification of PAOx-Glc in herbivore-damaged and undamaged tococa leaves. The amount of PAOx-Glc in *Spodoptera littoralis*–damaged leaves was about 10 times higher than that of PAOx, with concentrations of up to 10 *µ*g/g fresh weight (FW) (Fig. 2A). In contrast, undamaged leaves contained only trace amounts of PAOx and of PAOx-Glc.

### Tococa CYP79A206 and CYP79A207 produce PAOx in vitro from phenylalanine

The biosynthesis of PAOx from phenylalanine in plants is usually catalyzed by CYP79 enzymes, and a TBLASTN search with CYP79A1 from sorghum (*Sorghum bicolor*) (Koch et al. 1995) against the transcriptome of herbivore-damaged tococa leaves revealed 2 full-length genes with high similarity to CYP79A and CYP79B genes from other plants (Fig. 2D). The full-length genes were designated *CYP79A206* and *CYP79A207* according to the P450 nomenclature rules (David Nelson). Both *CYP79A206* and *CYP79A207* were highly expressed in herbivore-damaged leaves but only marginally in undamaged leaves (Fig. 2B; Supplemental Fig. S13). An amino acid alignment of CYP79A206 and CYP79A207 with characterized CYP79 proteins from other plant species confirmed that the tococa CYP79 enzymes contained all conserved regions important for the catalytic activity (Supplemental Fig. S14). The complete ORF of *CYP79A206* and *CYP79A207* were amplified, cloned, and expressed in yeast (*S. cerevisiae*). Incubation of microsomes containing CYP79A206 or CYP79A207 with L-phenylalanine, L-tyrosine, or L-tryptophan in the presence of the cosubstrate NADPH resulted in the formation of (*E*/*Z*)-PAOx, (*E*/*Z*)-*p*-hydroxyphenylacetaldoxime, and (*E*/*Z*)-indole-3-acetaldoxime, respectively, with L-phenylalanine being the preferred substrate (Fig. 2C; Supplemental Fig. S15). In contrast, no conversion of the aliphatic amino acids L-leucine or L-isoleucine to their respective aldoximes was detected (Supplemental Fig. S15). In the absence of NADPH, only marginal activity toward L-phenylalanine was detectable, and when using microsomes prepared from a yeast strain expressing the empty vector, no enzyme activity was observed (Supplemental Fig. S15).

### CYP71E76 converts PAOx to benzyl cyanide in vitro

**Figure 3.**
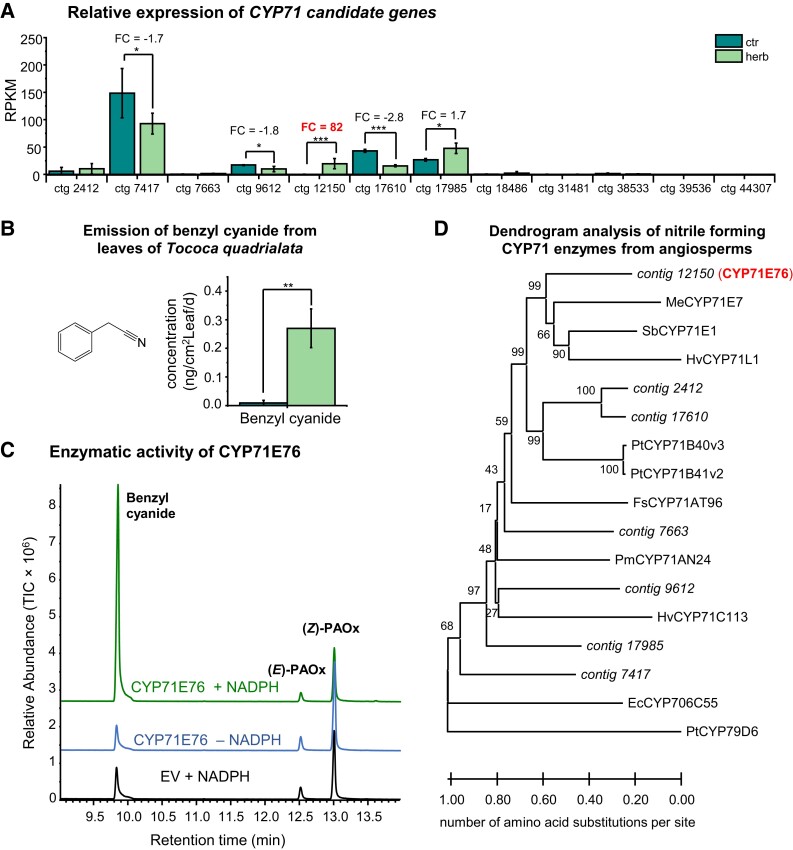
Identification and characterization of CYP71E76. **A)** Expression of *CYP71* candidate genes in herbivore-damaged and undamaged leaves. The leaf transcriptome was sequenced, de novo assembled, and reads mapped to the assembly. FC and *P*-value were calculated via EDGE. Means ± SEM are shown; *n* = 3. EDGE test: *P < 0.05; ***P < 0.001. RPKM, reads per kilo base per million mapped reads. **B)** Emission of benzyl cyanide from *S. littoralis*–damaged leaves (herb) and undamaged control leaves (ctr). Samples were analyzed by GC–MS and quantified by GC-flame ionization detection. Means ± SEM are shown; *n* = 11 to 12. Wilcoxon rank sum test: **P < 0.01. **C)** Formation of benzyl cyanide by CYP71E76. The gene was expressed in *S. cerevisiae*, and microsomes containing the enzyme were used for activity assays with (*E*,*Z*)-PAOx as substrate. The reaction product benzyl cyanide was analyzed using GC–MS. TIC, total ion chromatogram; EV, empty vector. **D)** Dendrogram analysis of CYP71E76 and other expressed CYP71 candidate genes with characterized nitrile-forming CYP71 enzymes from other angiosperms. The tree was inferred with the neighbor-joining method and *n* = 1,000 replicates for bootstrapping. Bootstrap values are shown next to each node. PtCYP79D6 was used as an outgroup. Tococa CYP71 candidates are shown in italics and the characterized one in red and bold. Ec, *E. cladocalyx*; Fs, *F. sachalinensis*; Hv, *Hordeum vulgare* L.; Pm, *P. mume*; Me, *M. esculenta*; Pt, *P. trichocarpa*; Sb, *S. bicolor*.

Since we detected benzyl cyanide in tococa emission (Müller et al. 2022; Fig. 1A; Supplemental Fig. S1), we sought candidate enzymes for the conversion of PAOx to this nitrile. CYP71 and CYP736 enzymes have been implicated in this conversion before, so we conducted a TBLASTN search against the tococa transcriptome using CYP71E1 from sorghum (Bak et al. 1998) as a query. Among the resulting 12 full-length candidate genes, 7 were expressed upon herbivory, but only 1 gene was upregulated upon wounding (Fig. 3, A and D; Supplemental Fig. S13), matching the benzyl cyanide emission pattern (Fig. 3B). Thus, this gene was designated *CYP71E76*, and its complete ORF (Supplemental Fig. S16) was cloned and heterologously expressed in yeast. Microsomes containing the recombinant protein were incubated with different aldoximes and the cosubstrate NADPH. From the 4 compounds tested, CYP71E76 accepted only (*E*/*Z*)-PAOx as substrate and catalyzed the formation of benzyl cyanide in the presence of NADPH (Fig. 3C; Supplemental Figs. S17 and S18). Besides benzyl cyanide, no other products were detected in the assays, neither mandelonitrile as described for hydroxynitrile-forming CYP71s nor its degradation product benzaldehyde (Yamaguchi et al. 2014). Although low levels of benzyl cyanide were also detected in various negative control assays without NADPH, with microsomes from yeast cells expressing the empty vector, or with CYP79A206, these are likely due to thermal dehydration of the aldoxime substrate during high-temperature gas chromatography (GC) injection.

### UGT85A122, UGT85A123, and UGT75AB1 can produce PAOx-Glc in vitro

**Figure 4.**
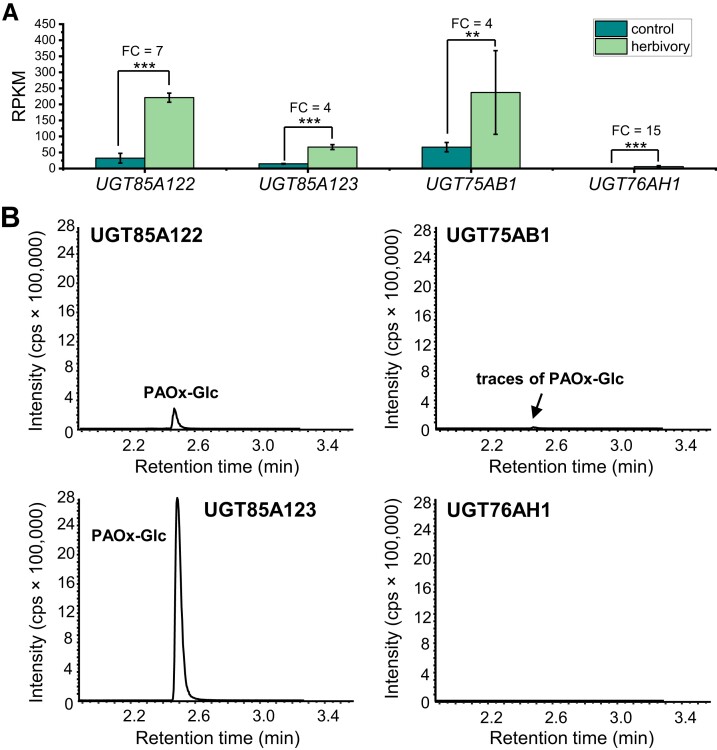
Formation of PAOx-Glc by UGTs from tococa. **A)** Expression of *UGT85A122*, *UGT85A123*, *UGT75AB1*, and *UGT76AH1* in herbivore-damaged (herbivory) and undamaged (control) leaves. The leaf transcriptomes were sequenced, de novo assembled, and reads mapped to the assembly. FC and *P*-values were calculated via EDGE. Means ± SEM are shown; *n* = 3. EDGE test: **P < 0.01; ***P < 0.001. RPKM, reads per kilo base per million mapped reads. **B)***UGT85A122*, *UGT85A123*, *UGT75AB1*, and *UGT76AH1* were heterologously expressed in *E. coli* as His-tag fusions, and purified enzymes were incubated with (*E*,*Z*)-PAOx as substrate. Reaction products were extracted with methanol from the assays and analyzed using targeted LC–MS/MS. cps, counts per second.

As the generation of PAOx-Glc from PAOx requires a UDP-glycosyltransferase (UGT), the tococa transcriptome was searched for UGT genes significantly upregulated (*P* < 0.05, FC ≥ 2) in herbivore-damaged leaves. Five putative UGT85-like genes were induced upon herbivory (Supplemental Table S1). As UGTs of this subfamily are involved in the biosynthesis of cyanogenic glycosides, the 2 most highly expressed UGT85 genes were designated *UGT85A122* (contig_533) and *UGT85A123* (contig_7014) and selected for subsequent studies. However, because UGTs have a broad substrate range and their substrate affinity is difficult to predict from sequence similarity, 2 putative UGT genes from other subfamilies were also included. We chose the candidate gene with the highest FC, a putative UGT of the subfamily 76 designated *UGT76AH1* (contig_23826), and the candidate gene with the highest expression level in wounded samples, encoding a UGT75, designated *UGT75AB1* (contig_20911). An amino acid alignment of UGT85A122, UGT85A123, UGT75AB1, and UGT76AH1 with already characterized UGTs from other plants showed that all selected candidates contained the conserved regions necessary for enzymatic activity (Supplemental Fig. S19). Heterologous expression in *E. coli* and subsequent enzyme assays with various potential substrates revealed that UGT85A122, UGT85A123, and UGT75AB1 were able to glycosylate PAOx (Fig. 4) but with different efficiencies. Unlike UGT75AB1, which accepted a wider range of phenolic substrates, UGT85A122 and UGT85A123 only glycosylated the tested aromatic aldoximes (Supplemental Fig. S20). However, UGT85A122, UGT85A123, and UGT76AH1 also accepted the monoterpene geraniol and the structurally similar fatty acid–derived octen-3-ol as substrates. A comparison of the UGT activities toward (*E*/*Z*)-PAOx revealed that UGT85A123 had the highest conversion rate, 25 times higher than UGT85A122, whereas UGT75AB1 was only able to produce trace amounts of PAOx-Glc (Supplemental Fig. S21).

### Overexpression of *CYP79A206/207*, *CYP71E76*, and *UGT85A123* in *N. benthamiana* enabled reconstitution of the pathways

To verify the activities of the candidate enzymes in planta, we transiently expressed them either alone or in different combinations in *N. benthamiana*. Plants expressing only *CYP79A206* or *CYP79A207* accumulated substantial amounts of PAOx, whereas this compound could not be detected in eGFP-expressing control plants (Supplemental Fig. S22). Coexpression of *CYP79A206* or *CYP79A207* with *CYP71E76*, however, resulted in a significant production of benzyl cyanide and a reduction of PAOx compared to plants expressing only the *CYP79* genes. PAOx-Glc could already be detected in plants expressing only *CYP79A206* or *CYP79A207*, indicating that *N. benthamiana* possesses an intrinsic UGT activity for PAOx. However, coexpression of *CYP79A206* or *CYP79A207* with *UGT85A123* resulted in a significantly increased accumulation of the glucoside and decreased PAOx levels.

### Turnover and spatial distribution of PAOx and PAOx-Glc in tococa leaves after herbivory

**Figure 5.**
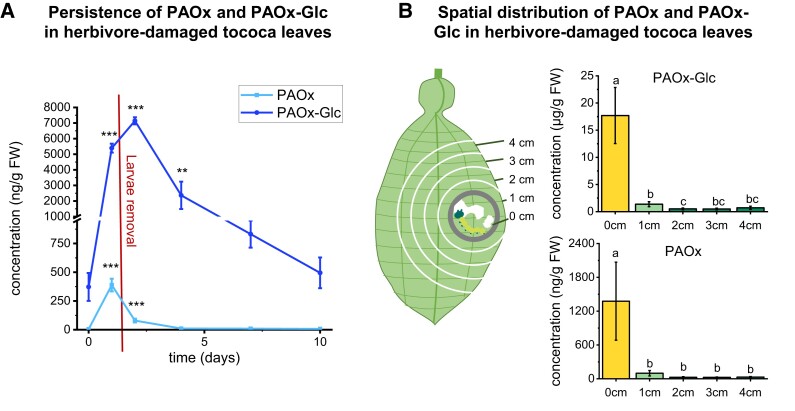
Temporal and spatial distribution of PAOx and PAOx-Glc in herbivore-damaged *T. quadrialata* leaves. **A)** Leaves were exposed to herbivory by *S. littoralis* caterpillars for 24 h, and the accumulation of PAOx and PAOx-Glc was monitored for 10 d. Compounds were extracted with methanol from leaf powder and analyzed using targeted LC–MS/MS. FW, fresh weight. Asterisks indicate significant differences (***P < 0.001, **P < 0.01) to respective control (0 d) based on a one-way ANOVA with log-transformed data (*F*_5,18, PAOx-Glc_ = 12.92, *F*_5,18, PAOx_ = 41.03, *P* ≤ 0.001) and Dunnett's post hoc test. Means ± SEM are shown; *n* = 3 to 5. **B)** A clip cage was installed on tococa leaves, and *S. littoralis* larvae were allowed to feed within the cage for 24 h. Thereafter, leaf discs with the diameter of the cage were excised (“0 cm”) as well as rings of 1 cm width around the wounding site whose outer edge was 1, 2, 3, or 4 cm from the cage (“1 cm” to “4 cm”). Leaf pieces were extracted with methanol and analyzed using LC–MS/MS. FW, fresh weight. Different letters indicate significant differences (*P* < 0.05) between the samples based on linear mixed-effect models with log-transformed data (L-ratio_PAOx_ = 12.821, *P*_PAOx_ = 0.012; L-ratio_PAOx-Glc_ = 12.529, *P*_PAOx-Glc_ = 0.014) and Tukey contrast post hoc tests. Means ± SEM are shown; *n* = 6.

Glucosides often represent storage forms of unstable or toxic natural products (Gachon et al. 2005). To test whether herbivory-induced PAOx-Glc levels in tococa leaves remain stable after herbivory, we measured PAOx-Glc and PAOx in tococa leaves before caterpillar feeding, immediately after 24 h of caterpillar feeding, as well as 1, 3, 6, and 9 d after removal of *S. littoralis* caterpillars that had fed on them. Liquid chromatography–tandem MS (LC–MS/MS) analysis showed that the accumulation of PAOx-Glc continued after herbivore removal, while free PAOx had already returned to almost baseline levels 1 d after herbivory (Fig. 5A; Supplemental Fig. S23). Undamaged control plants showed only trace amounts of PAOx and low levels of PAOx-Glc.

Apart from this temporal dimension, we were also interested in where PAOx and PAOx-Glc accumulate after herbivore feeding. Therefore, we conducted an experiment where the caterpillars had a predefined leaf area to feed on and measured the accumulation of PAOx and PAOx-Glc after 24 h herbivory at the site of damage as well as in more distal parts of the leaves. Interestingly, the accumulation of both compounds was restricted to the site of herbivory (Fig. 5B).

### Mechanical wounding leads to the induction of PAOx-Glc in tococa leaves

**Figure 6.**
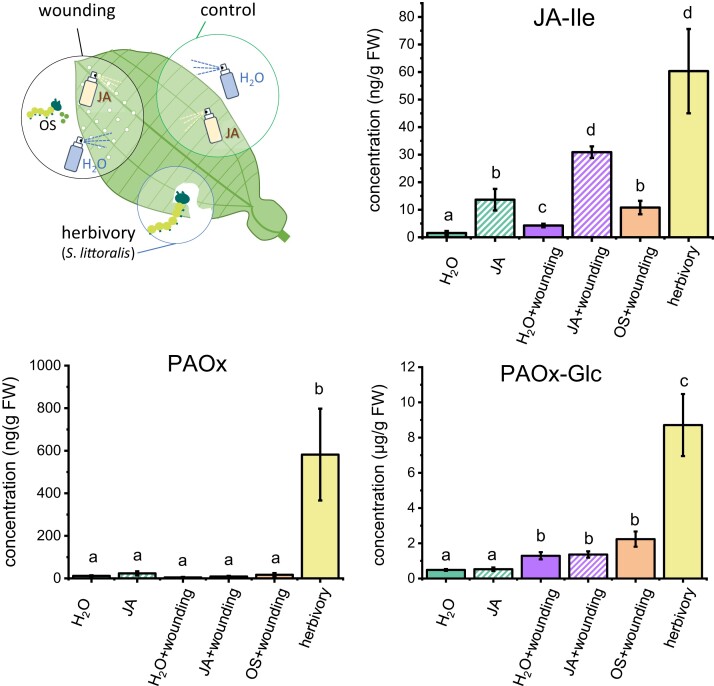
Formation of PAOx and PAOx-Glc in tococa leaves in response to different treatments. Leaves were sprayed with JA, OS collected from *S. littoralis*, or water (H_2_O) after mechanical wounding or without further treatment. *S*. *littoralis* feeding served as a positive control. All leaves were harvested 24 h after the (beginning of the) respective treatment, and the phytohormone JA-Ile, and PAOx and PAOx-Glc contents were quantified via targeted LC–MS/MS. Different letters indicate significant differences (*P* < 0.05) between treatments, based on linear (gls) models (L-ratio_PAOx_ = 27.797, *P*_PAOx_ < 0.001, L-ratio_JA-Ile_ = 73.824, *P*_JA-Ile_ < 0.001) or one-way ANOVA (*F*-value_PAOx-Glc_ = 37.02, *P*_PAOx-Glc_ < 0.001) with log-transformed data and subsequent Tukey contrasts/Honestly Significant Difference post hoc tests. Means ± SEM are shown; *n* = 6 to 11.

Since PAOx and PAOx-Glc are formed in tococa leaves after herbivory (Müller et al. 2022; Fig. 2A), we investigated the internal signals inducing their production. In our previous paper (Müller et al. 2022), we showed that jasmonic acid (JA) and its bioactive isoleucine conjugate JA-Ile (the typical phytohormones involved in plant responses to herbivory) accumulate in tococa leaves upon herbivory, and that spraying of JA resulted in high levels of free amino acids such as phenylalanine, the precursor of PAOx. Here, we compared the effects on PAOx and PAOx-Glc accumulation of other treatments that induce plant defenses, including mechanical wounding and herbivore oral secretions (OS) (Abdul Malik et al. 2020) to learn more about what triggers accumulation of these compounds. As expected, the bioactive JA-Ile accumulated in plants sprayed with JA and to even higher levels when plants were also mechanically wounded (Fig. 6). Mechanical wounding alone also induced JA-Ile but at lower levels than any of the other treatments. Interestingly, the pattern of JA-Ile induction was very different from those of PAOx and PAOx-Glc. None of the treatments resulted in the formation of PAOx-Glc in concentrations as high as those seen after herbivory; however, mechanical wounding resulted in PAOx-Glc levels significantly higher than in control leaves, whereas the application of JA or OS did not alter this response. Interestingly, elevated amounts of PAOx and benzyl cyanide (Supplemental Fig. S24) were only detectable in herbivore-treated samples, not in JA-treated samples, matching the respective gene expression pattern (Supplemental Fig. S24).

### Insect gut protein extracts but not plant protein extracts can hydrolyze PAOx-Glc in vitro

**Figure 7.**
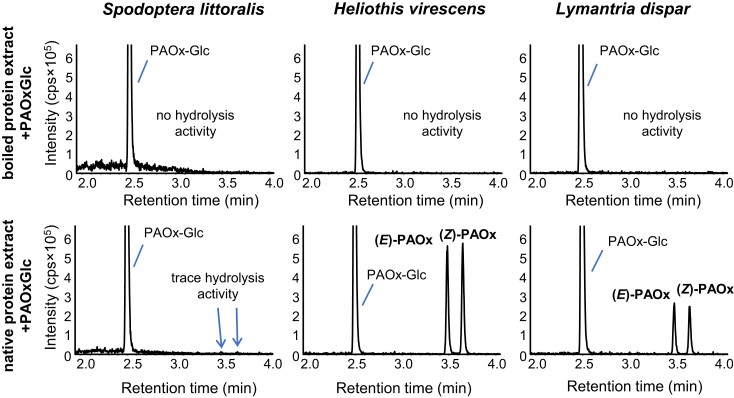
Deglycosylation of PAOx-Glc by gut protein extracts of different insect caterpillars. Gut extracts of *H. virescens*, *S. littoralis*, and *L. dispar* were incubated with PAOx-Glc. Hydrolysis products were extracted with methanol from the assays and analyzed using targeted LC–MS/MS. cps, counts per second.

Aldoximes have been reported to be toxic to mammals, insects, fungi, and microorganisms (Drumm et al. 1995; Bartosova et al. 2006; Irmisch et al. 2013). Therefore, PAOx-Glc stored in tococa leaves upon herbivory may represent a barrier against subsequent herbivore attack. Since the release of toxic aglucones from their glucosides is often catalyzed by enzymes in the insect gut, we examined the capacity of 3 lepidopteran insect species to hydrolyze PAOx-Glc in their guts. Nonboiled (native) and boiled (control) protein extracts prepared from dissected guts of the generalist caterpillars *S. littoralis*, *Lymantria dispar*, and *Heliothis virescens* were incubated with PAOx-Glc, and the release of PAOx was measured using LC–MS/MS. While native (nonboiled) gut protein extracts of *L. dispar* and *H. virescens* were able to hydrolyze the glucoside, the native gut protein extract of *S. littoralis* showed only trace activity toward PAOx-Glc (Fig. 7). Boiling completely inactivated any hydrolytic activity, suggesting that the release of PAOx from its glucoside in gut protein extracts from *L. dispar* and *H. virescens* is enzymatically catalyzed. The protein extracts were also tested for activity toward the cyanogenic glucoside amygdalin, which was efficiently hydrolyzed by all extracts (Supplemental Fig. S25). Interestingly, plant protein extracts made from 3 different PAOx-Glc–producing species (see next paragraph) showed no hydrolysis activity toward PAOx-Glc but efficiently hydrolyzed amygdalin (Supplemental Fig. S26).

### Biotic stresses induce the accumulation of PAOx-Glc in poplar, crape jasmine, and soybean

Various poplar species including *Populus trichocarpa*, *Populus nigra*, *Populus* × *canescens*, and *Populus simonii* × *pyramidalis* have been described to produce volatile and semivolatile aldoximes in response to herbivory (Zeng-hui et al. 2004; Irmisch et al. 2013; Clavijo McCormick et al. 2014). However, whether aldoximes can also be glycosylated in these species was unclear. In the present study, LC–MS/MS analysis of *L. dispar* and *Chrysomela populi*–damaged *P. trichocarpa* leaf samples showed that herbivory indeed resulted in PAOx-Glc accumulation (Supplemental Fig. S27).

Crape jasmine (*Tabernaemontana divaricata*) is a medicinal plant known for its indole alkaloids. While the biosynthesis of these compounds has been studied in detail, studies on other metabolites in the plant are scarce. When we analyzed leaves of crape jasmine after *S. littoralis* feeding, we found a very high accumulation of PAOx in herbivore-treated leaves along with increased levels of PAOx-Glc (Supplemental Fig. S27).

Since the phytoalexin camalexin, which is derived from indole-3-acetaldoxime, accumulates upon pathogen infection in Arabidopsis (*Arabidopsis thaliana*) (Glawischnig 2007) and the aldoxime-producing CYP79B1 is induced in Arabidopsis in response to this treatment (Lemarie et al. 2015), we hypothesized that aldoximes may also be produced as pathogen defenses in other species. Therefore, a soybean (*Glycine max*) cell culture was treated with a crude elicitor fraction, a branched β-glucan cell wall component, from the oomycete *Phytophthora sojae*, and aldoxime formation was analyzed. Interestingly, elicitor-treated soybean cells also produced both PAOx-Glc and free PAOx, while untreated controls showed no accumulation of these compounds (Supplemental Fig. S27).

### PAOx negatively affects the growth of plant pathogenic bacteria

Since aldoxime metabolites accumulated in soybean in response to pathogens, and the literature has previously suggested a role for aldoximes in defense against pathogens, we challenged bacterial plant pathogens with PAOx in growth inhibition assays. We found that the growth of the gram-positive bacteria *Curtobacterium flaccumfaciens* and *Clavibacter michiganensis* (kindly provided by Dr. Matthew Agler, FSU, Jena) and the gram-negative bacteria *Agrobacterium tumefaciens* and *Pseudomonas syringae* (kindly provided by Dr. Katrin Krause, FSU, Jena) were significantly reduced by PAOx in a concentration-dependent manner (Supplemental Fig. S28). Notably, the lowest PAOx concentration tested that affected bacterial growth was 125 *µ*M (approximately 17 *µ*g/g), which is about 15 times higher than the PAOx concentration around the wound site of tococa leaves damaged by herbivores (Fig. 5B). However, since all bacteria tested were able to hydrolyze PAOx-Glc into free PAOx (Supplemental Fig. S29), the Glc (∼17 *µ*g/g FW, Fig. 5B) likely adds to the total concentration of PAOx faced by bacteria entering the wound site.

## Discussion

Aldoximes are known to be precursors of important defense compounds such as cyanogenic glycosides, glucosinolates, and volatile nitriles (Tapper et al. 1967; Underhill 1967; Andersen et al. 2000; Wittstock and Halkier 2000; Irmisch et al. 2014). Recent work has shown that they can also act as defense compounds themselves (Irmisch et al. 2013). Here, we found that in addition to free PAOx, the glucoside PAOx-Glc accumulates as the dominant form of aldoxime in herbivore-damaged tococa leaves. To the best of our knowledge, this compound has not yet been described in plants, nor have aldoxime glucosides been found in nature to date. The aim of our study was to investigate how PAOx-Glc and related compounds are produced in tococa and what functions they perform in plant defense.

**Figure 8.**
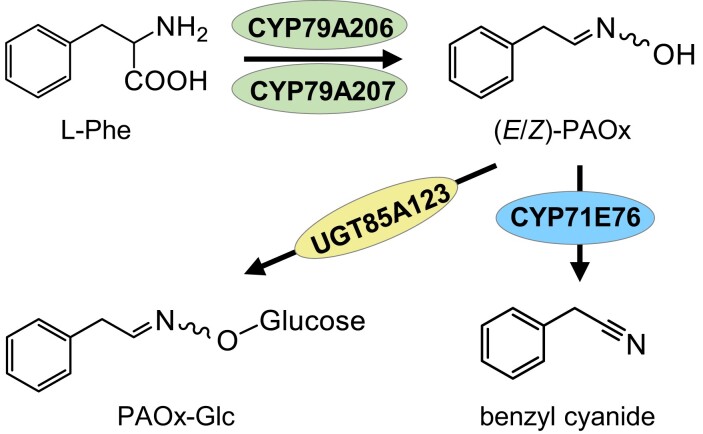
The formation and metabolism of PAOx in *T. quadrialata*.

Enzymes for the biosynthesis of free PAOx and volatile benzyl cyanide have so far only been discovered in a few plants, including poplar (Irmisch et al. 2013; Irmisch et al. 2014), coca (*Erythroxylum coca*) (Luck et al. 2016), maize (*Zea mays*) (Irmisch et al. 2015), and giant knotweed (*Fallopia sachalinensis*) (Yamaguchi et al. 2016). Homology-based searches allowed us to identify candidate enzymes in tococa, and in vitro activity assays and overexpression of *CYP79A206*, *CYP79A207*, and *CYP71E76* in *N. benthamiana* confirmed their role in the formation of PAOx and benzyl cyanide (Fig. 8). Analogous to other studies on herbivore-induced aldoxime formation, the accumulation of PAOx in tococa was strongly associated with the expression of the *CYP79* gene(s) (Irmisch et al. 2013, 2015; Luck et al. 2016; Liao et al. 2020). Similarly, most of the benzyl cyanide–producing CYP71s characterized to date showed an expression pattern that was strongly associated with the emission of this volatile nitrile (Irmisch et al. 2014; Yamaguchi et al. 2016; Liao et al. 2020).

Like other glycosyltransferases accepting aldoxime-related compounds as substrates (Jones et al. 1999; Takos et al. 2011; Knoch et al. 2016), UGT85A122 and UGT85A123 identified in this study also belong to the UGT subfamily 85 (Supplemental Fig. S30). Although UGT85A122 and UGT85A123 were able to glycosylate PAOx in vitro, we conclude from their expression patterns, in vitro characterization, and reconstitution of PAOx-Glc formation in *N. benthamiana* that UGT85A123 is mainly responsible for the accumulation of PAOx-Glc in wounded tococa leaves, whereas UGT85A122 may play only a minor role (Fig. 8).

Interestingly, a deeper phylogenetic analysis suggests that UGT85A122 and UGT85A123 are not directly related to other UGTs involved in PAOx and nitrile glucosylation (Supplemental Fig. S31), indicating the possibility of an independent evolution of this activity. However, since the bootstrap values are at times very low, this interpretation should be treated with caution. Convergent evolution of aldoxime-metabolizing enzymes in different lineages has already been shown for nitrile- and hydroxynitrile-forming enzymes. Poplar, for example, uses a CYP71 for nitrile formation (Irmisch et al. 2014), while in *Lotus japonicus* and in *Eucalyptus cladocalyx,* a CYP736 and a CYP706, respectively, are involved in nitrile production (Takos et al. 2011; Hansen et al. 2018). In contrast, *CYP79* genes have been found in the genomes and transcriptomes of nearly all sequenced angiosperms and gymnosperms (Irmisch et al. 2013; Luck et al. 2017), and all CYP79 enzymes characterized to date produce aldoximes, suggesting a monophyletic origin of aldoxime formation in seed plants. Notably, ferns also produce aldoximes as intermediates for cyanogenic glycosides (Thodberg et al. 2020), but instead of a CYP79, they use a flavin-dependent monooxygenase for the biosynthesis of PAOx. Thus, aldoxime formation has evolved at least twice independently, but is conserved in angiosperms and gymnosperms, and thus seems to have a general role in spermatophytes.

PAOx has already been shown to act as a toxin against generalist caterpillars (Irmisch et al. 2013), and benzyl cyanide is repellent to herbivorous insects (Irmisch et al. 2014). Given these properties as well as the fact that PAOx(-Glc) and benzyl cyanide are formed specifically after insect herbivory, it is reasonable to assume that they function as defense compounds in tococa. However, since aldoximes are also highly reactive (Grootwassink et al. 1990; Bak et al. 1999; Morant et al. 2007), the question arises as to how plants can protect themselves from potential autotoxic effects. Glycosylation is a typical response for coping with toxic compounds in all organisms (Jones and Vogt 2001). Many plants use glycosylation to inactivate toxic compounds and store them in the vacuole (Gachon et al. 2005). The bioactive aglycones are only released upon tissue or cell damage, as has been described in detail for e.g. glucosinolates and cyanogenic glycosides (Dewick 1984). Thus, the glycosylation of PAOx may represent a self-protection mechanism of the plant against high levels of free aldoxime. We have shown that generalist caterpillars possess glucosidase enzyme activity that can release the toxic PAOx from its glucoside (Fig. 7), while plant protein extracts showed no PAOx-Glc hydrolysis activity (Supplemental Fig. S26). It is therefore likely that PAOx is first formed from its glucoside in the caterpillar gut, where it then acts as a toxin. Since PAOx is relatively unstable (Jones and Vogt 2001) and semivolatile, conversion to the stable and nonvolatile glucoside may also provide an elegant way to prevent loss of this nitrogenous and thus metabolically valuable defense compound by decay or emission. Indeed, our studies on the turnover of PAOx and PAOx-Glc showed that PAOx is produced only during injury and is undetectable shortly after herbivory (Fig. 5). However, PAOx-Glc continues to accumulate in the plant for several days after herbivory. This suggests that the production of PAOx is a direct response to herbivore attack, while the storage of PAOx-Glc may help the plant to protect itself from subsequent insect feeding. It still remains an open question how the production of benzyl cyanide fits into this picture. Future time course experiments at earlier time points analyzing the gene expression pattern of *CYP71E76* and *UGT85A123* together with the accumulation of PAOx-Glc and benzyl cyanide could provide more insight into the (temporal) regulation of these pathways.

Many plant pathogens require a wound site to invade the plant (Savatin et al. 2014). Because herbivory by feeding insects can lead to extensive wounding and concomitant secondary infections, we performed antimicrobial assays with several plant pathogenic bacteria to determine a possible role for PAOx in pathogen defense. Indeed, we found that PAOx reduced the growth of these bacteria. Notably, the concentrations of PAOx active in the in vitro assays were very high compared to those measured in tococa leaves. However, considering the facts that PAOx-Glc and PAOx accumulate exclusively in an area of a few millimeters around the wound site (Fig. 5) and that PAOx-Glc is also deglycosylated by the bacteria (Supplemental Fig. S29), adding to the PAOx levels, the tested PAOx concentrations could be reached, if not in tococa, then at least in other plants (poplar and crape jasmine) with higher PAOx levels. We therefore hypothesize that PAOx and its glucoside play a role not only in defense against caterpillars but also in the prevention of secondary infections by phytopathogenic bacteria and fungi.

Experiments on the inducibility of PAOx and PAOx-Glc formation have shown that mechanical wounding is necessary for PAOx-Glc accumulation and that continuous feeding of caterpillars is required to obtain higher levels of these compounds (Fig. 6), indicating that the production of PAOx and PAOx-Glc are associated with the extent of wounding. This is very similar to the results of a recent study by Liao et al. (2020), which showed that continuous wounding is necessary for the production of PAOx and benzyl cyanide in tea (*Camellia sinensis*) leaves. Interestingly, JA and its bioactive derivatives appear to play little or no role in regulating the formation of PAOx, PAOx-Glc, and benzyl cyanide in tococa (Fig. 6; Supplemental Fig. S24). This is in contrast to previous experiments with coca (Luck et al. 2016), tea (Liao et al. 2020), or giant knotweed (Yamaguchi et al. 2016), in which the formation of aldoximes and nitriles could be induced by an artificial JA treatment. What is in turn consistent with previous results (Mikkelsen et al. 2003; Irmisch et al. 2013, 2015; Luck et al. 2016, 2017) is that the formation of PAOx-Glc and probably benzyl cyanide is mainly controlled by the expression of the *CYP79* gene(s) and the PAOx availability itself. This is particularly evident when comparing the detected metabolite accumulation with the expression pattern of the corresponding enzymes.

Our study showed that the production of PAOx-Glc in response to biotic stress is not restricted to tococa (order: Myrtales, family: Melastomataceae) but could also be detected in poplar (order: Malpighiales, family: Salicaceae), soybean (order: Fabales, family: Fabaceae), and crape jasmine (order: Gentianales, family: Apocynaceae) (Supplemental Fig. S27). Similar to cyanogenic glycosides, glycosylated aldoximes appear to be widespread in the eudicotyledons and not restricted to a particular family or order like other natural products such as salicinoids or glucosinolates. Given our knowledge of the biosynthetic pathway, induction pattern, and mode of action of PAOx and its glucoside, we conclude that their formation represents a widespread and common plant defense response to tissue damage, helping to protect the injured site from further damage. Future studies on the occurrence of these compounds in the plant kingdom and their distribution relative to cyanogenic glycosides may provide interesting insights into the evolutionary relationship of these compounds as well as the ancestral function of aldoximes in vascular plants.

## Materials and methods

### Plants and insects

Tococa (*T. quadrialata*, recently renamed *M. microphysca*) plants were grown from seeds in a glasshouse (see Supplemental Table S2). Experiments were performed with mature plants (ca. 30 cm tall). Egyptian cotton leafworm (*S. littoralis*) larvae were hatched from eggs obtained from Syngenta Crop Protection AG (Switzerland) and reared on an agar-based optimal diet at 23 to 25 °C with 16-h light/8-h dark cycles (Bergomaz and Boppre 1986). Second and third instar larvae were chosen for the herbivory experiments and starved 24 h prior to plant feeding. *N. benthamiana* plants were grown as described in Irmisch et al. (2013). Western balsam poplar (*P. trichocarpa*) trees, gypsy moth (*L. dispar*) larvae, and poplar leaf beetles (*C. populi*) were reared as described in Lackus et al. (2020, 2021), and the insects were starved for 13 h prior to the herbivory experiment. Crape jasmine (*T. divaricata*) trees were kindly provided by Sarah E. O’Connor (Max Planck Institute for Chemical Ecology, Germany) and grown under the same climatic conditions as the tococa plants.

### Tococa herbivore experiment and volatile collection

Tococa plants were randomly divided into treatment and control groups. One leaf of each plant was enclosed in a PET (polyethylene terephthalate) bag (Toppits Bratschlauch, Minden, Germany). Three *S. littoralis* larvae were placed on all leaves of the “treatment” group and allowed to feed for 24 h. Volatiles were collected simultaneously over 24 h using a push–pull system, where charcoal-purified air was pumped into the bag at a flow rate of 0.6 L/min, while 0.4 L/min was pumped out of the system, passing through a 20 mg PoraPak-Q filter (Alltech, IL, USA), which absorbed the volatiles. At the end of the experiment, all leaves were excised and photographed to determine the leaf area, the domatium was removed, and the leaf blade was flash-frozen in liquid nitrogen. All leaf samples were ground in liquid nitrogen before being split in half, and one-half of each sample was lyophilized and the other stored at −80 °C until further processing. To test whether benzyl cyanide is produced by the plant or the caterpillars, a similar experiment was conducted with *S. littoralis* feeding on tococa leaves for 24 h and simultaneous volatile collection, as described above. The caterpillars were then removed from the leaves, the larvae were placed in PET bags, and the volatiles from the wounded leaves and the caterpillars were collected separately for another 24 h using the same parameters as described before.

### Analysis of herbivory-induced volatiles

As described in Müller et al. (2022), volatiles were analyzed via GC–MS and GC–flame-ionization detector (FID) after elution from PoraPak-Q filters using 200 *µ*L dichloromethane containing 10 ng/*µ*L *n*-bromodecane (Sigma-Aldrich, Taufkirchen, Germany) as an internal standard.

### Processing and extraction of leaf samples

For the quantification of benzyl cyanide, 100 mg of ground leaf powder from transiently transformed *N. benthamiana* plants was extracted with 400 *µ*L *n*-hexane containing 10 ng/*µ*L bromodecane as an internal standard. To analyze phytohormones, aldoximes, and unknown semipolar compounds, frozen and ground fresh leaf material (usually 100 mg) was extracted with methanol (100 *µ*L per 10 mg sample) containing internal isotopically labeled phytohormone standards (40 ng/mL D_6_-JA [HPC Standards GmbH, Germany] and 8 ng/mL D_6_-JA-Ile [HPC Standards GmbH]). After mixing for 30 min, the homogenates were centrifuged at 16,000 × *g* for 10 min. Freeze-dried plant material was processed similarly, but the extraction of 20 and 40 mg powder was achieved with 0.5 and 1.0 mL MeOH, respectively.

### HPLC–MS/MS analysis

The analysis of JA and JA-Ile, and untargeted metabolomics of the methanolic extracts of tococa leaves and initial UGT activity assays with various substrates were performed as described in Müller et al. (2022) and Rizwan et al. (2021), respectively (see also Supplemental Tables S3 to S5). Aldoximes and PAOx-Glc concentrations were measured from methanolic extracts as described in Luck et al. (2017) with minor modifications. The exact LC separation and MS analysis parameters are listed in Supplemental Tables S3 and S4. The concentrations of PAOx and PAOx-Glc were determined using external standard curves of authentic standards synthesized as described below. Whenever the concentration of the compounds in the samples was outside the linear range, the extracts were diluted accordingly with MeOH and remeasured. Glucosidase activity was confirmed by analyzing the accumulation of prunasin with the corresponding HPLC–MS/MS method described in Supplemental Tables S3 and S4.

### GC–MS and GC–FID analyses of CYP79 and CYP71 products

The *n*-hexane phase of CYP71E76 activity assays and leaf extracts of transformed *N. benthamiana* were analyzed by GC–MS and GC–FID as described in Müller et al. (2022), with changes only in the temperature ramp (Supplemental Table S6). Compounds were identified by comparison to the authentic standards benzyl cyanide (Merck, Darmstadt, Germany) and PAOx (synthesized as described below). Quantification was achieved by comparing their FID peak area with that of the internal standard, applying equal response factors on a weight basis.

### RNA extraction and cDNA synthesis

Leaf RNA was isolated as described by Müller et al. (2022). For transcriptome sequencing, RNA was extracted from flash-frozen and ground powder of the stem, the roots, and an ant domatium of a control plant as described. cDNA synthesis from 800 ng RNA was performed with the RevertAid First Strand cDNA Synthesis Kit (Thermo Scientific, Schwerte, Germany) using oligo (dT)_18_ primers according to the manufacturer's instructions.

### Transcriptome sequencing and analysis

Total RNA extracted from the leaves of 3 herbivore-treated and 3 undamaged control tococa plants as well as from the stem, the roots, and a domatium of a single undamaged plant was sent to the Max Planck Genome Center, Cologne, Germany, for sequencing (25 M paired-end reads, 150 bp, Illumina HiSeq3000 [San Diego, CA, USA]). Trimming of the obtained reads, de novo assembly, and read mapping were performed using CLC Genomics Workbench (Qiagen Bioinformatics). Specifically, the trimmed reads of a herbivore-treated and an undamaged control leaf transcriptome and of a stem, root, and domatium transcriptome were pooled, randomly reduced by half, and then used to generate a de novo assembly (for details see Supplemental Table S7). Empirical analysis of digital gene expression (EDGE) implemented in CLC Genomics Workbench was performed for gene expression analysis.

### Identification and cloning of putative tococa *CYP79* and *CYP71* genes

Putative tococa *CYP79* genes were identified by TBLASTN analysis using the amino acid sequence of CYP79A1 (GenBank, AAA85440.1) from sorghum (*S. bicolor*) as query and the de novo assembled tococa transcriptome as reference. The complete ORF of the 2 candidate genes *CYP79A206* and *CYP79A207* were amplified from leaf cDNA, PCR products were cloned into the sequencing vector pJET1.2/blunt (Thermo Scientific), and both strands were fully sequenced using the Sanger method. Putative tococa *CYP71* genes were identified by BLAST analysis using the amino acid sequence of CYP71E1 (GenBank: AAC39318) from sorghum against the de novo assembled tococa transcriptome. Considering only full-length sequences expressed in wounded leaves (reads per kilo base per million mapped reads [RPKM] ≥ 0.5) and upregulated upon herbivory (FC ≥ 2, *P* < 0.05) reduced the number of candidates to 1. Hence, the ORF of *CYP71E76* was amplified and cloned as described for the *CYP79* candidate genes (primers: Supplemental Table S8).

### Identification and cloning of putative tococa *UGT* genes

Putative tococa *UGT* genes were identified by searching the de novo assembly for *UGT*s with a high FC and/or expression level upon herbivory. Since only *UGT76AH1* could be inserted into the *E. coli* expression vector pET100/D-TOPO (Thermo Scientific), *UGT85A122*, *UGT85A123*, and *UGT75AB1* were synthesized as codon-optimized sequences (see Supplemental Table S9) and subsequently cloned into the same vector.

### Heterologous expression of CYP79A206, CYP79A207, and CYP71E76 in yeast (*S. cerevisiae*)

The complete ORF of CYP79A206, CYP79A207, and CYP71E76 were cloned into the pESC-Leu2d vector (Ro et al. 2008) as *Not*I-*Pac*I (CYP79A206) or *Spe*I-*Sac*I (CYP79A207 and CYP71E76) fragments. The *S. cerevisiae* strain WAT11 (Pompon et al. 1996), carrying the Arabidopsis (*A. thaliana*) *cytochrome P450 reductase 1*, was used for the heterologous expression of the enzymes following the protocol described in Irmisch et al. (2013).

### Heterologous expression of *UGT* candidate genes in *E. coli*

Chemically competent *E. coli* OneShot BL21Star^TM^ (DE3) cells (Thermo Fisher Scientific) were used for heterologous expression of the target genes. Cells were grown at 25 °C and 220 rpm until an OD_600_ value of 0.6 was reached, induced by the addition of IPTG at a final concentration of 1 mM and subsequently maintained at 18 °C and 220 rpm for an additional 20 h. Bacteria were separated from the culture medium by centrifugation (7 min; 5,000 × *g*; 4 °C) and resuspended in 4 mL of resuspension buffer (Supplemental Table S10). After 30 min incubation on ice, cells were disrupted by 4 freeze-thaw cycles using liquid nitrogen and a 25 °C water bath, respectively. After centrifugation (4 °C, 16,000 × *g*, 45 min), the supernatant was further purified by affinity chromatography with the Ni-NTA Spin Kit (Qiagen), following the manufacturer's instructions. Buffer exchange with Illustra NAP-5 columns (GE Healthcare, Buckinghamshire, UK) yielded 1 mL of the purified proteins in assay buffer (Supplemental Table S10). Protein concentration was determined using the QuickStart Bradford Protein Assay (Bio-Rad, München, Germany).

### In vitro assays of recombinant CYP79A206 and CYP79A207

Twenty microliters of microsomal extracts were incubated with 1 mM substrate (L-Phe, L-Leu, L-Ile, L-Tyr, or L-Trp) and 1 mM NADPH in 75 mM sodium phosphate buffer (pH 7.0) in a total reaction volume of 300 *µ*L at 25 °C and 300 rpm for 2 h before stopping the reaction by adding 300 *µ*L MeOH. After another 60 min incubation on ice and removal of the denatured enzymes by centrifugation (11,000 × *g* for 10 min), the supernatant was transferred to a glass vial and the reaction products were analyzed by targeted LC–MS/MS.

### In vitro assays of recombinant CYP71E76

Twenty microliters of microsomal extracts was incubated with 1 mM substrate (PAOx, acetaldoxime, salicylaldoxime, or benzaldoxime) and 1 mM NADPH in 75 mM sodium phosphate buffer (pH 7.0) in a total reaction volume of 300 *µ*L at 25 °C and 300 rpm. The assay mixture was overlaid with 150 *µ*L *n*-hexane. The reaction was stopped after 2 h by mixing and freezing the samples in liquid nitrogen. The hexane phase was then transferred to a glass vial and the reaction products were analyzed by GC–MS.

### In vitro assays of recombinant UDP-glucosyltransferases

Ten micrograms of the purified protein in assay buffer (Supplemental Table S10) was incubated with 5 mM of UDP-glucose (Sigma, dissolved in ddH_2_O), 1 mM of varying substrates (Supplemental Fig. S20, dissolved in DMSO) or varying amounts of PAOx (substrate affinity assay), and 5 mM β-mercaptoethanol (in assay buffer) in a total volume of 200 *µ*L for 90 min (30 min for substrate affinity assay) at 30 °C under shaking conditions (300 rpm). The reaction was stopped by adding 200 *µ*L MeOH, vigorous mixing, and, after 30 min incubation on ice, the removal of the denatured enzymes by centrifugation (11,000 × *g* for 10 min). The supernatant was transferred into a glass vial and the reaction products were analyzed by targeted LC–MS/MS and untargeted LC–quadrupole time-of-flight (qTOF)–MS.

### Transient expression of candidate enzymes in *N. benthamiana*

Expression was performed as described in Irmisch et al. (2013) with a few modifications. Briefly, the coding regions of *CYP79A206*, *CYP79A207*, *CYP71E76*, and *UGT85A123* were cloned into the pCAMBiA2300U vector (primers: Supplemental Table S8). *A. tumefaciens* strain GV3101 was transformed with either one of these vectors, an *eGFP* construct, or the pBIN:p19 construct. For the *N. benthamiana* transformation, 50 mL of LB selection media (Supplemental Table S10) was inoculated with 5 mL of an overnight culture (220 rpm, 28 °C) and, after overnight growth under the same conditions, the cells were harvested by centrifugation (6,000 × *g*, 15 min, 14 °C) and resuspended in infiltration buffer (Supplemental Table S10) to reach a final OD of 0.6. After shaking for 1 to 3 h at 25 °C, 25 mL of pBIN:p19-containing cultures was mixed with 25 mL cultures carrying *CYP79* or *eGFP*, or with a combination of 12.5 mL *CYP79* and 12.5 mL *CYP71E76* or *UGT85A123*. Three-week-old *N. benthamiana* plants were transformed by syringe infiltration, i.e. the opening of a soaked 1-mL syringe was held against the abaxial side of the leaf and the suspension pressed into the leaves. Four leaves per plant were transformed. Plants were kept in a shaded place for 1 d before being transferred to a location with high light intensity. Leaves were harvested 5 d after transformation, pooled for each plant, and stored at −80 °C.

### NMR spectroscopy

NMR measurements were carried out on a 400 MHz Bruker Avance III HD spectrometer (Bruker Biospin GmbH, Rheinstetten, Germany) for PAOx. Data for PAOx-Glc were measured on a 500 MHz Bruker Avance III HD spectrometer, equipped with a TCI cryoprobe, using standard pulse sequences as implemented in Bruker Topspin ver. 3.6.1. Chemical shifts were referenced to the residual solvent signals of CDCl_3_ (*δ*_H_ 7.26/*δ*_C_ 77.16) and MeOH-*d_3_* (*δ*_H_ 3.31/*δ*_C_ 49.0), respectively.

### Synthesis of PAOx

The synthesis of PAOx from phenylacetaldehyde followed the protocol of Castellano et al. (2011). ^1^H-NMR (400 MHz, CDCl_3_) *δ* ppm: 8.32 (*brs*, 1H, OH), 7.41 to 7.23 (*m*, 5H, Ar-H), 6.94 (*t*, *J* = 5.3 Hz, 1H, NCH), and 3.78 (*d*, *J* = 5.3 Hz, 2H, CH_2_). The chemical shifts were in agreement with published data (Castellano et al. 2011). *E/Z*-configurations were assigned based on chemical shifts of the oxime protons (Karabatsos and Hsi 1967). High-resolution mass spectrometry (HRMS) (electrospray ionization [ESI]-TOF, positive) *m/z*: calculated for C_8_H_10_NO [M+H]^+^ 136.0757, found 136.0757.

### Synthesis of PAOx-Glc

PAOx-Glc was synthesized from *O*-β-D-glucopyranosyl-oxyamine (Lagnoux et al. 2005; Amano et al. 2018) following a published method (Tejler et al. 2005). To *O*-β-D-glucopyranosyl-oxyamine (3.8 mg, 0.019 mmol) in tetrahydrofuran (160 *µ*L) was added benzoacetoaldehyde (2.7 mg, 0.22 mmol), H_2_O (160 *µ*L), and aqueous 0.1 M HCl solution (19 *µ*L). The reaction mixture was stirred overnight. The mixture was neutralized with aqueous 0.1 M NaHCO_3_ solution and concentrated in vacuo. The residue was purified by short-path chromatography using a SPE cartridge (CHROMABOND HR-X, 3 mL, 200 mg, MACHEREY-NAGEL, MeOH:H_2_O = 50:50 to 100:0) to give PAOx-Glc (3.29 mg, 0.011 mmol, 58%, *E*:*Z* = 3:1). NMR data are shown in Supplemental Figs. S3 to S12. HRMS (ESI-TOF, positive) *m/z*: calculated for C_14_H_20_NO_6_ [M+H]^+^ 298.1285, found 298.1285.

### RT-qPCR analysis

Reverse transcription quantitative PCR (RT-qPCR) was performed to confirm results of the RNA-Seq experiment and to analyze gene expression upon different treatments. Experimental details are given in Supplemental Tables S11 and S12 and the respective Supplemental Figs. S13 and S24.

### Long-term response to herbivory

After a leaf of tococa was wrapped in a perforated plastic bag, 3 *S. littoralis* larvae were placed on the leaf, allowed to feed for 24 h and removed. Leaf samples were harvested right before the treatment (0 d), upon removal of the caterpillars (1 d), or 1 to 9 d after larval removal (2 to 10 d). All leaves were flash-frozen in liquid nitrogen and stored at −80 °C.

### Spatial distribution of PAOx and PAOx-Glc

*S. littoralis* larvae were placed in clip cages (3.8 cm diameter) installed on single tococa leaves and were allowed to feed on the leaves for 24 h. Afterwards, leaf samples were harvested using a stencil that enabled cutting out the clip cage area as well as leaf pieces with a distance of 1 cm to the previous piece (see Fig. 4B). All samples were flash-frozen in liquid nitrogen and stored at −80 °C.

### Induction of PAOx and PAOx-Glc by different simulations of herbivory

To investigate a potential effect of JA on aldoxime production, tococa leaves were sprayed with 1 mL 1 mM JA (1:90 in EtOH/dH_2_O) or with a control solution (1:90 EtOH/dH_2_O). The same experiment was also conducted including a wounding step: utilizing a pattern wheel, tiny holes were punched into the leaves before spraying the JA or control solution. We also included an OS treatment. The OS was collected beforehand from *S. littoralis* larvae (fifth instar) feeding on tococa leaves, centrifuged, and the supernatant diluted 1:1 with dH_2_O. Leaves were wounded with the pattern wheel before 200 *µ*L diluted OS was applied to the adaxial and abaxial side of the leaf, respectively.

After application of the respective solution, leaves were dried for 1 h, before being wrapped in PET bags, to collect volatiles via the before mentioned push–pull system using the same parameters as described above for another 23 h. As a positive control, a 24- h *S. littoralis* feeding treatment with simultaneous volatile collection was performed as described before. All leaf samples were harvested 24 h after the respective treatment, flash-frozen in liquid nitrogen, and stored at −80 °C.

### Crape jasmine, poplar, and soybean (*G. max*) treatments

Clip cages with (treatment) or without (control) 3 *S. littoralis* larvae were placed on randomly selected middle-aged leaves of crape jasmine trees. The caterpillars fed slowly at first, and so they were allowed to feed for 48 h. Then, the leaves were excised, and green and brown parts of the leaves were collected separately in liquid nitrogen. For *P. trichocarpa*, leaves were numbered according to the leaf plastochron index (LPI; Irmisch et al. 2013). Then 10 *L. dispar* caterpillars or 12 *C. populi* beetles were released on *P. trichocarpa* leaves LPI3 to 7, allowed to feed for 24 h, and removed. Leaf material was harvested 8 h after removal, flash-frozen in liquid nitrogen, ground, and 1 part stored at −80 °C, while the other part was lyophilized and stored at 4 °C. Soybean (*G. max* L. cv. Harosoy 63) cell suspension cultures were cultivated and the assay conducted according to Nißler et al. (2022). Briefly, cells were kept in the dark under shaking conditions (110 rpm, 26 °C) and were subcultured in fresh medium every 7 d. For the actual experiment, soybean cell suspension cultures were subcultured after 5 d in fresh medium (6 g cells in 40 mL medium) and grown for 2 d before the suspension culture was carefully transferred to a 24-well plate (1 mL/well) (CELLSTAR 662102, Greiner Bio-One, Kremsmünster, Austria). Ten microliters of raw elicitor (50 mg/mL ddH_2_O) isolated from the cell walls of the oomycete *P. sojae* was added to the treatment group, whereas pure ddH_2_O served as control. The plate was kept in the dark under shaking conditions (100 rpm, RT) for 4 d. Then, the suspensions were transferred to suitable tubes for centrifugation, cells were removed (5000 rpm, 4 °C, 20 min), and the supernatant transferred to a new vial for subsequent chemical analysis.

### Raw protein extracts and β-glucosidase activity assays

After the larvae were immobilized by placing them on ice, the entire gut was extracted from the larvae and rinsed with 0.9% (*w*/*v*) aqueous sodium chloride solution. For protein extraction, 1 mL of prechilled gut extraction buffer (Supplemental Table S10) and 3 metal beads were added to the isolated gut. The tissue was disrupted using 2010 Geno/Grinder (SPEXSamplePrep, Metuchen, NJ, USA) (4 °C, 1,250 rpm, 2 × 1 min), and cell debris was removed by centrifugation (30 min, 13,000 × *g*, 4 °C). The supernatant contained soluble proteins and enzymes. Similarly, proteins were extracted from 100 mg of *S. littoralis*–damaged leaves (frozen or ground) by adding 3 to 4 metal beads and 1 mL gut extraction buffer (soybean or crape jasmine) or tococa protein extraction buffer (tococa, Supplemental Table S10), tissue disruption using 2010 Geno/Grinder, and removal of cell debris (same parameters). β-Glucosidase activity was tested by incubating 30 *µ*L crude protein extract with 30 *µ*L PAOx-Glc (20 *µ*g/mL) in 140 *µ*L assay buffer (Supplemental Table S10) for 2.5 h at 30 °C under shaking conditions (300 rpm). The reaction was stopped by adding 200 *µ*L MeOH. As a positive control, the assay was carried out with 2.5 mM amygdalin (Roth, Karlsruhe, Germany) as substrate. As negative control, the assays were repeated with 30 *µ*L of boiled crude extracts (95 °C, 10 min). β-Glucosidase activity was analyzed by targeted LC–MS/MS. To test for glucosidase activity of the pathogenic bacteria, bacteria were grown under the same conditions as described in the subsequent paragraph for antimicrobial assays in the presence or absence of PAOx-Glc. After 24 h, samples were centrifuged to pellet the cells (4 °C, 10 min, 12,000 × *g*), and the supernatant 1:100 diluted in methanol before analysis via LC–MS/MS.

### Antimicrobial assays

PAOx was tested for antimicrobial effects on *A. tumefaciens*, *P*. *syringae* pv. *syringae*, *C. flaccumfaciens,* and *C. michiganensis*. Varying concentrations of PAOx (5, 2.5, 1.25, 0.6, 0.125, and 0 mM in a 20 *µ*L volume of DMSO/ddH_2_O [1:10, *v*/*v*]) were added to 20 *µ*L of freshly grown bacterial cultures (OD_600_ = 0.025 [*C. flaccumfaciens* and *P. syringae*] to 0.1 [*C. michiganensis* and *A. tumefaciens*]) and 160 *µ*L LB medium in a sterile 96-well microtiter plate. As a blank, 20 *µ*L DMSO:ddH_2_O (1:10, *v*/*v*) was added to 180 *µ*L of LB medium. Bacterial growth was monitored with a SPECTRAmax 250-Photometer (Molecular Devices, San Jose, CA, USA) by measuring the OD_600_ every 30 min for 22 h.

### Phylogenetic analysis

For the construction of phylogenetic trees, amino acid alignments of the putative tococa enzymes and characterized enzymes from other plant species were created using the MUSCLE algorithm (gap open, -2.9; gap extend, 0; hydrophobicity multiplier, 1.5; clustering method, UPGMA) implemented in MEGA6 (Tamura et al. 2013), and tree reconstructions were achieved with MEGA6 using the maximum likelihood algorithm (Jones–Taylor–Thornton [JTT] model) for UGTs and CYP79 or the neighbor-joining algorithm (JTT model) for CYP71 sequences. Bootstrap resampling analyses with 1,000 replicates were performed to evaluate the tree topologies. Amino acid alignments were also generated using the ClustalW algorithm and visualized with BioEdit (https://archive.org/details/bioedit).

### Statistical analyses

Statistical analyses were performed with R (Posit_team 2022). All data were tested for statistical assumptions (normal distribution and homogeneity of variances) using diagnostic plots and were log-transformed when necessary. Depending on whether or not the criteria were met, statistical tests were selected accordingly. Statistical details are provided in the figures (mean ± SEM; significance) and figure legends (*n*, test, significance). Data for the volcano plot (Fig. 1B) were calculated using the MetaboAnalyst package (Pang et al. 2021) after data preprocessing with MetaboScape (Bruker Daltonics). Data were filtered by interquartile range and normalized using the Pareto algorithm. All results were visualized with OriginPro, version 2019 (OriginLab Corporation, Northampton, MA, USA).

### Accession numbers

Sequence data from this article can be found in the GenBank/EMBL data libraries under accession numbers PRJNA974355 (RNA-Seq data), OQ921381 (*CYP79A206*), OQ921382 (*CYP79A207*), OQ921383 (*CYP71E76*), OQ921379 (*UGT85A122*), OQ921380 (*UGT85A123*), OQ921384 (*UGT75AB1*), and OQ921385 (*UGT76AH1*). Accession numbers of sequences used for phylogenetic analyses are listed in Supplemental Table S13.

## Supplementary Material

kiad448_Supplementary_Data

## Data Availability

The paper does not report original code. The data that support the findings of this study are available in the supplementary material of this article. Any additional information will be provided by the corresponding author upon reasonable request.

## Supplemental data

The following materials are available in the online version of this article.

**Supplemental Figure S1.** Formation of benzyl cyanide in tococa leaves in response to herbivory (see Fig. 1).

**Supplemental Figure S2.** Identification of PAOx-Glc in herbivore-treated tococa leaves.

**Supplemental Figure S3.** The structure of the synthesized PAOx-Glc.

**Supplemental Figure S4.** PAOx-Glc.

**Supplemental Figure S5.** PAOx-Glc.

**Supplemental Figure S6.** PAOx-Glc.

**Supplemental Figure S7.** PAOx-Glc.

**Supplemental Figure S8.** PAOx-Glc.

**Supplemental Figure S9.** PAOx-Glc.

**Supplemental Figure S10.** PAOx-Glc.

**Supplemental Figure S11.** PAOx-Glc.

**Supplemental Figure S12.** PAOx-Glc.

**Supplemental Figure S13.** RT-qPCR of PAOx-Glc and benzyl cyanide biosynthesis genes.

**Supplemental Figure S14.** Amino acid sequence alignment of CYP79A206 and CYP79A207 with representative CYP79 sequences from other plant species.

**Supplemental Figure S15.** Enzymatic activity of CYP79A206 and CYP79A207 with different amino acid substrates.

**Supplemental Figure S16.** Amino acid sequence alignment of CYP71E76 with sequences of other nitrile-forming CYP71s and CYP71E1 from *S. bicolor*.

**Supplemental Figure S17.** Formation of benzyl cyanide by CYP71E76 in vitro.

**Supplemental Figure S18.** CYP71E76 has narrow substrate specificity.

**Supplemental Figure S19.** Amino acid sequence alignment of candidate UGTs and characterized UGTs from *Sorghum* and Arabidopsis.

**Supplemental Figure S20.** Activity of UGT85A122, UGT85A123, UGT75AB1, and UGT76AH1 toward various substrates.

**Supplemental Figure S21.** Substrate affinity of UGT85A122, UGT85A123, and UGT75AB1 toward PAOx.

**Supplemental Figure S22.** Reconstitution of the formation of benzyl cyanide and PAOx-Glc in *N. benthamiana* by overexpression of tococa *CYP79*, *CYP71*, and *UGT* genes.

**Supplemental Figure S23.** Relative temporal distribution of PAOx and PAOx-Glc in herbivore-damaged *T. quadrialata* leaves (see Fig. 5).

**Supplemental Figure S24.** Gene expression of *CYP71E76*, *CYP79A206/207*, and *UGT85A123* and formation of benzyl cyanide in tococa leaves in response to different treatments (see Fig. 6).

**Supplemental Figure S25.** Deglycosylation of amygdalin by gut enzymes of different caterpillars.

**Supplemental Figure S26.** Deglycosylation of amygdalin but not PAOx-Glc by crude enzyme extracts of various plant species.

**Supplemental Figure S27.** Identification of PAOx-Glc in different plant species upon biotic stress.

**Supplemental Figure S28.** Negative effects of PAOx on the growth of several plant pathogenic bacteria.

**Supplemental Figure S29.** Several plant pathogenic bacteria are able to deglycosylate PAOx-Glc.

**Supplemental Figure S30.** Phylogenetic tree of candidate UGTs from tococa.

**Supplemental Figure S31.** Phylogenetic tree of characterized UGTs of the subfamily 85.

**Supplemental Table S1.** UGT candidate genes for the glycosylation of PAOx-Glc.

**Supplemental Table S2.** Growth conditions for *T. quadrialata*.

**Supplemental Table S3.** Chromatographic gradients for the analysis of jasmonates, aldoximes, and prunasin using an LC–triple quad–MS system.

**Supplemental Table S4.** MRM parameters for the LC–MS/MS analysis of jasmonates, aldoximes, and prunasin using an LC–triple quad–MS system.

**Supplemental Table S5.** Nontarget metabolite analysis by LC–ESI–qTOF–MS.

**Supplemental Table S6.** Temperature profile of benzyl cyanide measurements by GC–FID and GC–MS.

**Supplemental Table S7.** Parameters used for the de novo assembly.

**Supplemental Table S8.** Primers used for cloning.

**Supplemental Table S9.** Codon-optimized ORF of tococa UGTs for expression in *E. coli*.

**Supplemental Table S10.** Buffers.

**Supplemental Table S11.** Thermocycler program for RT-qPCR.

**Supplemental Table S12.** List of primers used for RT-qPCR.

**Supplemental Table S13.** Accession numbers of known enzymes that were used for the construction of the phylogenetic trees.

## Dive Curated Terms

The following phenotypic, genotypic, and functional terms are of significance to the work described in this paper:

CYP71 Gramene: AT3G44600
CYP71 Araport: AT3G44600

NADPH CHEBI: CHEBI:16474

aldoximes CHEBI: CHEBI:22307

leaf AmiGo: PO:0025034
