# Supplementary material for: Biosynthesis, herbivore induction, and defensive role of phenylacetaldoxime glucoside
Source: Plant Physiol. 2023 Aug 16;194(1):329–46. doi: 10.1093/plphys/kiad448 (PMC10756763; doi:10.1093/plphys/kiad448)
Supplement: kiad448_Supplementary_Data [file kiad448_supplementary_data.pdf]

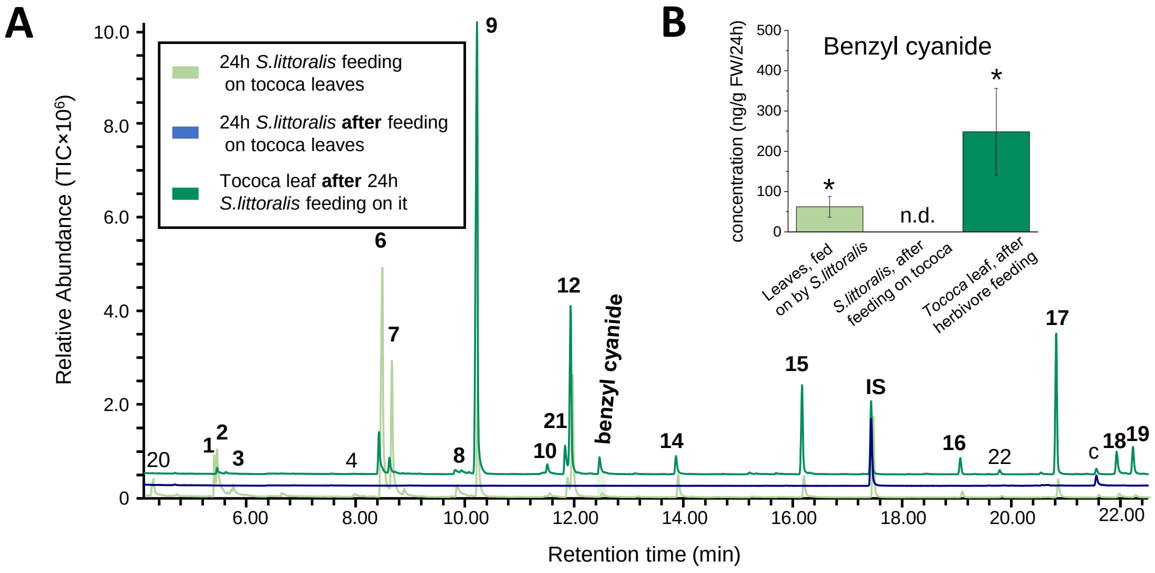

Supplemental Figure S1: **Formation of benzyl cyanide in tococa leaves in response to herbivory (see Figure 1).** 3-4 *S. littoralis* caterpillars were allowed to feed on leaves for 24 h and the volatiles were collected simultaneously (light green). Afterwards, the caterpillars were removed from the leaves, put into another bag and their volatiles collected for 24h (blue). Volatiles of the damaged leaves were collected for another 24 h as well (dark green). Samples were analyzed by GC-MS (**A**) and quantified by GC-FID (**B**). **IS**, internal standard; c, contamination; **1**, 2-hexenal; **2**, 3-hexenol; **3**, 1-hexanol; **4**, 4-oxo-hex-2-enal and benzaldehyde; **6**, 1-octen-3-ol; **7**, octan-3-one; **8**, benzyl alcohol, **9,** β-ocimene; **10**, linalool: **12**, (*E*)-4,8–dimethyl–nonatriene (DMNT); **14**, methyl salicylate; **15**, indole; **16**, (*E*)-β-caryophyllene; **17**, α-farnesene; **18**, nerolidol; **19**, (*E*,*E*)-4,8,12-trimethyl-1,3,7,11-tridecatetraene (TMTT); **20**, 3-hexenal + hexanal; **21**, phenylethyl alcohol; **22**, α-humulene; TIC: total ion chromatogram. Means ± SEM are shown; n = 5. Asterisk indicates significant accumulation of benzyl cyanide (> 0) based on one-tailed t-test, * = p<0.05. The absence of volatiles in the blue chromatogram (only caterpillars after feeding on tococa leaves) indicates that all volatiles detected in the headspace of herbivore-damaged tococa leaves (dark green) are actually produced by the plant and not by the caterpillars.

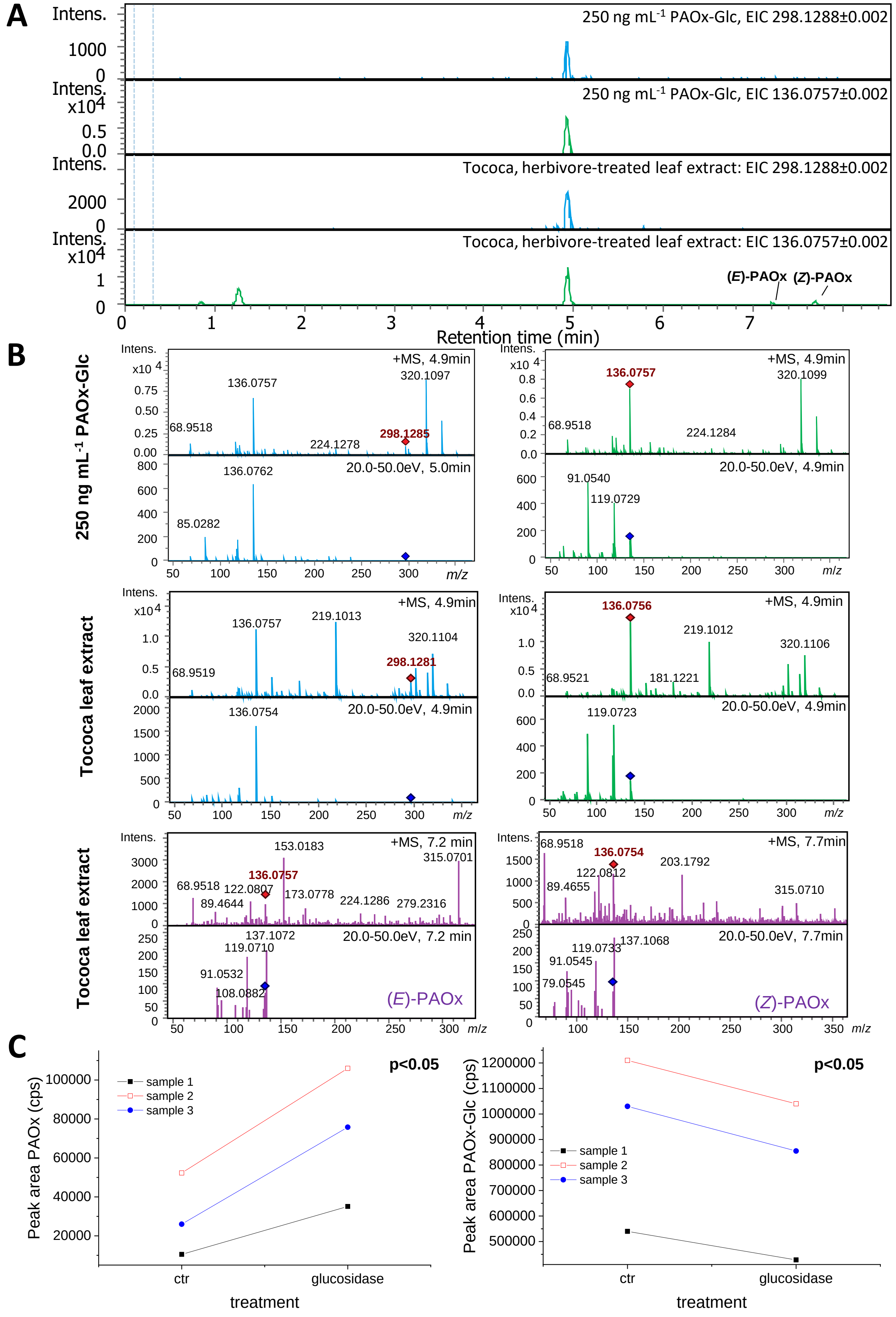

Supplemental Figure S2: Identification of phenylacetaldoxime glucoside (PAOx-Glc) in herbivore-treated tococa leaves. **(A)** Comparison of retention time and **(B)** fragmentation pattern of synthetic PAOx-Glc and plant-derived PAOx-Glc and PAOx (methanolic extract of *Spodoptera littoralis*-damaged leaves) analyzed by LC-qTOF-MS/MS, the upper mass spectrum is the full scan spectrum, the lower spectra are MS2 spectra after CID of *m/z* 298.1285 and *m/z* 136.0757. **(C)** Compounds extracted with 100% methanol from herbivore-damaged leaves were treated with a commercial (Sigma-Aldrich) β-glucosidase from almonds (glucosidase), or only incubated in the reaction buffer (ctr). Paired t-test, n=3. cps: counts per second (electron multiplier).

Phenylacetaldoxime glucoside

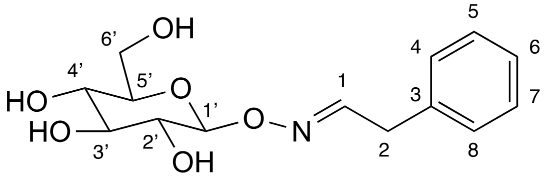

E-isomer

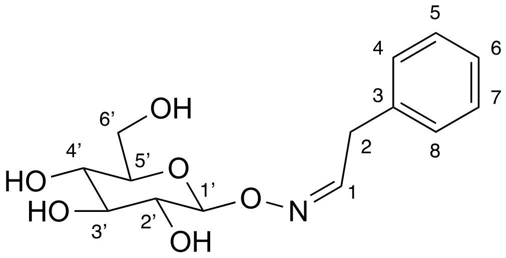

Z-isomer

Supplemental Figure S3: The structure of the synthesized phenylacetaldoxime glucoside. ^{1}H-NMR (500 MHz,MeOH-d_3) for the *E*-isomer: δ ppm: 7.63 (*dd*, *J*= 6.7/6.7 Hz 1H, H-1), 7.30 (*m*, 2H, H-5, H-7), 7.20-7.27 (*m*, 3H, H-4, H-6, H-8), 4.91 (*d*, *J*= 8.2 Hz, 1H, H-1'), 3.87 (*m*, 1H, H-6'), 3.70 (*m*, 1H, H-6'), 3.54 (*d*, *J*= 6.7 Hz, 2H, H-2), 3.30-3.44 (*m*, 4H, H-2', H-3', H-4', H-5'). For the *Z*-isomer (only non-overlapped signals are shown): 6.98 (*dd*, *J*= 5.4/5.4 Hz 1H, H-1), 4.96 (*d*, *J*= 8.0 Hz, 1H, H-1'), 3.77 (*d*, *J*= 5.4 Hz, 2H, H-2). ^{13}C-NMR (125 MHz, MeOH-d_3) *E*-isomer: δ ppm: 153.6 (C-1), 137.2 (C-3), 129.74 (C-4), 129.74 (C-8), 129.67 (C-5), 129.67 (C-7), 127.8 (C-6), 105.6 (C-1'), 78.3 (C-3'), 78.1 (C-5'), 73.56 (C-2'), 71.28 (C-4'), 62.56 (C-6'), 36.5 (C-2). *Z*-isomer: δ ppm: 153.9 (C-1), 137.8 (C-3), 129.8 (C-4), 129.8 (C-8), 129.67 (C-5), 129.67 (C-7), 127.6 (C-6), 105.7 (C-1'), 78.4 (C-3'), 78.1 (C-5'), 73.59 (C-2'), 71.3 (C-4'), 62.63 (C-6'), 33.4 (C-2). *E/Z*-configurations were assigned based on chemical shifts of the oxime protons (Karabatsos & Hsi, 1967).

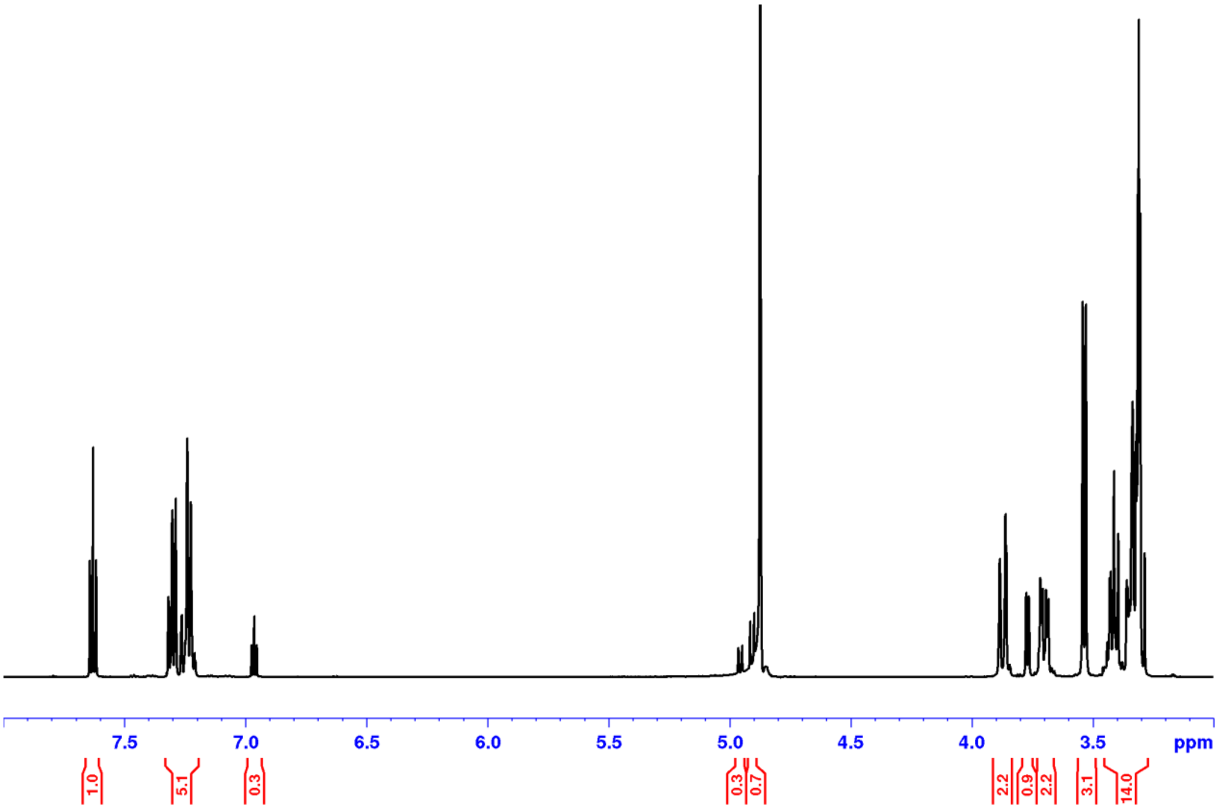

Supplemental Figure S4: Phenylacetaldoxime glucoside, ^{1}H NMR with water suppression, full range in MeOH-d_3

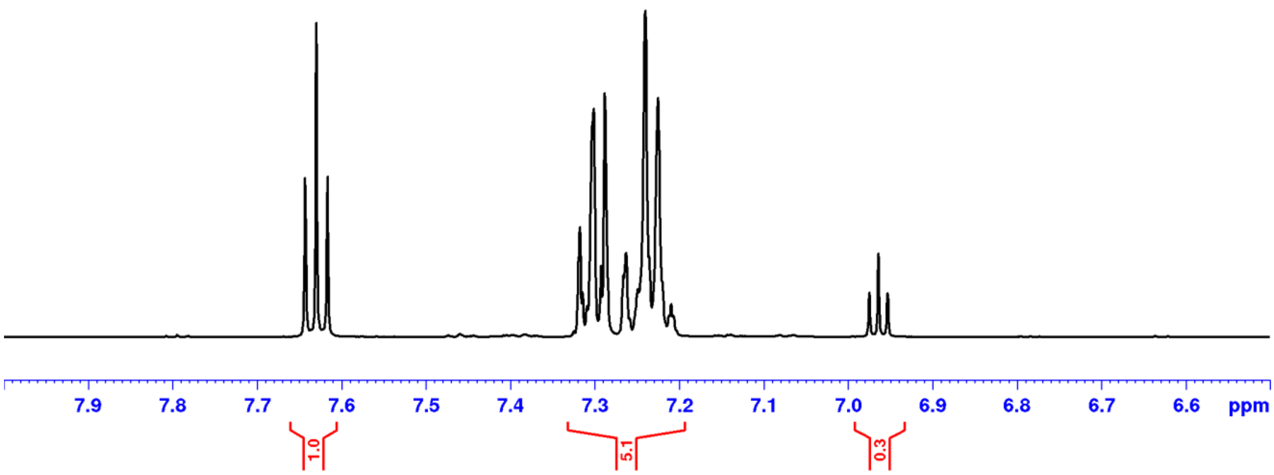

Supplemental Figure S5: Phenylacetaldoxime glucoside, ^{1}H NMR with water suppression, aromatic range in MeOH-d_3

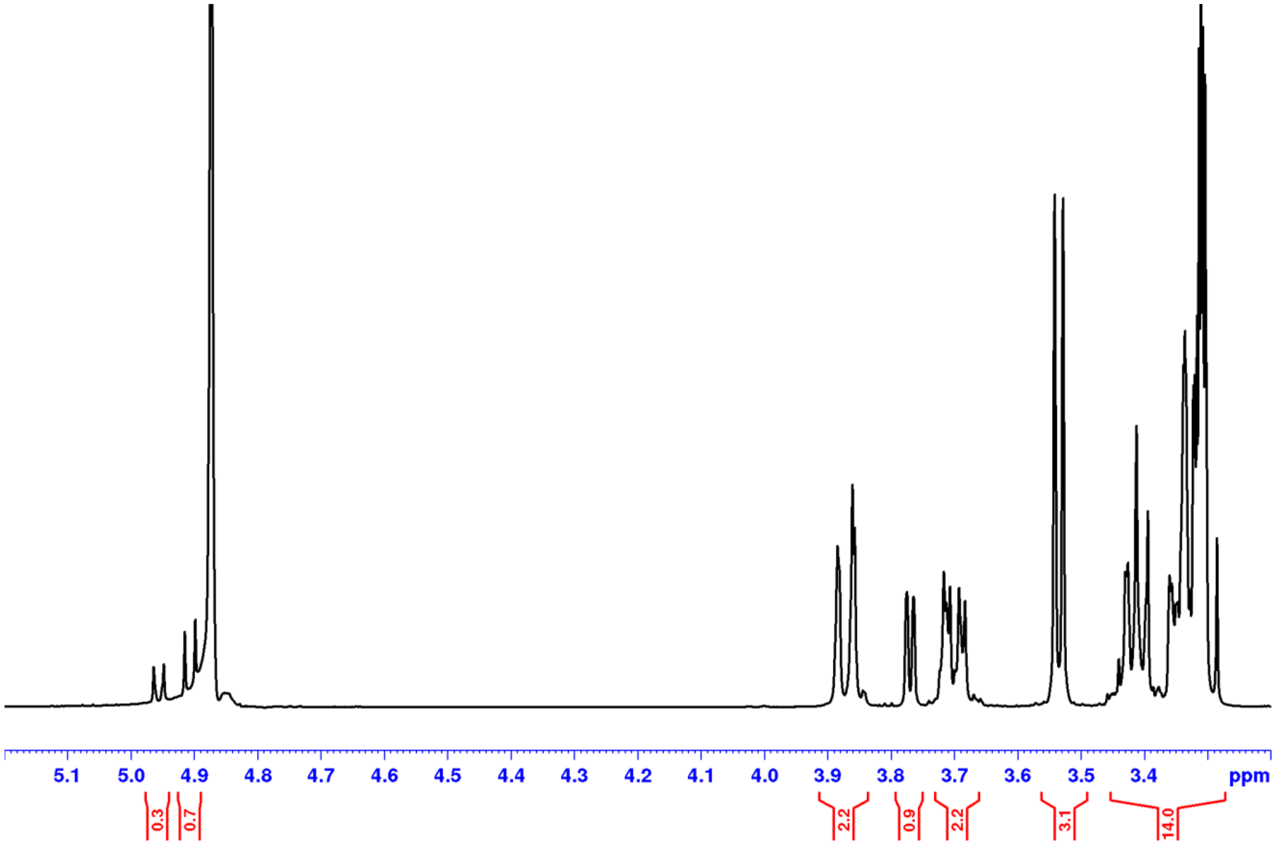

Supplemental Figure S6: Phenylacetaldoxime glucoside, ^{1}H NMR with water suppression, aliphatic range in MeOH-d_3

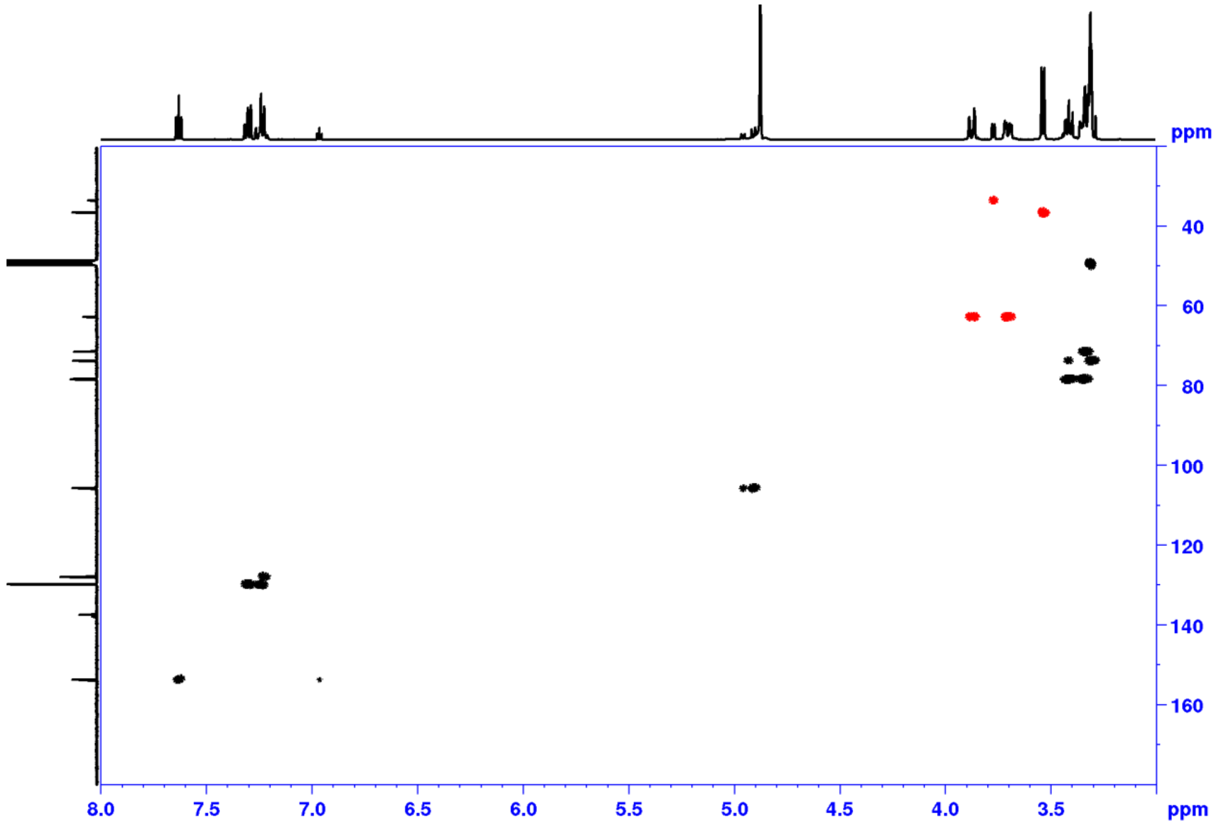

Supplemental Figure S7: Phenylacetaldoxime glucoside, phase sensitive HSQC, full range in MeOH-d_3

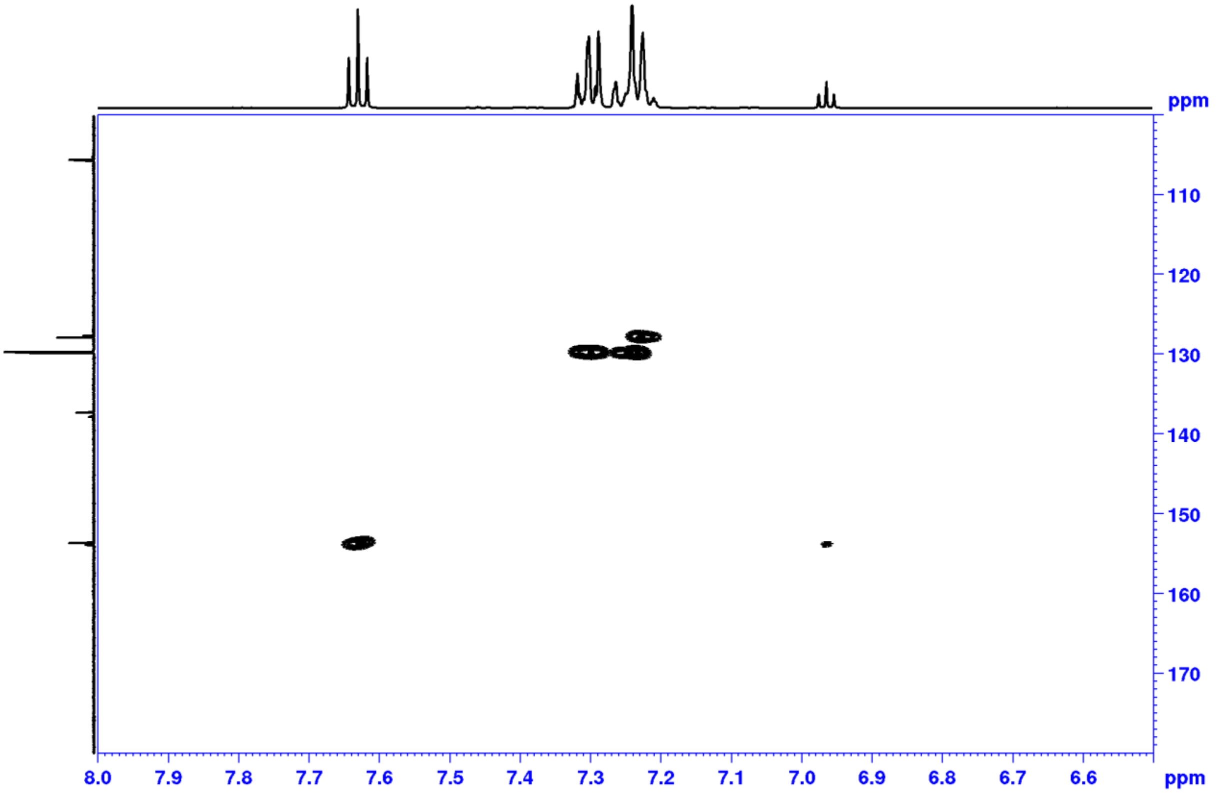

Supplemental Figure S8: Phenylacetaldoxime glucoside, phase sensitive HSQC, aromatic range in MeOH-d_3

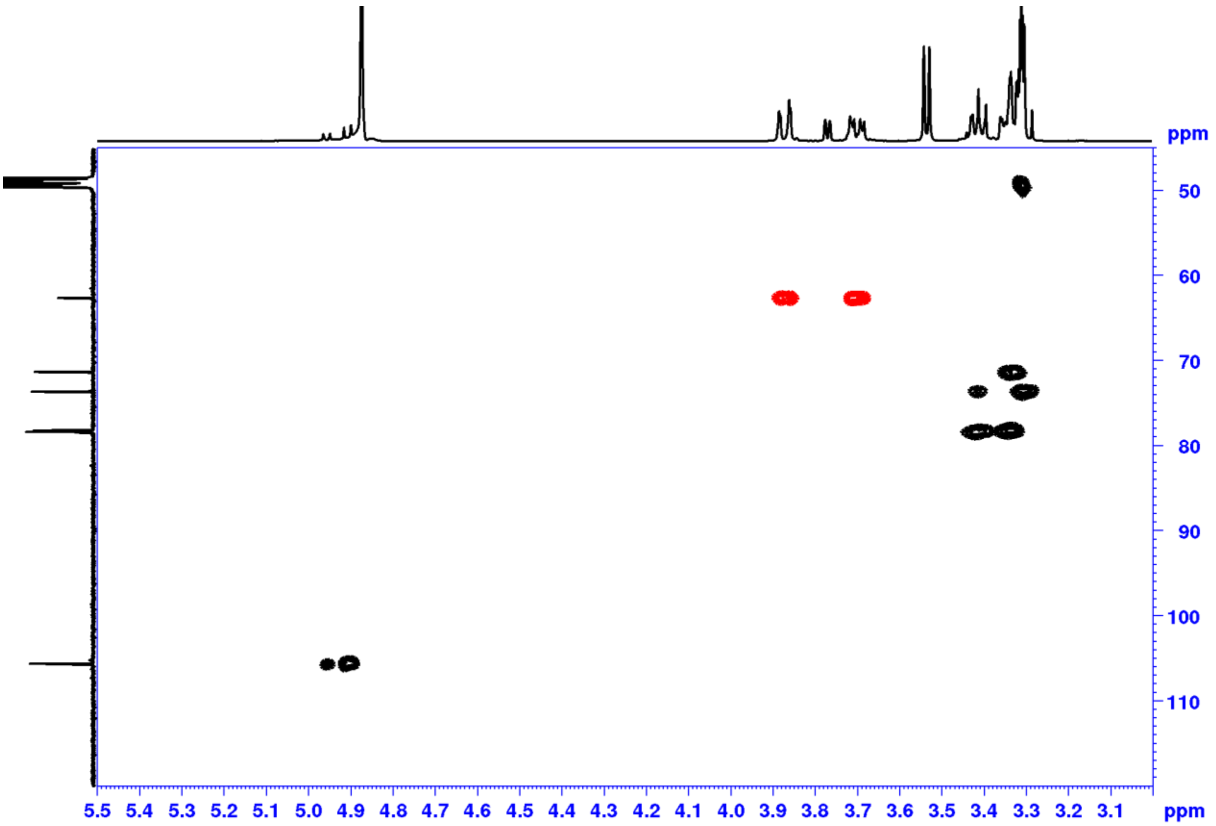

Supplemental Figure S9: Phenylacetaldoxime glucoside, phase sensitive HSQC, aliphatic range in MeOH-d_3. Shaded areas mark impurity and solvent, red: CH_2, black: CH, CH_3

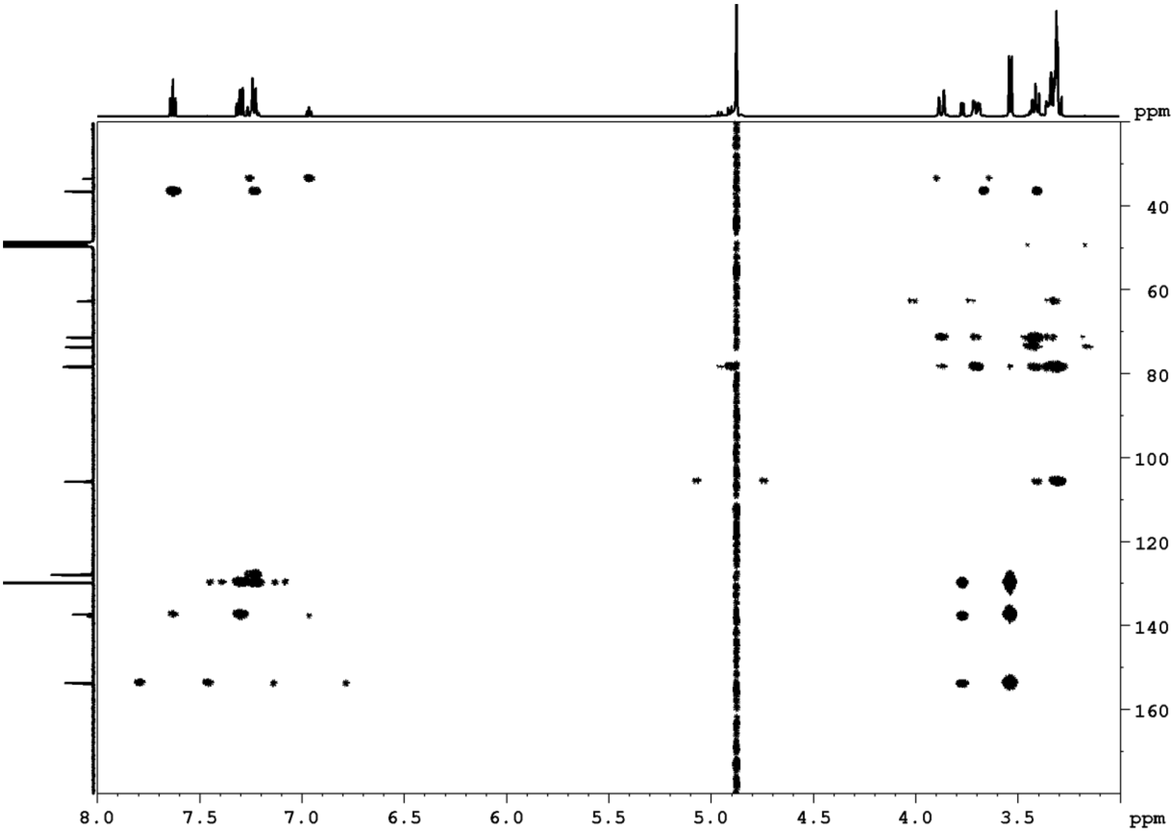

Supplemental Figure S10: Phenylacetaldoxime glucoside, HMBC, full range in MeOH-d_3

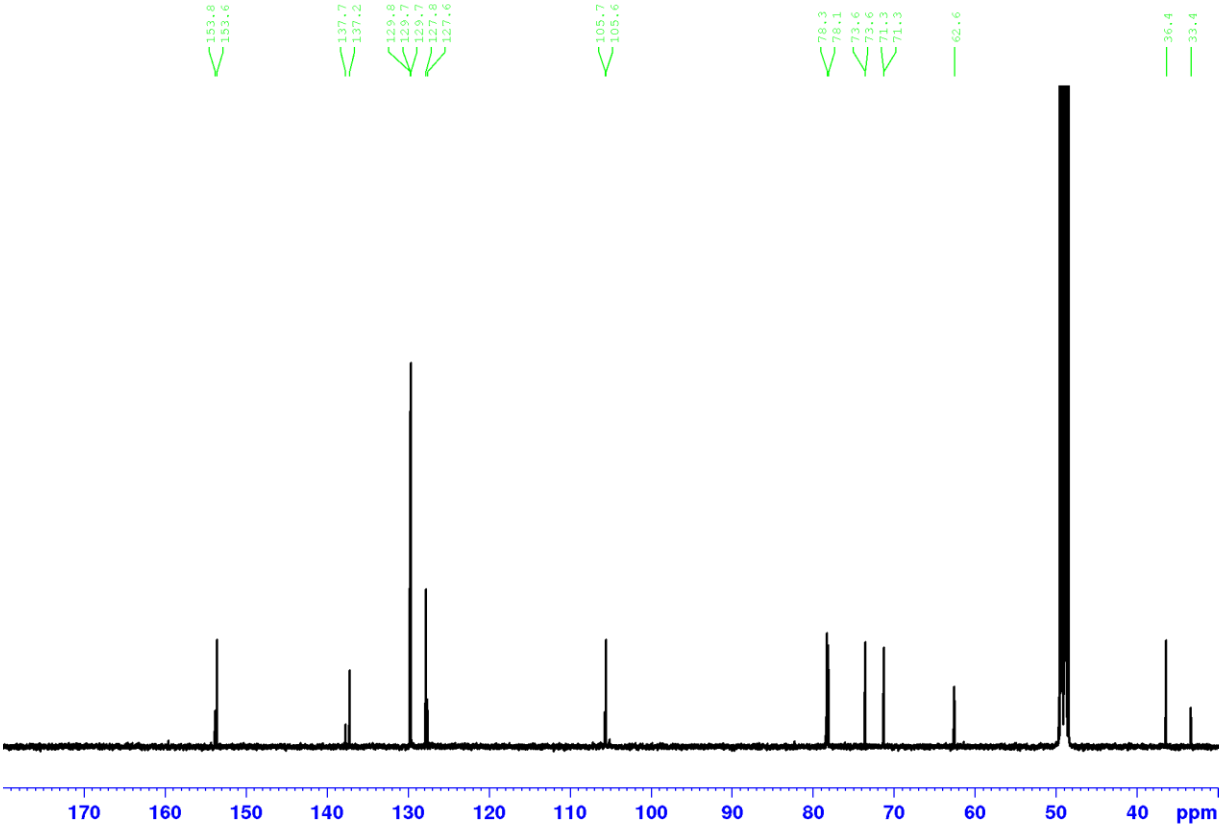

Supplemental Figure S11: Phenylacetaldoxime glucoside, ^{13}C, full range in MeOH-d_3

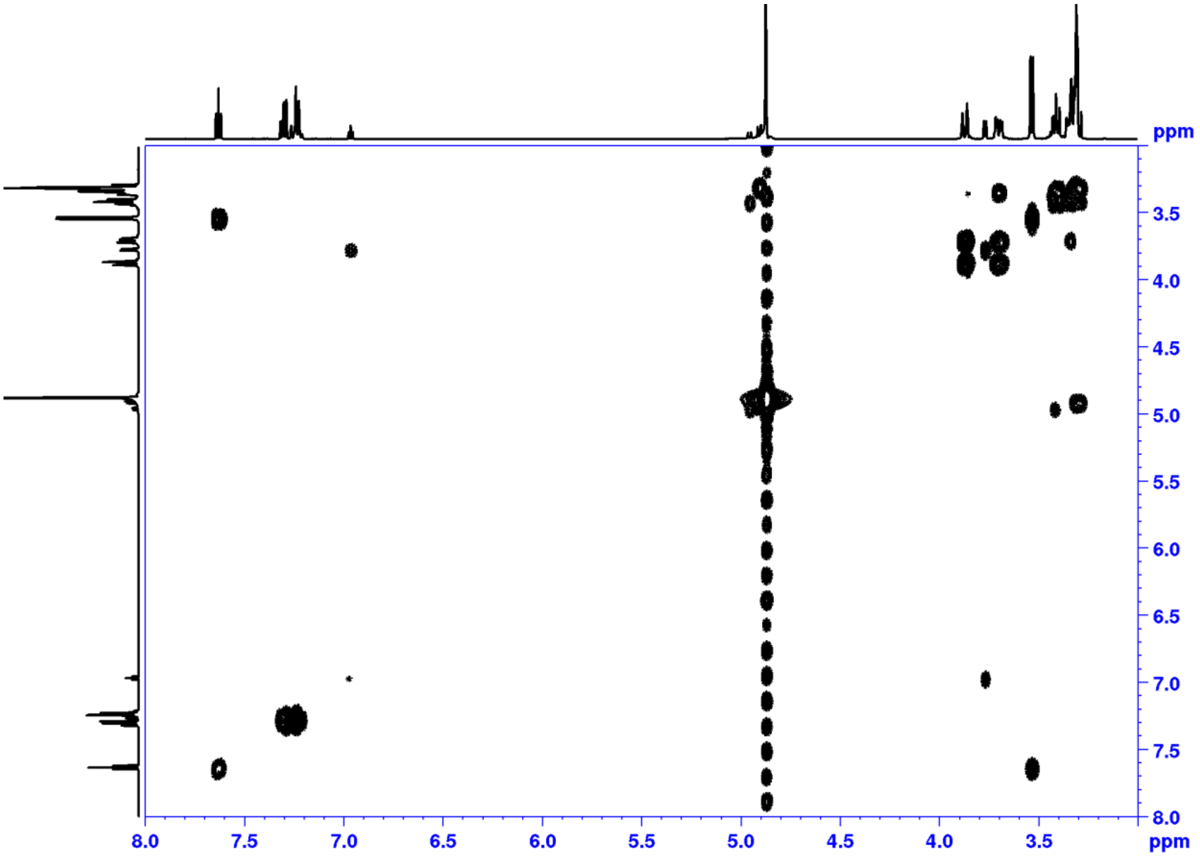

Supplemental Figure S12: Phenylacetaldoxime glucoside, ^{1}H-^{1}H DQF COSY with water suppression. Magnitude mode processed, aliphatic range in MeOH-d_3

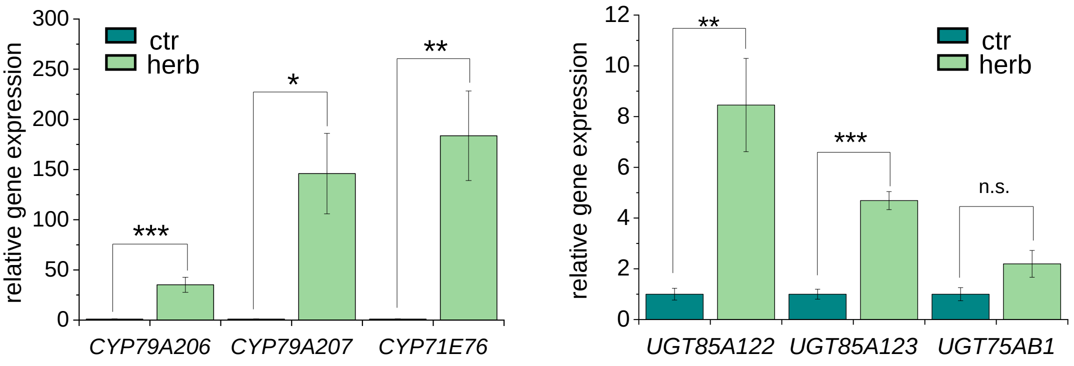

Supplemental Figure S13: **Quantitative reverse transcription PCR (RT-qPCR) of PAOx-Glc and benzyl cyanide biosynthesis genes.** Expression was analyzed in *Spodoptera littoralis*-damaged and undamaged tococa leaves. The RNA of the treated leaves was isolated, transcribed to cDNA, and RT-qPCRs performed using the primers mentioned in Supplemental Table S12 and actin as housekeeping gene. Relative expression was calculated with the ΔΔ Ct method (Pfaffl, 2001). Data are presented as mean ± SEM; Student‘s t-test: n = 5 - 7, * = p<0.05, ** = p<0.01, *** = p<0.001.

Methodology: cDNA synthesis from 800 ng RNA was performed with the RevertAid First Strand cDNA Synthesis Kit (Thermo Scientific) using oligo $(dT)_{18}$ primers according to the manufacturer's instructions. The obtained cDNA was diluted 1:8 with ddH_2O. Primer pairs were designed to amplify the respective gene (Supplemental Table S12), and their specificity confirmed by agarose gel electrophoresis, melting curve, and standard curve analysis. Samples were run in an optical 96-well plate on the CFX96 Touch^{TM} Real-Time PCR System (Bio-Rad) with the Brilliant III Ultra-Fast SYBR Green QPCR Master Mix (Agilent). PCR conditions are given in Supplemental Table S11. All samples were run in duplicates. Normalized fold expression was calculated with the ΔΔCP method (Pfaffl, 2001). As internal calibrator, cDNA from all samples was pooled and run on each plate.

```
                                                                                    PP
TqCYP79A206   MNISASAAIL PNTSSLNATT VFTTIPSFLA ASPLSAFNAF LVSTFVLLYA ILKSQQSSQK SS-------- KQLG--RLPP  70
TqCYP79A207   MNISAYAAIL PNTSSLIATT VIKTKLSFLA ASPLSAFNAF VVSTFVVLYA VLKSQQSSRK IS-------- KQLG--RLPP  70
SbCYP79A1     ---------- MATMEVEAAA ATVLAAPLLS SSAILKLLLF VVTLSYLARA LRRPRKSTTK CSSTTCASPP AGVGNPPLPP  70
PtCYP79D6     ---------- ---------- -----MEYLA PTSFTTLLSF TASLLVLAII LFYFIQSHKN VK-------- ----KHPLPP  43
PtCYP79D7     ---------- ---------- -----MEYLA ATSFTTLLRF PTSLLVLAII LFYFIQSHKN VK-------- ----KHPIPP  43
EcoCYP79D62   ---------- ---------- -----MTYLI LILIMIILVS FQALNVR--- ----CNDKSN RH-------- ----QLQLPP  36
EcCYP79A125   ---------- ---------- --------MS SATLATINAL LLLALVILCS IVKQQLCRKT IR-------- KENNPLPLPP  44

              xP
TqCYP79A206   GPTPWPVLGN LPEMLSNKPV FRWMHRLMEE MNTEIACVRL GSTHVISVTC PKIAAEILKK QDAVFASRPE SMAARSFSDG 150
TqCYP79A207   GPMPWPVLGN LPEMLSHKPV FRWMHRLMEE MDTEIACLRL GSTHVVSVTC PKIAAEILKK QDAVFASRPE SMAARSFSDG 150
SbCYP79A1     GPVPWPVVGN LPEMLLNKPA FRWIHQMMRE MGTDIACVKL GGVHVVSITC PEIAREVLRK QDANFISRPL TFASETFSGG 150
PtCYP79D6     GPKRWPIVGC LPTMLRNKPV YRWIHNLMKE MNTEIACIRL GNVHVIPVIC PDIACEFLKA QDNTFASRPH TMTTDLISRG 123
PtCYP79D7     GPKPWPIVGC LPTMLRNKPV YRWIHNLMKE MNTEIACIRL GNVHVIPVIC PDIACEFLKA QDNTFASRPN TMATDLISSG 123
EcoCYP79D62   GPKPWPVIGC LPQMLRNRPT FAWIHQLMKD MNTEIACIRL GNVHVIPVTC PNIAREFLKS QDEAFSSRPD FMSNKLVSKR 116
EcCYP79A125   GPTPWPVVGC APGMLRNKPV FRWIHRLMKE MNTDIACIRL GSTHVIPVTS PTIAQEFLRK QDSSFASRPV SMASSTFSSG 124

TqCYP79A206   YKTAVLVPHG DQWKKMRRVL TSEIICPARH KWLHDKRVYE ADNLVRYVLA QCGVS----H DVNLRTAARH YSGNLIRNLM 226
TqCYP79A207   YKTAVLVPHG DQWKKMRRVL TSEIICPARH KWLHDKRVDE ADNLVRYVLA QCGVS----R DVNIRTAARH YSGNLIRNLM 226
SbCYP79A1     YRNAVLSPYG DQWKKMRRVL TSEIICPSRH AWLHDKRTDE ADNLTRYVYN LATKAATGDV AVDVRHVARH YCGNVIRRLM 230
PtCYP79D6     YLTTALSPSG DQWNKMKKVL MTHVLSPKKH QWLYSKRVEE ADHLVHYVYN QCKKSVHQGG IVNLRTAAQH YCANLTRKML 203
PtCYP79D7     YLATILSPSG DQWNKMKKVL MTHVLSPKKH QWLYSKRVEE ADHLVHYVYN QCKKSVHQGG IVNLRTAAQH YCANVTRKML 203
EcoCYP79D62   YLTTALSPSG DQWKKMKKIL VTSFLSPAKH QLFYGKRLEE ADNLVSYLYG QCKNNPEKGG LVNVRLATRH YCGNVIRKIV 196
EcCYP79A125   YLTAVLSPYG EQWKKMRRIL VSEVICPARH KWLHDKRVGE ADNLVKYVFN LCKASG---H QVNLRTTTRY YSGNVIRRLM 201

TqCYP79A206   FSKRHFGKGM PDGGPGIEEE EHVEALFTAL GYLYAFCVSD YFPCLEGLDL DGHEKIVKEA TMTLKKYHDP IIDQRIQQWR 306
TqCYP79A207   FSKRHFGKGM PDGGPGIEEE EHVEALFTAL GYLYAFCISD YFPCLEGLDL DGHEKIVKEA TMTLKKYHDP IIDQRIQQWR 306
SbCYP79A1     FNRRYFGEPQ ADGGPGPMEV LHMDAVFTSL GLLYAFCVSD YLPWLRGLDL DGHEKIVKEA NVAVNRLHDT VIDDRWRQWK 310
PtCYP79D6     FNKRFFGEGM KDGGPGFEEE EYVDALFSCL NHIYAFCISD FLPSLIGLDL DGHEKVVMEN HRIINKYHDP IIHERVQQWK 283
PtCYP79D7     FNKRFFGEGM KDGGPGFEEE EYVDALFSCL NHIYAFCISD FLPSLIGLDL DGHEKVVMEN HRIINKYHDP IIHERVQQWK 283
EcoCYP79D62   FNKRNFGEGM KDGGPGIEEK EHIDAIFTIL SYLFSFCISD YMPSLIGLDL EGHEKVLKEN TDIVNKYHDP IIEERIQQWR 276
EcCYP79A125   FNKRYFGQGR QDGGPTIDEE QHVDALFNAL TYLYAFCVSD YFPFLKGLDL DGHEKKVEES ARTLRTLHEP IISERIKRWR 281

                                                                   N P
TqCYP79A206   K--SGDSNNK EPQDLLDVLI ALKDSNGQPS LTSDEIKAQT TEIMMAAIDN PSNVAEWAMA EMINQPELLQ KAVEELDRVV 384
TqCYP79A207   K--SGDSNNT EPQDLLDVLI ALKDSNGRPL LTSDEIKAQT TEIMMAAIDN PSNVAEWAMA EMINRPELLQ KAVEELDRVV 384
SbCYP79A1     S-----GERQ EMEDFLDVLI TLKDAQGNPL LTIEEVKAQS QDITFAAVDN PSNAVEWALA EMVNNPEVMA KAMEELDRVV 385
PtCYP79D6     D-----GAKK DTEDLLDILI TLKDPDGNPL LSKDEIKAQI TEIMVAAVDN PSNACEWAFA EMLNQPEILE KATEELDRVV 358
PtCYP79D7     D-----GAKK DTEDLLDILI TLKDRHGNPL LSKDEIKAQI TEIMVAAVDN PSNACEWAFA EMLNQPEILE KATEELDRVV 358
EcoCYP79D62   N-----GMKD REEDLLDILI ALKDDNGNPL LSIEEIKAQI TEIILATVDN PSNAAEWAIA EMINQPEMMK KAVEELDRVV 351
EcCYP79A125   DDLRSESNEK EPQDLLDVLI MLKDSKGMPV LAPQEVRALT TEIMMAAVDN PSNAVEWAMA EMINQPELIH KATEEIDRVV 361

                                                                                   D
                                                                                FxPER
TqCYP79A206   GKDRLVQESD IPHLNYIKAC ARETFRLHPI APFNVPHVAM SDTTVAGYFI PKGSHMLVSR LGLGRNPKVW DEPLEFRPER 464
TqCYP79A207   GKDRLVQESD IPHLNYIKAC ARETFRLHPI APFNVPHVAM SDATVAGYFI PKGSHMLVSR LGLGRNPKVW DEPLEFRPER 464
SbCYP79A1     GRERLVQESD IPKLNYVKAC IREAFRLHPV APFNVPHVAL ADTTIAGYRV PKGSHVILSR TGLGRNPRVW DEPLRFYPDR 465
PtCYP79D6     GKERLVQESD FAHLNYVKAC AREAFRLHPV APFNVPHVSA ADTTVANYFI PKGSYVLLSR LGLGRNPKVW DEPLKFKPER 438
PtCYP79D7     GKERLVQESD FAHLNYVKAC AREAFRLHPV APFNVPHVSA ADTTVANYFI PKGSYVLLSR LGLGRNPKVW DEPLKFKPER 438
EcoCYP79D62   GRERLVQESD FVKLQYVKAC AREAFRLHPL EPFNIPHVST VDTTVANYFI PKGSHVLLSR MGLGRNPKVW DEPHKYKPER 431
EcCYP79A125   GKERLVQESD IGDLNYIKAC AREAFRLHPV APFNVPHVAM SDTVVAGYRI PKGSHVLLSR IGLGRNPEIW DEPLKFKPDR 441

                I                         K    A
               HL                  S FSTGRRGCIG
TqCYP79A206   HLGDGYTNVV LTEPDLRFIS FSTGRRGCIA ASLGTTMTVM LLARLIQGFS WEKSPQAPGI DLSESEHDLF LVRPLNVHAE 544
TqCYP79A207   HLSDGYTNVV LTEPDLRFIS FSTGRRGCIA ASLGTTMTVM LLARLIQGFS WEKSLQAPGI DLSESEHDLF LARPLNVRAE 544
SbCYP79A1     HLATAASDVA LTENDLRFIS FSTGRRGCIA ASLGTAMSVM LFGRLLQGFT WSKPAGVEAV DLSESKSDTF MATPLVLHAE 545
PtCYP79D6     HLNE-MEKVV LTENNLRFIS FSTGKRGCIG VTLGTSMTTM LFARLLQAFT WSLPPSQSSI DLTIAEDSMA LAKPLSALAK 517
PtCYP79D7     HLNE-MEKVV LTENNLRFIS FSTGKRGCIG VSLGTSMTTM LFARLLQAFT WSLPPRQSSI DLTIAEDSMA LAKPLCALAK 517
EcoCYP79D62   HLSN-GDDVV LIEPELRFIS FSTGRRGCIG VNLGTSMTVM LFARLLQGFS WSAPPGKLAI NLNESKTGLA LAQPLVALAK 510
EcCYP79A125   HIMSDQAEVV LTEPDLRFIS FSTGRRGCIA AMLGTTMTVM LLARLIQGFN WSKPSNLSSI NLAESRDDLF LAEPLVAQAE 521

TqCYP79A206   PRLPVHLYPS F------- 555
TqCYP79A207   PRLPLHLYPS F------- 555
SbCYP79A1     PRLPAHLYPS ISI----- 558
PtCYP79D6     PRLPPQVYPG Y------- 528
PtCYP79D7     PRLPPQVYPG Y------- 528
EcoCYP79D62   PRLPQALYQE LTSCINHS 528
EcCYP79A125   PRLPSHLYLA -------- 531
```

Supplemental Figure S14. Amino acid sequence alignment of CYP79A206 and CYP79A207 with representative CYP79 sequences from other plant species. The alignment was achieved with the ClustalW algorithm using BioEdit. Black shading corresponds to conserved residues, dark grey shades mark residues identical in six of the sequences, and residues with light grey shading are identical in five sequences. Conserved motifs are indicated (see Luck et al, 2016, Bak et al, 2011, Durst et al, 1995).

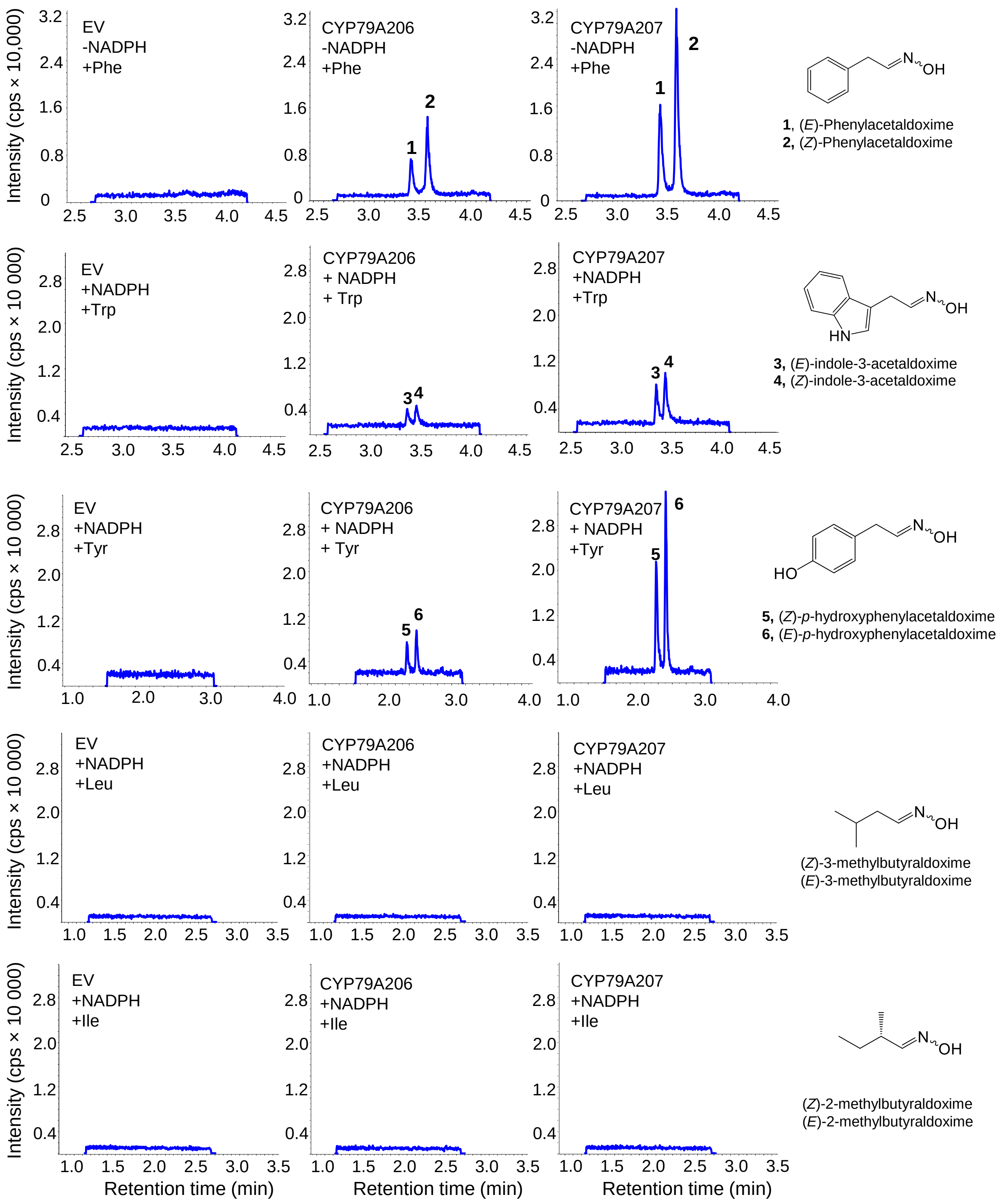

Supplemental Figure S15: **Enzymatic activity of CYP79A206 and CYP79A207 with different amino acid substrates**. The two genes were heterologously expressed in *Saccharomyces cerevisiae* and microsomes containing the recombinant enzymes were incubated with NADPH and either L-tryptophan (Trp), L-tyrosine (Tyr), L-isoleucine (Ile), or L-leucine (Leu). As negative controls, *S. cerevisiae* was transformed with an empty vector (EV) and the respective microsomes were incubated with the substrates (Phe, Trp, Tyr, Ile, Leu) as well. As a second control, microsomes containing the recombinant enzymes were incubated with Phe in the absence of NADPH. Products were extracted with methanol and detected using LC-MS/MS. cps: counts per second (electron multiplier).

```
TqCYP71E76    MSSTIISFPL DN-------F ILLATFALLI IPIIFITFLK R---RNKQQC DGVRLAPSPP TLPIIGNLHQ LG-KLPHRSL  69
PtCYP71B40v3  MALYVVPLWL P--------- -LILLLALLL LFMKKMEVKR Q--------- SEQLLPPSPP KLPILGNLHQ LG-SLPHQSL  60
PtCYP71B41v2  MALYVVPLWL P--------- -LILLLALLL LFMKKMEVKR Q--------- SEQLLPPSPP KLPILGNLHQ LG-SLPHQSL  60
FsCYP71AT96   MHLFLQ---- ---------- -LLLILVLVI SLLFLIQKTR K--------- DVTKQPPGPP RLPLIGNLHQ FPSSCPHLWL  56
SbCYP71E1     MATTATPQLL GGSVPQQWQT CLLVLLPVLL VSYYLLTSRS RNRSRSGKLG GAPRLPPGPA QLPILGNLHL LG-PLPHKNL  79

TqCYP71E76    WELSLRYGPV MMLRLGDVPV LVVSSAEMAK EVLKTHDVDT CSRPASPGTG RLSYDYLDVA FSPYTEYWRE VRKLFMFEMM  149
PtCYP71B40v3  WQLSKKYGPV MLIRLGRIPT VVISSAEAAR EVLKVHDLAF CSRPLLAGTG RLTYNYLDIA FSPYSDHWRN MRKIVTLELF  140
PtCYP71B41v2  WQLSKKYGPV MLIRLGRIPT VVISSAEAAR EVLKVHDVAF CSRPLLAGTG RLTYNYLDIA FSPYSDHWRN MRKILTLELF  140
FsCYP71AT96   HELSKKYGPL IFLKLGFIST LVVSSARMAK QVMKTQDQTF CSRPSLAAQR KLSYNALDVV FAPYGGCWKQ LKKICVLHLF  136
SbCYP71E1     RELARRYGPV MQLRLGTVPT VVVSSAEAAR EVLKVHDVDC CSRPASPGPK RLSYDLKNVG FAPYGEYWRE MRKLFALELL  159

TqCYP71E76    STKRVHSLWH VREAEVDALI GFLRKNAGN- -AVEMNERIF ALTDGIVGTV AFGKMYGRDK FGSKS-FQCV LDEAMNMLAR  226
PtCYP71B40v3  SLKRVQSFRF IREEEVSLLV NFISESSALA APVDLTQKLY ALVANITFRM AYGFNYRGTS FDRDK-FHEV VHDTKAVAGS  219
PtCYP71B41v2  SLKRVQSFRF IREEEVSLLV NFISESSALA APVDLTQKLY ALVANITFRM AYGFNYRGTS FDRDK-FHEV VHDTVAVVGS  219
FsCYP71AT96   SSKKVQSFSS VRQEEVCEMM KEISRLSGEG RVVDLSEMMM SLTCTVICRI AFGKRYDGVT SRCRWSFRDL LKDAQVMLAT  216
SbCYP71E1     SMRRVKAACY AREQEMDRLV ADLDRAAASK ASIVLNDHVF ALTDGIIGTV AFGNIYASKQ FAHKERFQHV LDDAMDMMAS  239

TqCYP71E76    FSAEEVFP-R WGRTIDRLTG LTSRLEKIIK DLDKYYEYVI EEHRKPSRDN EQEIG-DFVD VLLNLQSD-- PKNKLALTDN  302
PtCYP71B40v3  ISADESIP-Y LGWIVDRLTG HRARTERVFH ELDTFFQHLI DNHLKPGRIK EHD---DMVD VLLRIEKEQT ELGASQFTKD  295
PtCYP71B41v2  ISADESIP-Y LGWIVDRLTG HRARTERVFH EVDTFFQHLI DNHLKPGRIK EHD---DMVD VLLRIEKEQT ELGASQFTKD  295
FsCYP71AT96   LSFSDYFP-- GMGWIDKLTG MSARLDKVFM ELNNFYEEII EEHLNTIEPM AKSGQEDIVD VLLKLQRE-- GSLGFDITTD  292
SbCYP71E1     FSAEDFFPNA AGRLADRLSG FLARRERIFN ELDVFFEKVI DQHMDPARPV PDNGG-DLVD VLINLCKE-- HDGTLRFTRD  316

                                        T
                                       TS
TqCYP71E76    HIKAILMDTF IGAISTSSIT MLWAMSELVR NPRVLKKAQD EIRNRIGK-K PR---VESDD MAKLPYLRMV VKETLRLHPP  378
PtCYP71B40v3  NIKAILLNLF MAGVDTSSLT VNWAMAELVR NPRVMKKVQD EVRKCVGN-K GR---VTESD IDQLEYLRMV IKETLRLHPP  371
PtCYP71B41v2  NIKAILLNLF LGGVDTSSLT VNWAMAELVR NPRVMKKVQD EVRKCVGN-K GR---VTESD IDQLEYLRMV IKETLRLHPP  371
FsCYP71AT96   HIKAILMNII VGATETTTAT VVWVMTELMR NPEAMKRLQE ETRTFMMRKK TSDMMIKGED FEKLVYLKAV VKETMRLHPA  372
SbCYP71E1     HVKAIVLDTF IGAIDTSSVT ILWAMSELMR KPQVLRKAQA EVRAAVGDDK PR---VNSED AAKIPYLKMV VKETLRLHPP  393

                                                                             D                                   A   T
                                                                          FxPER F                            PFG SGRRICPG
TqCYP71E76    LALLLPRETI RPCKIGGYDV PARTRVFVNA WGIGRDPRSW ERPEKFDPDR FE-VLERDFK GQDFELIPFG SGRRTCPGMT  457
PtCYP71B40v3  GPLLIPRETM SHCKVSGHNI YPKMLVQINV WAIGRDPRYW KDPEEFFPER FL-DRSIDYK GQSFEYLPFG SGRRICPGMH  450
PtCYP71B41v2  APLLIPRETM SHCKVSGHNI YPKMLVQINV WAIGRDPTYW KDPEEFFPER FL-DSSIDYK GQSFEYLPFG SGRRICPGMH  450
FsCYP71AT96   APLLIPRETI GKCVIEGYEI QEKTLVFVNA WAIGRDPEFW ENPEEFTPER FLSDKAVDFR GQDFELIPFG SGRRICPGMQ  452
SbCYP71E1     ATLLVPRETM RDTTICGYDV PANTRVFVNA WAIGRDPASW PAPDEFNPDR FV-GSDVDYY GSHFELIPFG AGRRICPGLT  472

TqCYP71E76    MGVTTIEFTL ANLLYGFDWK LPEGTKVEDF SMEEEG--GL AIYRKMPLRL VPTKYN---- - 511
PtCYP71B40v3  MGSITMEIIL ANLLYCFDWV FPDGMKKEDI NMEEKAGVSL TTSKKTPLIL VPVNYLQ--- - 507
PtCYP71B41v2  MGFITMEIIL ANLLFCFDWV FPDGMKKEDI NMEEKAGVSL TTSKKTPLIL VPVNYLQ--- - 507
FsCYP71AT96   MGLAMVELAL VNLIGLFDWA LPAGMEEENL DLNMLP--GI AVHKEIPLRL IAMKFCC--- - 507
SbCYP71E1     MGETNVTFTL ANLLYCYDWA LPGAMKPEDV SMEETG--AL TFHRKTPLVV VPTKYKNRRA A 531
```

Supplemental Figure S16: Amino acid sequence alignment of CYP71E76 with sequences of other nitrile-forming CYP71s and CYP71E1 from *Sorghum bicolor*. The alignment was achieved with the ClustalW algorithm using BioEdit. Black shading corresponds to conserved residues, dark grey shades mark residues identical in four of the sequences, and residues with light grey shading are identical in three of the sequences. Conserved motifs are indicated (Durst and Nelson 1995, Bak et al. 2011)

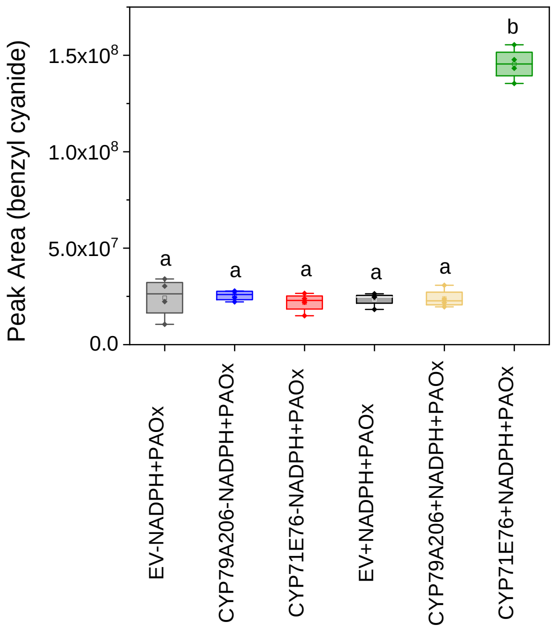

Supplemental Figure S17: Formation of benzyl cyanide by CYP71E76 *in vitro*. Genes were expressed in *S. cerevisiae* and microsomes containing the respective enzymes were used for activity assays with phenylacet-aldoxime (PAOx) as substrate. The hexane phase was analyzed via GC-MS/FID. While benzyl cyanide is always formed from PAOx due to thermal degradation during GC injection, recombinant CYP71E76 in the presence of NADPH was able to generate amounts of benzyl cyanide significantly higher compared to the background. Boxplots (center line, median; box limits, upper and lower quartiles; whiskers, 1.5x interquartile range; open circle, mean). One-Way ANOVA ($F_{5,18}$ = 238.1, $p<0.001$) Tukey HSD *posthoc* test: $p<0.05$; n=4, EV = empty vector.

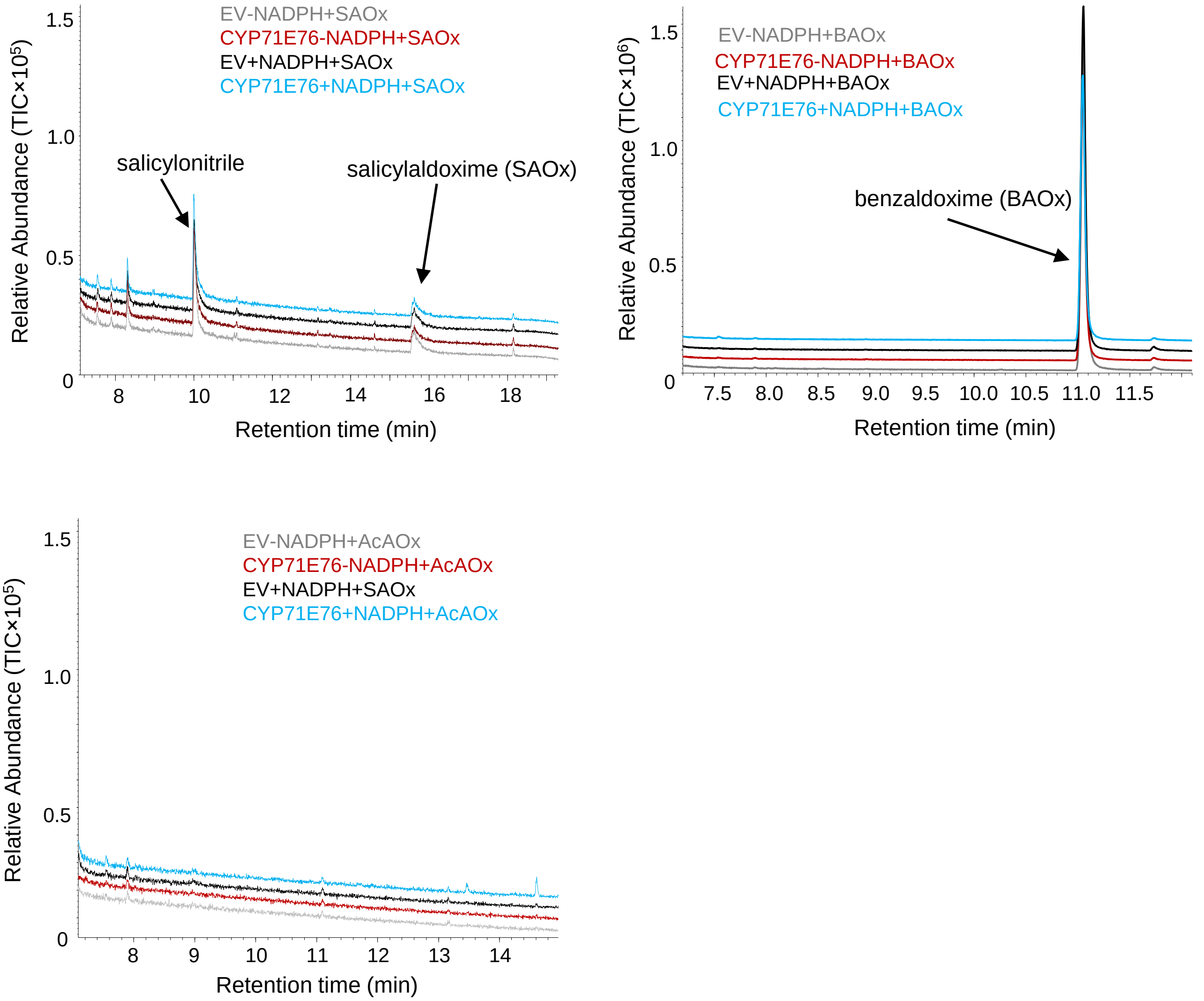

Supplemental Figure S18: CYP71E76 has narrow substrate specificity. The gene was expressed in *S. cerevisiae* and microsomes containing the CYP71E76 were used for activity assays with benzaldoxime (BAOx), salicylaldoxime (SAOx), and acetaldoxime (AcAOx) as potential substrates. The hexane phase was analyzed via GC-MS/FID. Salicylonitrile was always formed from SAOx likely due to thermal degradation during GC injection. The amounts produced in the sample with recombinant CYP71E76 were not higher than the background, indicating no enzyme activity. In addition, no activity was detectable when the microsomes were incubated with BAOx or AcAOx. EV = empty vector.

```
UGT85A122   --MARDS--- -AVEALPGTK PHAIIVPYPA QGHINPLLKL AKILHGRGGF HVTFVNTEYN ARRLLRSRGP TSLDDMPG--  72
UGT85A123   --MSSEQEQS MKTHVHPNPK PHAVFIPYPA QGHINPILKL AKLLHRCHGF HVTFVHTEYN HRRLLRSRGP DSLLGLPG--  76
SbUGT85B1   ---------- MGSNAPPPPT PHVVLVPFPG QGHVAPLMQL ARLLHAR-GA RVTFVYTQYN YRRLLRAKGE AAVRPPATSS  69
UGT75AB1    ---------- -------MAP PNFLLVTFPG QGHINPSLRF AERLIR-IGC HVTFTTALSA RRRMSDSKSP PP--------  54
AtUGT75B1   ---------- -------MAP PHFLLVTFPA QGHVNPSLRF ARRLIKRTGA RVTFVTCVSV FHNSMIANHN KV--------  55
UGT76AH1    MGTTSSQDFS TLNQPNIINY HKVILFPLPF QGHMNSMLQL AQILHSQ-GF SVTVLHIRYN PPRPSDHPG- ----------  68
AtUGT76C1   ---------- -----EKRNE RQVILFPLPL QGCINPMLQL AKILYSR-GF SITIIHTRFN APKSSDHPL- ----------  53

UGT85A122   --FHFETIPD GLPP----VD ADVMQDVPAL SDSLSRTCFE PFVELVSKVN ---QHGG--- PPATCFVYDA MMPFVADAAE  140
UGT85A123   --FRFETIPD GLPPTPEDAS DDVTQDIPSL CDSTSRTCTA PFISLVRRLN A--EEGG--- PPVSCVVFDG AMSFALDAAE  149
SbUGT85B1   ARFRIEVIDD GLSL------ SVPQNDVGGL VDSLRKNCLH PFRALLRRLG Q--EVEGQDA PPVTCVVGDV VMTFAAAAAR  141
UGT75AB1    EGLSFATFSD GYDE----GI KEAELDLDVY MKEITRRGPE TLRELILEKR D-------RG TNFTHIFFTI LMPWAADVAQ  123
AtUGT75B1   ENLSFLTFSD GFDD----GG ISTYEDRQKR SVNLKVNGDK ALSDFIEATK N-------GD SPVTCLIYTI LLNWAPKVAR  124
UGT76AH1    FDFHPVDVPS PVAE-----L EAVEGDTMAL ISLLNVTCVV PFRESLENLM GESSDGGD-- -HVACLITDI CWHFTQAIAD  140
AtUGT76C1   FTF--LQIRD GLSE-----S QTQSRDLLLQ LTLLNNNCQI PFRECLAKLI KPSSDSGTED RKISCVIDDS GWVFTQSVAE  126

UGT85A122   KFGLPAVAFW PPAACAIWGV AQYPKLIEKG LVPLKDSSYL SNG-FLDTVI EWIPGIDN-F RLRDIPTFIQ TTDPNDIMVH  218
UGT85A123   EFGVPGVAYW TPSACGVLAY SYYHKLVEKG LSPLKDSSYL TNG-YLDTVV DWIPGMKKNI CLRDLPSFIR TTDPNDVMVN  228
SbUGT85B1   EAGIPEVQFF TASACGLLGY LHYGELVERG LVPFRDASLL ADDDYLDTPL EWVPGMSH-M RLRDMPTFCR TTDPDDVMVS  220
UGT75AB1    SLGLRSTLVW IQPATVFDIY YYHFNGYDQL IRSSADAAAA DNG---DSRE IRLPGMLP-M TSSYFPSFLA SGNQYHFSLP  199
AtUGT75B1   RFQLPSALLW IQPALVFNIY YTHFMG---- ---------- ------NKSV FELPNLSS-L EIRDLPSFLT PSNTNKGAYD  183
UGT76AH1    EVGIPRMVLR TTSIGSISAF SAVSRLQKNG LLPLPES--- -------QIE EVIPEVAP-L RWKDLP--IV PTR-NPKDFF  206
AtUGT76C1   SFNLPRFVLC AYKFSFFLGH FLVPQIRREG FLPVPDS--- -------EAD DLVPEFPP-L RKKDLSRIMG TSA-QSKPLD  194

UGT85A122   YMIRSVEVSS KKAC-----A VVFNTFDALE ADVLRALKSI YPS-IYTVGP LNLVLDR--Y PEEELTAVGS NLWKEDNSCL  290
UGT85A123   YVIGEIQRTS QKSS-----A LVLNTFDKLE KDVLDALSAI FPS-VYAIGP INLMLDR--V SDEDLDSIGS NLWKEENWCL  300
SbUGT85B1   ATLQQMESAA G-SK-----A LILNTLYELE KDVVDALAAF FPP-IYTVGP LAEVIASSDS ASAGLAAMDI SIWQEDTRCL  293
UGT75AB1    VIKRHFEILN SEKTGTMKPK VLVNTFEELE AEAVKAIDEL NVIPVGPFIP LAFLDEQ--H PTDTSLGGDL FQKSRDLDYI  277
AtUGT75B1   AFQEMMEFLI KET----KPK ILINTFDSLE PEALTAFPNI DMVAVGPLLP TEIFSG---- STNKSV---- --KDQSSSYT  249
UGT76AH1    RLLADIDRAT KACA-----G LISNSFEELE EVKLAESRRS IPIPFFLIGP FHKRFPS--- ----SSTPVA GKLLQDQSCI  274
AtUGT76C1   AYLLKILDAT KPAS-----G IIVMSCKELD HDSLAESNKV FSIPIFPIGP FHIHDVP--- ----ASS--S SLLEPDQSCI  260

UGT85A122   KWLDSQEHGS VVYVNFGSIT VATAEQMTEF AWGLANSRMP FLWVIR---- -PDLVVGES- -AVLPPGFTD ETS-DRCMIS  362
UGT85A123   HWLDSQDLGS VIYVNFGSIT VATKEQITEF AWGLADSKKP FLWVIR---- -PDLVIGES- -AVLPPGFGD ATE-GRGILS  372
SbUGT85B1   SWLDGKPAGS VVYVNFGSMA VMTAAQAREF ALGLASCGSP FLWVKR---- -PDVVEGEE- -VLLPEALLD EVARGRGLVV  366
UGT75AB1    DWLNKQQAAS VIYISFGSLS LFSRPQKEEM AKALIAMGRP FLWVIR---- ---KRMGEEE EEDDKLS-YE EELSKLGMIV  349
AtUGT75B1   LWLDSKTESS VIYVSFGTMV ELSKKQIEEL ARALIEGKRP FLWVITDKSN RETKTEGEEE TEIEKIAGFR HELEEVGMIV  329
UGT76AH1    AWLDKQSPKS TIYVSFGSIA SIDERDFLEI VRGLANSGVP FLWVVR---- -PGSVRGHEW LVTLPPGLLE ----GKGHIV  345
AtUGT76C1   PWLDMRETRS VVYVSLGSIA SLNESDFLEI ACGLRNTNQS FLWVVR---- -PGSVHGRDW IESLPSGFME SLD-GKGKIV  334

                                  PSPG Box                          N          G
UGT85A122   GWCPQEEVLK HPSIGGFLTH SGWNSTLESM SAGVPMVCWP FFADQQTNCW YGKNSWGIGM EIDHDVKRDK VEGMVKELME  442
UGT85A123   GWCPQELVLK HPSIGGFLTH CGWNSMMESV CSGVPVICWP FFAEQQTNCW YGKNAWGIGM EIDNEVKRDE VEGMVRELMD  452
SbUGT85B1   PWCPQAAVLK HAAVGLFVSH CGWNSLLEAT AAGQPVLAWP CHGEQTTNCR QLCEVWGNGA QLPREVESGA VARLVREMMV  446
UGT75AB1    PWCSQVEVLS NPSVGCFVTH CGWNSTSESL VCGVPMVGFP QWSDQQTNAK LVEEVWRTGV QVGKGNGEGV VEAGEIERCL  429
AtUGT75B1   SWCSQIEVLS HRAVGCFVTH CGWSSTLESL VLGVPVVAFP MWSDQPTNAK LLEESWKTGV RVRE-NKDGL VERGEIRRCL  408
UGT76AH1    KWAPQREVLA HPSVGAFWTH NGWNSTTEAI CEGVPMLSSP MFGDQRVNAR HVSDVWRVGI HLEGLLGRDE IANGIGRLMG  425
AtUGT76C1   RWAPQLDVLA HRATGGFLTH NGWNSTLESI CEGVPMICLP CKWDQFVNAR FISEVWRVGI HLEGRIERRE IERAVIRLMV  414
                                         Consensus sequence

UGT85A122   GEKG------ KEMKRRAAGW RSAAEKAVAP GGSSYKNLEK LLSLLLPKK- ---------- --- 485
UGT85A123   GEKG------ KEMKRRAAEW KAAAEEAAAP GGSSHQNLEK LVALLLSEQ- ---------- --- 495
SbUGT85B1   GDLG------ KEKRAKAAEW KAAAEAAARK GGASWRNVER VVNDLLLVGG KQ-------- --- 492
UGT75AB1    EVVLGDGEKG RELRGNAKKW GELAKKAAKD GGSSDNNLRR FVD-----GL VKGTASSE-- --- 482
AtUGT75B1   EAVME--EKS VELRENAKKW KRLAMEAGRE GGSSDKNMEA FVEDICGESL IQNLCEAEEV KVK 469
UGT76AH1    DSSSS--KER EEMMKRVMDL KMKVDGCLKP GGSSYKSLQK LTCYLFSSCQ PEKL------ --- 477
AtUGT76C1   ESKG------ EEIRGRIKVL RDEVRRSVKQ GGSSYRSLDE LVDRISIIIE PLVPT----- --- 463
```

Supplemental Figure S19: Amino acid sequence alignment of candidate UGTs and characterized UGTs from Sorghum and Arabidopsis. The alignment was achieved with the ClustalW algorithm using BioEdit. Black shading corresponds to conserved residues, dark grey shades mark residues identical in six of the sequences, and residues with light grey shading are identical in five of the sequences. Conserved motifs are indicated (Gachon et al., 2005), PSPG: plant secondary product glycosyltransferase.

Substrate	Structure	UGT85A122	UGT85A123	UGT75AB1	UGT76AH1
PAOx	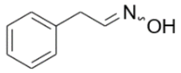	**yes**	**yes**	**yes**	no
salicylaldoxime	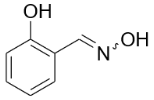	**yes**	**yes**	**yes**	no
benzaldoxime	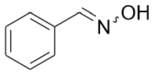	**yes**	**yes**	**yes**	no
β-mandelonitrile	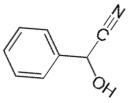	**yes**	**yes**	no	no
salicylic acid	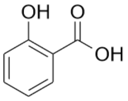	no	no	**yes**	no
4-OH-benzoic acid	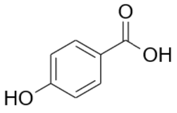	no	no	**yes**	no
gallic acid	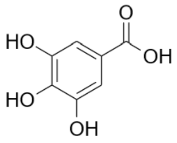	no	no	**yes**	no
quercetin	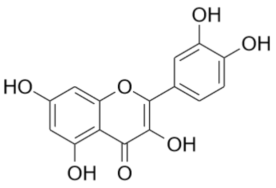	no	no	**yes**	**yes**
epicatechin	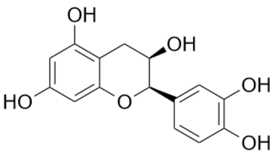	no	no	no	no
geraniol	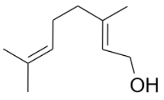	**yes**	**yes**	no	**yes**
oct-1-en-3-ol	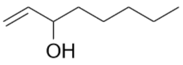	**yes**	**yes**	no	**yes**

Supplemental Figure S20: **Activity of UGT85A122, UGT85A123, UGT75AB1, and UGT76AH1 towards various substrates**. Candidate UGT genes were heterologously expressed in *Escherichia coli* and the purified enzymes used for activity assays with various substrates. Compounds were detected with LC-qTOF-MS.

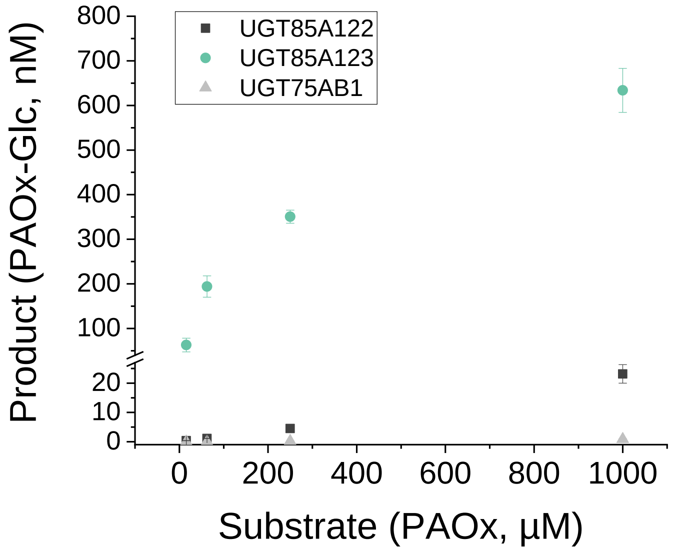

Supplemental Figure S21: **Substrate affinity of UGT85A122, UGT85A123, and UGT75AB1 towards PAOx**. Heterologous expression in *E. coli* and activity assays showed that three candidate UGTs were able to use PAOx as substrate. To compare their substrate affinities, 10 µg of the purified enzymes were incubated with various concentrations of PAOx for 30 min. Product formation was detected with targeted LC-MS/MS. Means ± SEM are shown (n = 3).

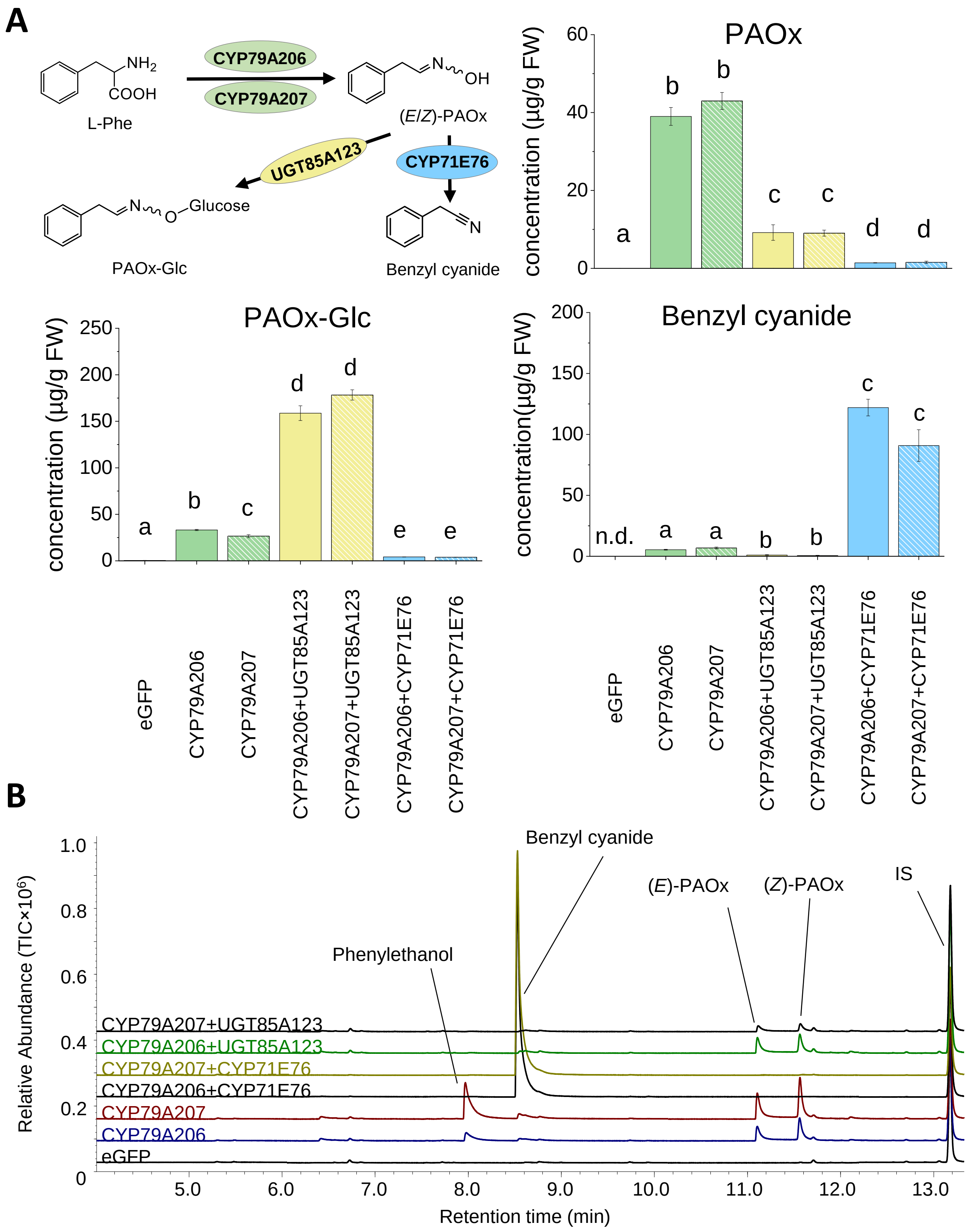

Supplemental Figure S22: Reconstitution of the formation of benzyl cyanide and phenylacetaldoxime glucoside in *Nicotiana benthamiana* by overexpression of tococa *CYP79, CYP71* and *UGT* genes. *N. benthamiana* was transformed with different combinations of recombinant *Agrobacterium tumefaciens* strains possessing CYP79A206, CYP79A207, CYP71E76, or UGT85A123, respectively. Accumulation of compounds was detected with targeted LC-MS/MS and GC-MS/FID. Means ± SEM are shown; n = 4-7. One-Way ANOVA (F_{PAOx} = 372.2, p<0.001; $F_{benzyl\ cyanide}$ = 32.34, p<0.001) or linear (gls) model (L-ratio$_{PAOx\text{-}Glc}$ = 144.0, p<0.001) and Tukey *post hoc* test on log transformed data, different letters indicate p<0.05; n.d., not detectable. **(A)** Overview of the pathway and quantification of the compounds of interest in the different plants. **(B)** Chromatogram from GC-MS analysis of the samples. FW, fresh weight. TIC: total ion chromatogram. IS: internal standard.

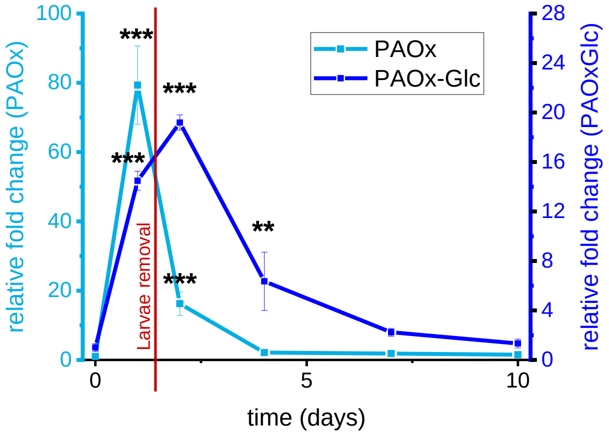

Supplemental Figure S23: **Relative temporal distribution of PAOx and PAOx-Glc in herbivore-damaged *Tococa quadrialata* leaves (see Figure 5)*.*** Leaves were exposed to herbivory by *Spodoptera littoralis* caterpillars for 24 hours and the accumulation of PAOx and PAOx-Glc was monitored for ten days. Compounds were extracted with methanol from leaf powder and analyzed using targeted LC-MS/MS. Here, we show the levels of PAOx and PAOxGlc relative to the respective concentration at the start of the experiment (t = 0d). Asterisks indicate significant differences to respective control (0d) based on One-Way ANOVA on log-transformed data ($F_{5,18,PAOx}$ = 41.03, $F_{5,18,PAOxGlc}$ = 12.96, $p_{PAOx,PAOxGlc} \leq 0.001$) and Dunnett's *post hoc* test. Means ± SEM are shown; n = 3-5.

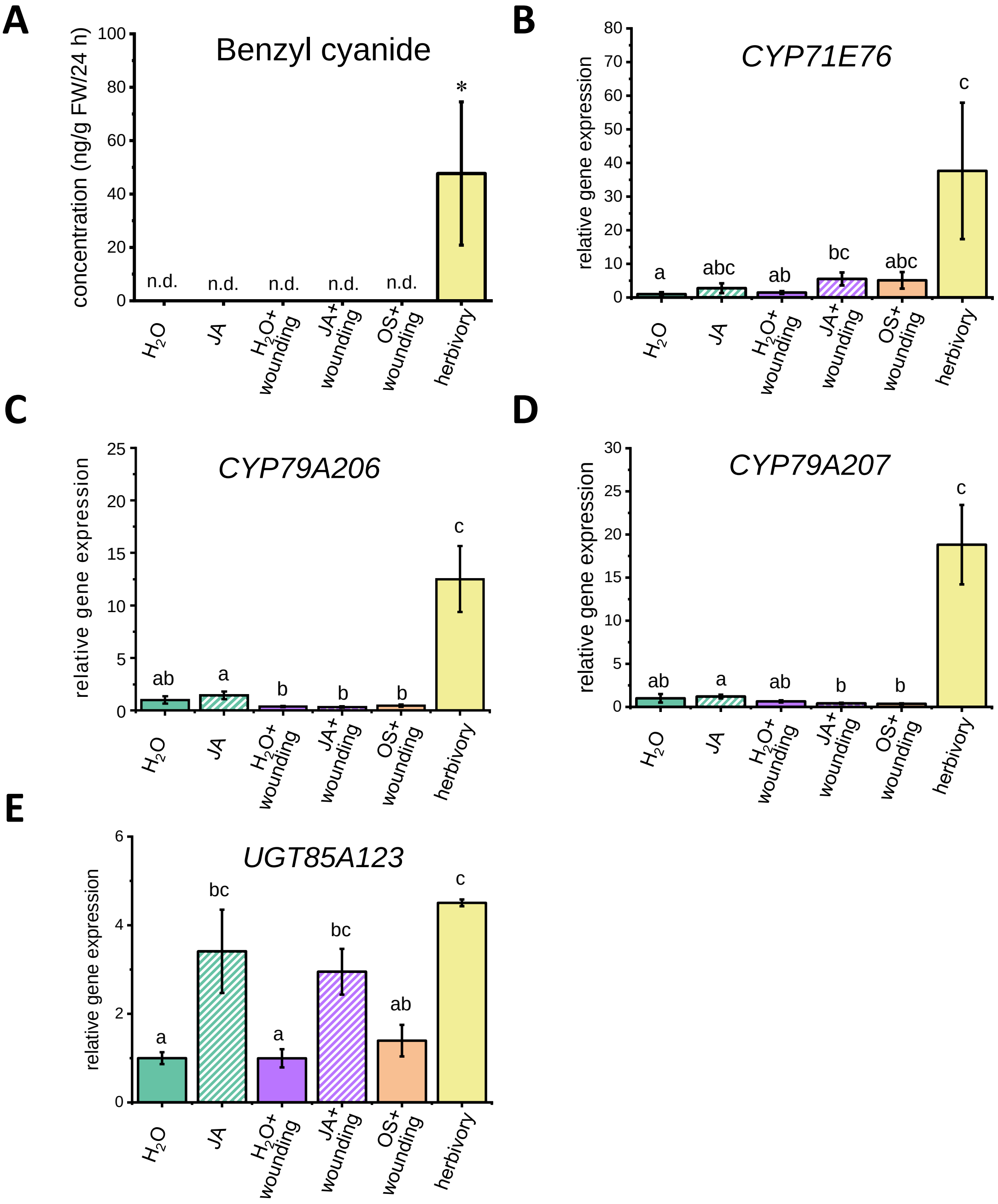

Supplemental Figure S24: **Gene expression of *CYP71E76*, *CYP79A206/207,* and *UGT85A123,* and formation of benzyl cyanide in tococa leaves in response to different treatments (see Figure 6).** Leaves were sprayed with jasmonic acid (JA), oral secretion (OS), or water (H_2O) after mechanical wounding or without further treatment. *S. littoralis* feeding served as a positive control. Volatiles were collected throughout the 24 h (after the) treatment, and quantified by GC-FID (**A)**. Means ± SEM are shown; n = 6-11. Asterisk indicates significant accumulation of benzyl cyanide (> 0) based on Wilcoxon signed rank test, * = p<0.05. All leaves were harvested 24 h after the (beginning of the) respective treatment, RNA extracted from leaf tissue, transcribed to cDNA, and RT-qPCRs performed (for primers see Supplemental Table S12) with actin as housekeeping gene (**B,C,D,E**). Relative expression was calculated with the ΔΔ Ct method (Pfaffl, 2001). Different letters indicate significant differences (p<0.05) between treatments based on One Way ANOVAs ($F_{CYP71E76}$ = 5.842, p = 0.001; $F_{UGT85A123}$ = 10.66, p <0.001) or GLS linear models (L-ratio$_{CYP79A206}$ = 33.36, L-ratio$_{CYP79A207}$ = 40.15, p <0.001) analyses on log transformed data and subsequent Tukey *post hoc* tests.

Methodology: cDNA synthesis from 900 ng RNA was performed with the SuperScript™ III First-Strand Synthesis Kit (Thermo Scientific) using oligo (dT)$_{12-18}$ primers according to the manufacturer's instructions. The obtained cDNA was diluted 1:10 with ddH_2O. Primer pairs were designed to amplify the respective gene (Supplemental Table S12), and their specificity confirmed by agarose gel electrophoresis, melting curve, and standard curve analysis. Samples were run in an optical 96-well plate on the CFX Connect Real-Time PCR System (Bio-Rad) with the Brilliant III Ultra-Fast SYBR Green QPCR Master Mix (Agilent). PCR conditions are given in Supplemental Table S11. All samples were run in triplicates. Normalized fold expression was calculated with the ΔΔCP method (Pfaffl, 2001).

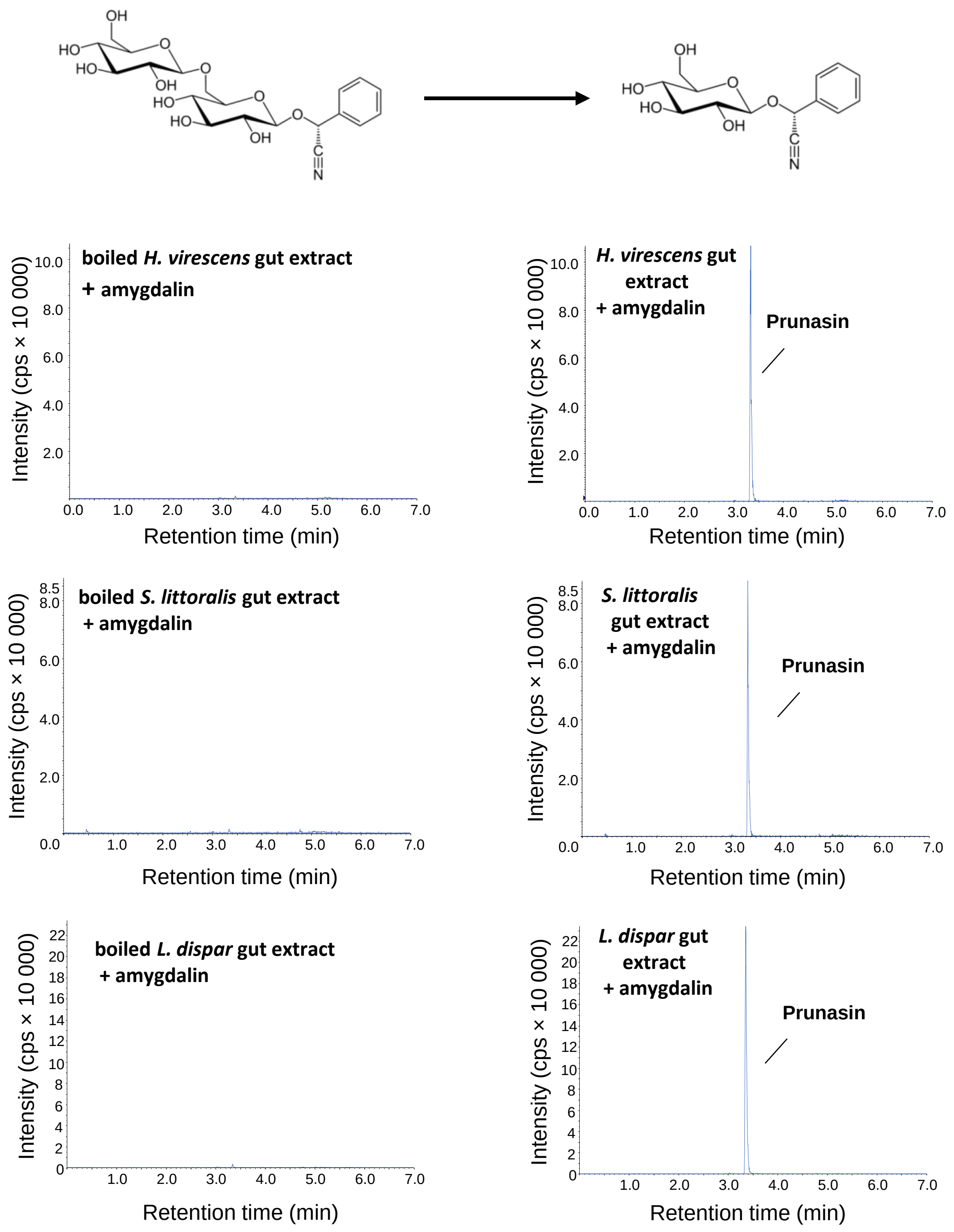

Supplemental Figure S25: **Deglycosylation of amygdalin by gut enzymes of different caterpillars.** Gut extracts of *Heliothis virescens*, *Spodoptera littoralis,* and *Lymantria dispar* were tested for their ability to form prunasin from amygdalin. Targeted LC-MS/MS analysis revealed glucosidase activities for all tested gut extracts. Boiled extracts were used as negative controls. Cps: counts per second.

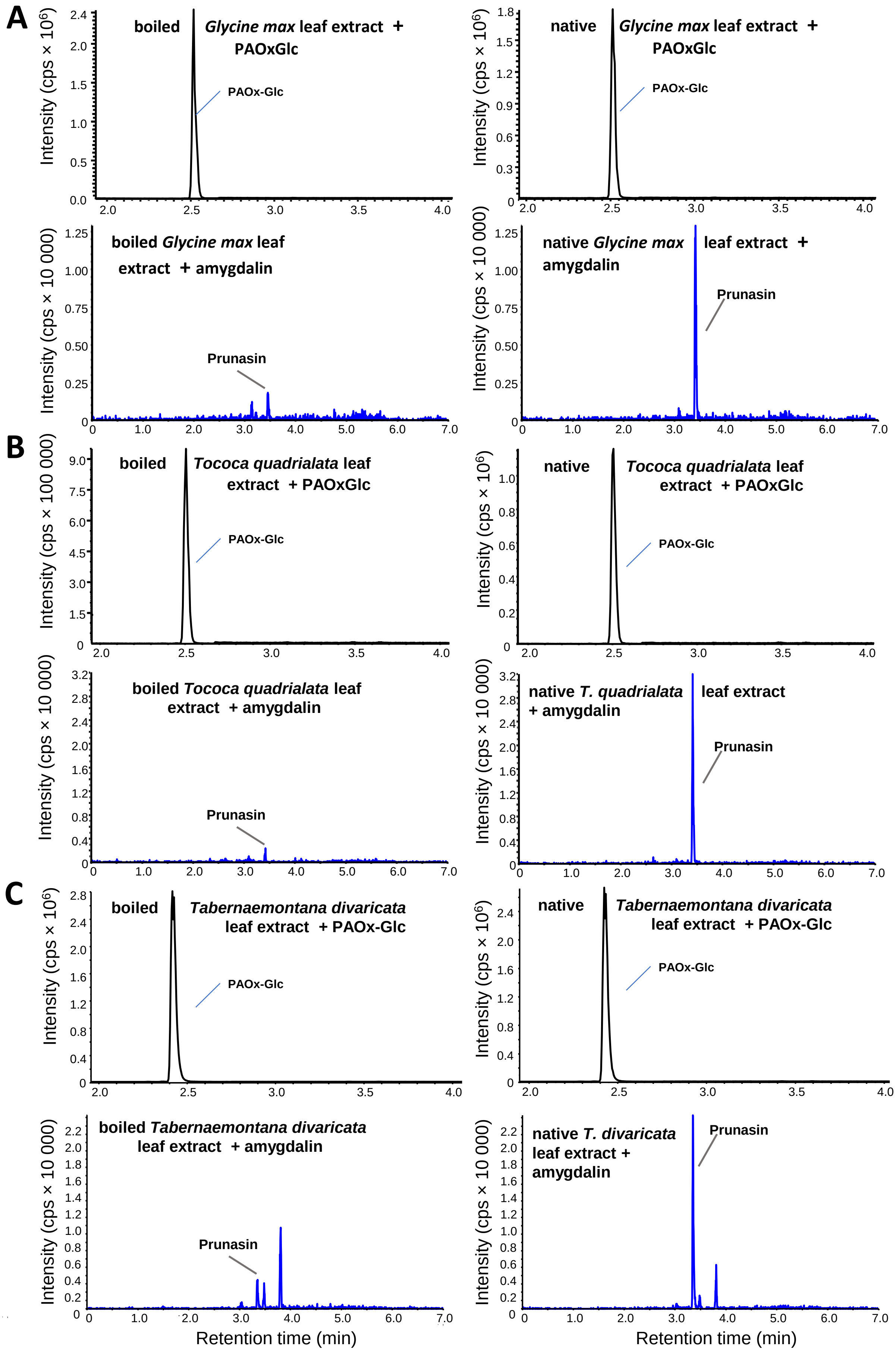

Supplemental Figure S26: **Deglycosylation of amygdalin but not PAOx-Glc by crude enzyme extracts of various plant species.** Plant extracts of herbivore-damaged leaves of *Glycine max* (**A**), *Tococa quadrialata* (**B**), and *Tabernaemontana divaricata* (**C**) were tested for their ability to form prunasin from amygdalin and PAOx from PAOx-Glc. Targeted LC-MS/MS analysis revealed glucosidase activities towards amygdalin for these extracts. Boiled extracts served as negative controls. Cps: counts per second.

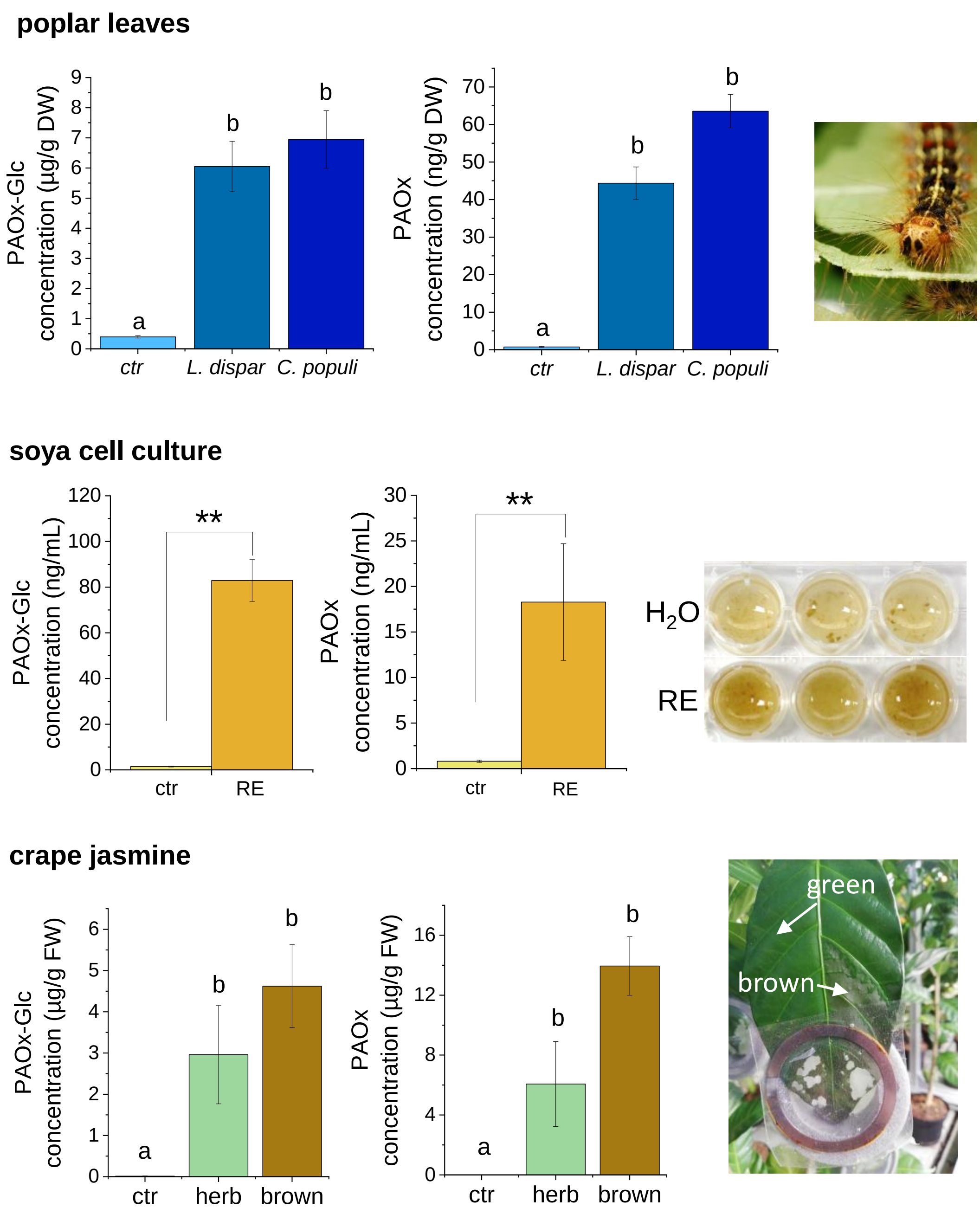

Supplemental Figure S27: **Identification of PAOx-Glc in different plant species upon biotic stress.** Blue: PAOx-Glc accumulation in Western balsam poplar (*Populus trichocarpa*) leaves upon *Lymantria dispar and Chrysomela populi* feeding. Leaves were extracted with methanol and PAOx(-Glc) detected using LC-MS/MS. One-Way ANOVA (F_{PAOx} = 411.2, p<0.001; $F_{PAOx\text{-}Glc}$ = 125.1, p<0.001) with Tukey *post hoc* on log transformed data, different letters indicate significant differences (p<0.05); Means ± SEM are shown; n = 8; DW, dry weight. Yellow: soybean cell cultures release PAOx and PAOx-Glc upon induction with raw elicitor (RE) from the oomycete *Phytophthora sojae*. The supernatant of the cell cultures was analyzed using LC-MS/MS. Student's t-test; **: p<0.01; Means ± SEM are shown; n = 6. Green, brown*: Spodoptera littoralis*-wounded leaves of crape jasmine (*Tabernaemontana divaricata*) produce PAOx and PAOx-Glc. Leaves were extracted with methanol and PAOx(-Glc) detected using LC-MS/MS. One-Way-ANOVA (F_{PAOx} = 58.06, p<0.001; $F_{PAOx\text{-}Glc}$ = 34.38, p<0.001) with Tukey *post hoc* on log transformed data, different letters indicate significant differences (p<0.05); Means ± SEM are shown; n = 3; FW, fresh weight.

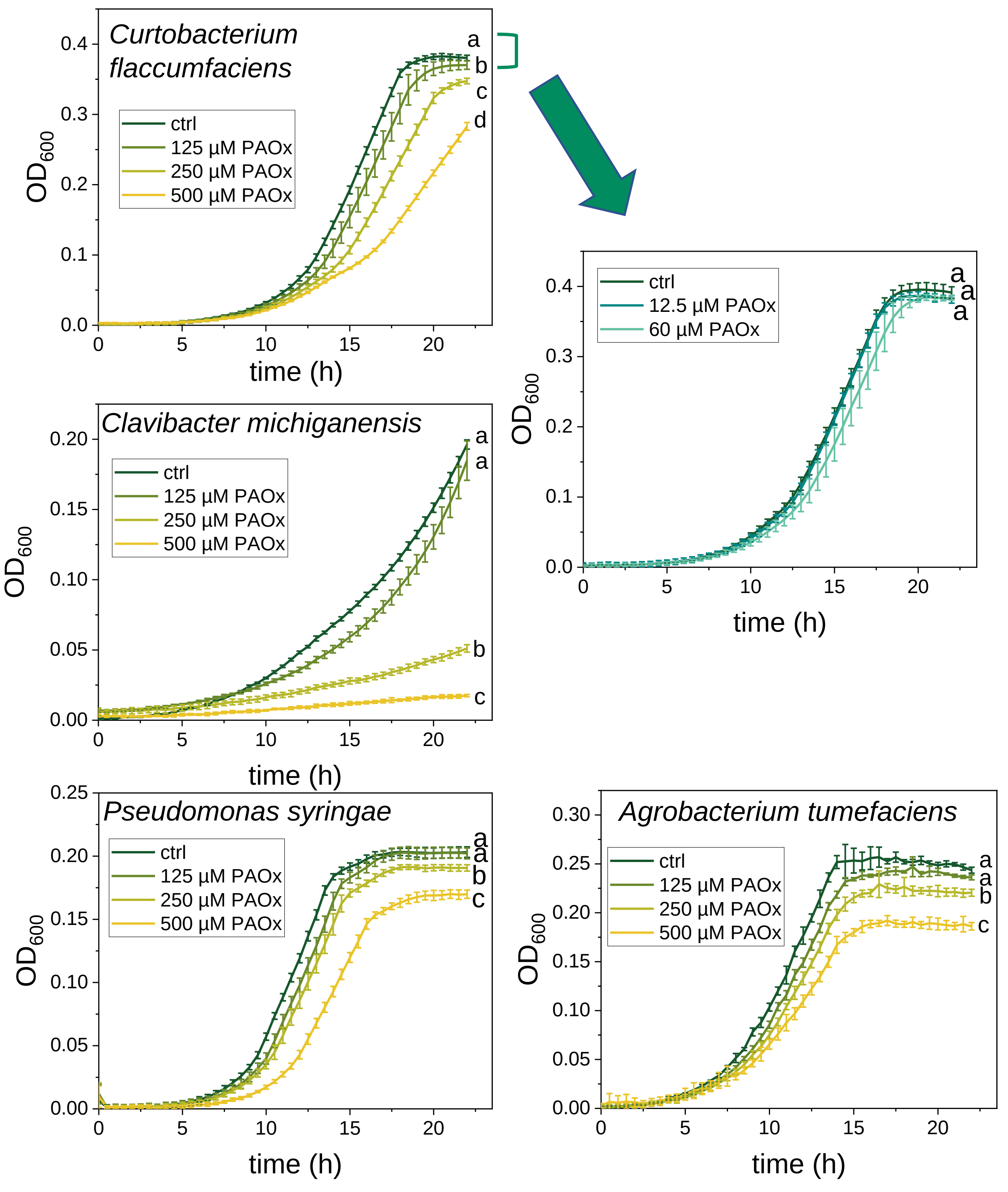

Supplemental Figure S28: **Negative effects of PAOx on the growth of several plant pathogenic bacteria.** The effect of PAOx on gram-negative (*Agrobacterium tumefaciens*, *Pseudomonas syringae*) and gram-positive (*Curtobacterium flaccumfaciens*, *Clavibacter michiganensis*) bacteria strains was tested in a bacterial growth assay in microtiter plates. Growth of the cultures was monitored over 22 h by measuring the OD_{600} every 30 min. Data are shown as means ± SD; n = 3-6. Different letters indicate significant differences (p<0.05) between treatments after 22 h. *C. michiganensis*: log transformed; one-way ANOVA ($F_{C.michiganenis}$ = 1771, p <0.001; $F_{A.tumefaciens}$ = 184.1, p <0.001, $F_{P.syringae}$ = 108.8, p <0.001) with Tukey HSD posthoc test or Kruskal-Wallis test ($\chi^2_{C.flaccumfaciens\ (highconc)}$ = 20.98, p <0.001; $\chi^2_{C.flaccumfaciens\ (lowconc)}$ = 3.317, p = 0.19) followed by multiple pairwise comparisons using Wilcoxon rank sum tests with a Holm correction.

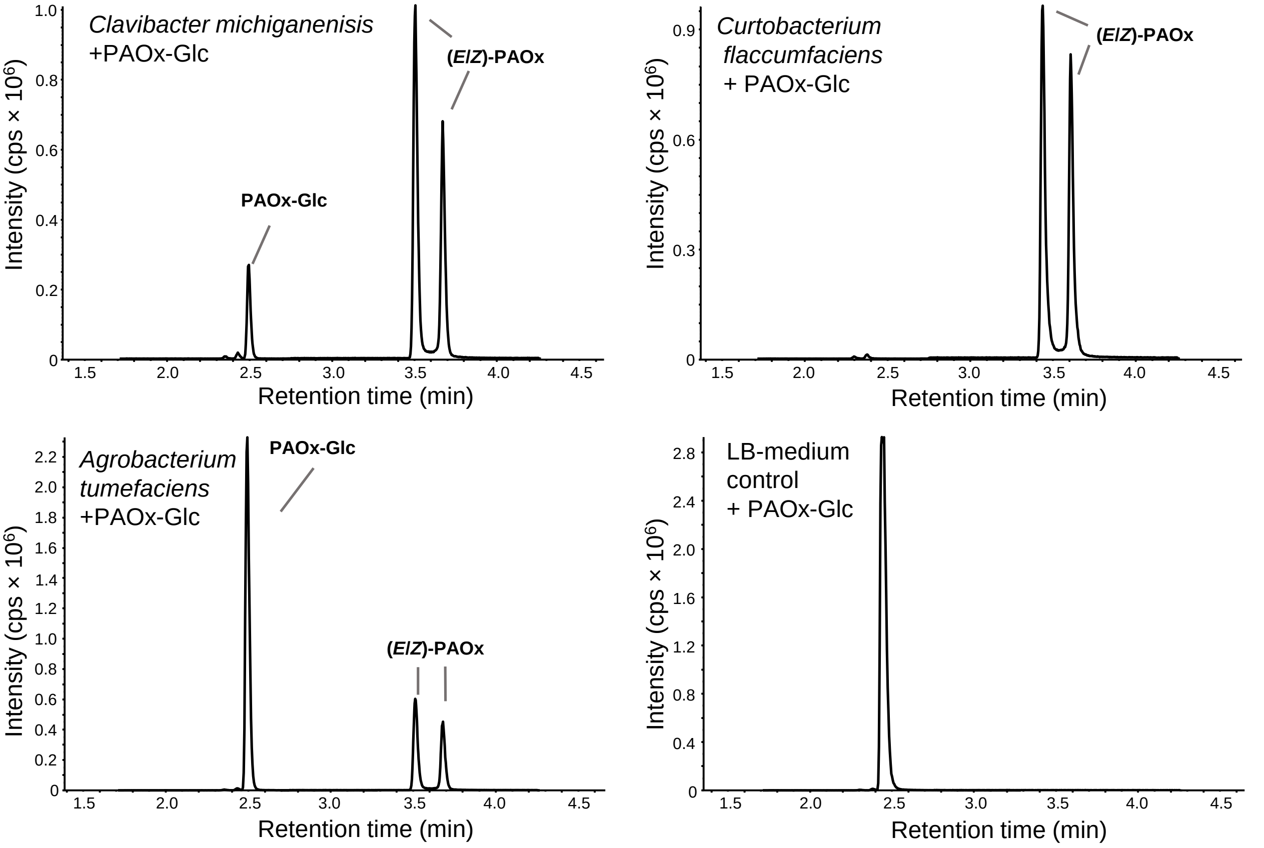

Supplemental Figure S29: **Several plant pathogenic bacteria are able to deglycosylate PAOx-Glc*.*** Gram-negative (*Agrobacterium tumefaciens*) and gram-positive (*Curtobacterium flaccumfaciens*, *Clavibacter michiganensis*) bacteria strains were tested for their ability to deglycosylate PAOx-Glc. Bacteria were grown in the presence of PAOx-Glc for 24 h in a microtiter plate, and the LB-medium was analyzed afterwards using liquid chromatography-tandem mass spectrometry (LC-MS/MS).

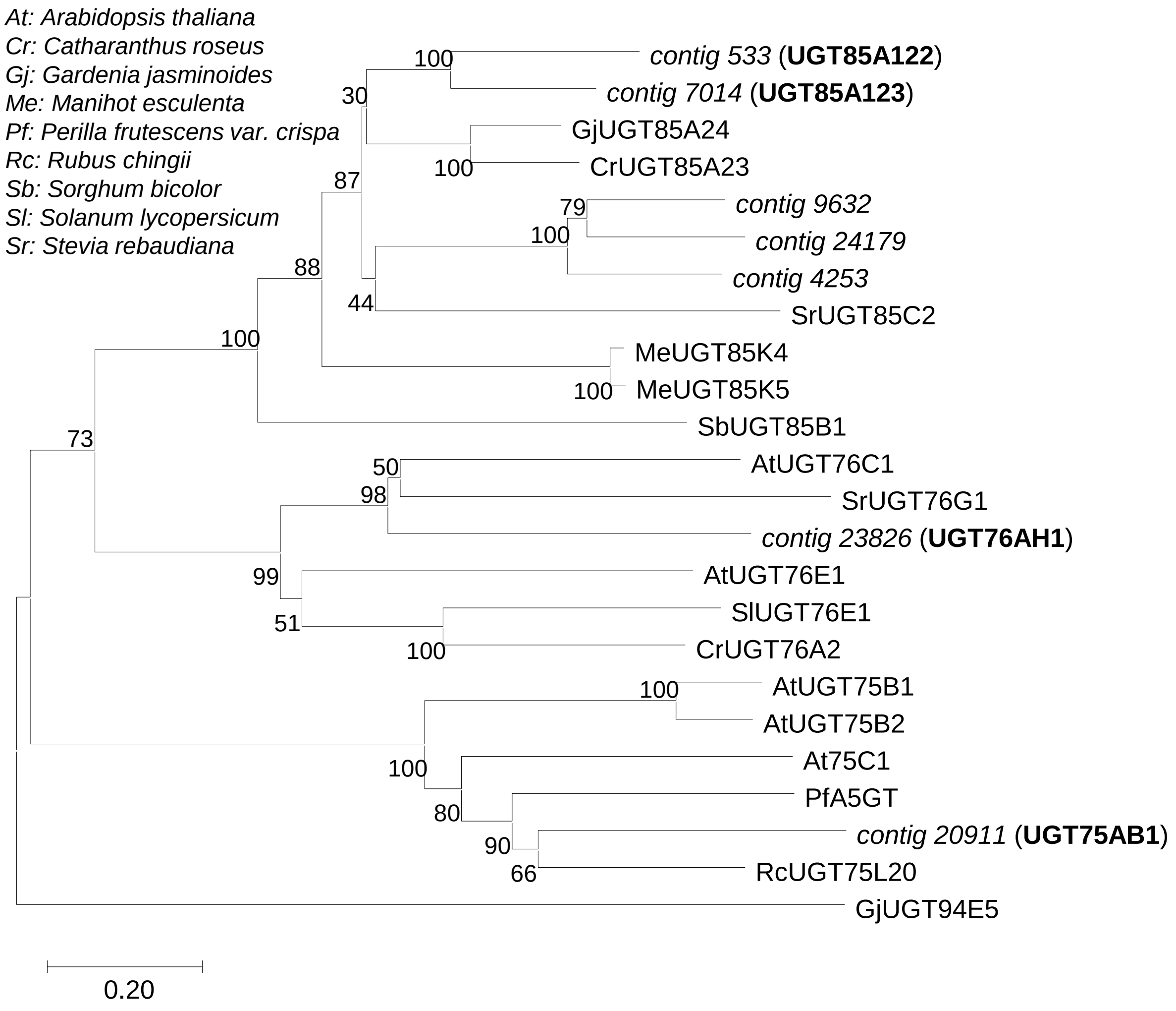

Supplemental Figure S30: Phylogenetic tree of candidate UGTs from tococa. The phylogenetic relationship of 7 putative *Tococa quadrialata* UGTs with already characterized UGTs of the respective subfamilies from other plants is shown. The tree was inferred with the Neighbor-joining method and n = 1000 replicates for bootstrapping. Bootstrap values are shown next to each node. The scale indicates in the number of substitutions per site. Putative UGTs from tococa are shown in italics, the characterized ones in bold. GjUGT94E5 (BAK55744.1) was used as an outgroup. UGT: family 1 UDP-glycosyltransferase.

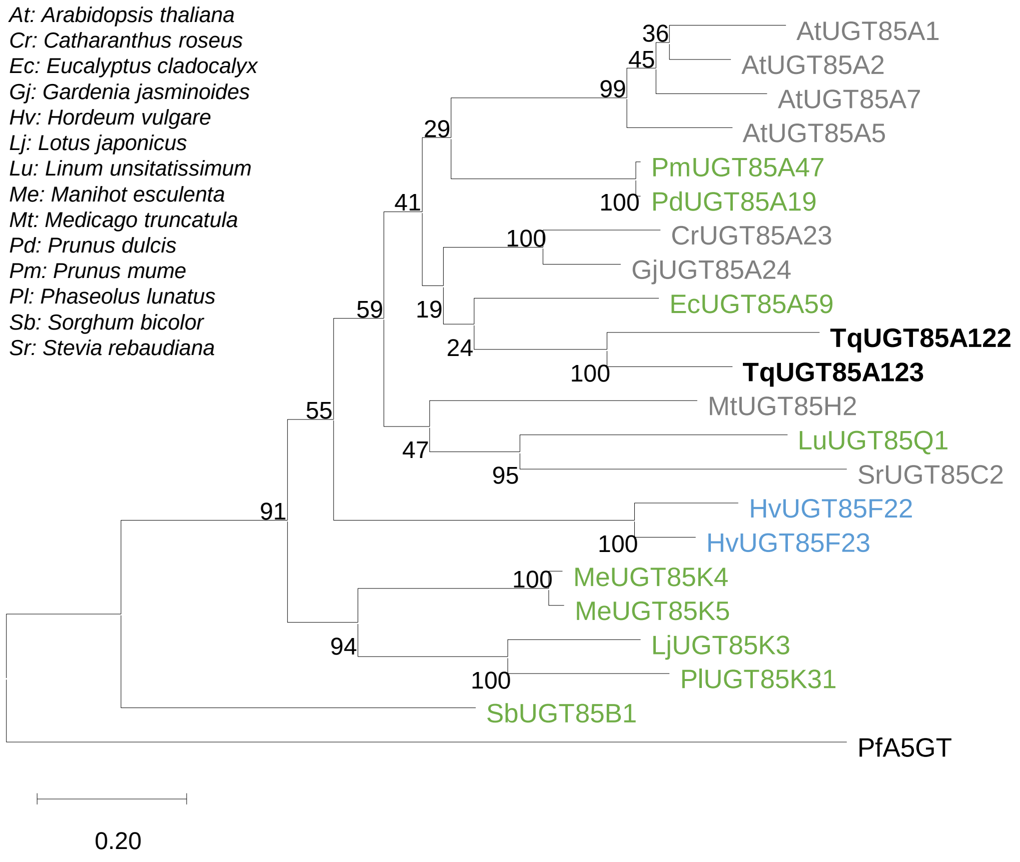

Supplemental Figure S31: Phylogenetic tree of characterized UGTs of the subfamily 85. The phylogenetic relationship of UGT85s involved in the biosynthesis of hydroxynitrile-glucosides with UGT85 using other substrates is shown. The tree was inferred with the Maximum-Likelihood method and n = 1000 replicates for bootstrapping. Bootstrap values are shown next to each node. The scale indicates in the number of substitutions per site. The characterized UGT85s from tococa are shown in bold, UGTs involved in the biosynthesis of cyanogenic glucosides in green, of non-cyanogenic hydroxynitrile-glucosides in blue, and those using different compound classes as substrates in grey. PfA5GT (Q9ZR27) was used as an outgroup. UGT: family 1 UDP-glycosyltransferase.

Supplemental Table S1: UGT candidate genes for the glycosylation of PAOx-Glc.

Feature ID	P-value	Fold change	ctr - Mean RPKM	herb - Mean RPKM	UGT sub-family	Length	SWISSPROT: Description	NCBI: Description
**contig_533**	0.000	7	32	221	UGT85	1928	UGT2_GARJA ame: Full=7-deoxyloganetin glucosyltransferase ame: Full=Genipin glucosyltransferase ame: Full=UDP-glucose glucosyltransferase 2 Short= 2 ame: Full=UDP-glycosyltransferase 85A24	7-deoxyloganetin glucosyltransferase-like
**contig_7014**	0.000	4	15	67	UGT85	2692	UGT2_GARJA ame: Full=7-deoxyloganetin glucosyltransferase ame: Full=Genipin glucosyltransferase ame: Full=UDP-glucose glucosyltransferase 2 Short= 2 ame: Full=UDP-glycosyltransferase 85A24	7-deoxyloganetin glucosyltransferase-like isoform X1
contig_4253	0.002	2	16	38	UGT85	1657	UGT2_GARJA ame: Full=7-deoxyloganetin glucosyltransferase ame: Full=Genipin glucosyltransferase ame: Full=UDP-glucose glucosyltransferase 2 Short= 2 ame: Full=UDP-glycosyltransferase 85A24	7-deoxyloganetin glucosyltransferase
contig_24179	0.001	6	3	16	UGT85	1176	U85A3_ARATH ame: Full=UDP-glycosyltransferase 85A3	7-deoxyloganetin glucosyltransferase
contig_9632	0.022	5	1	5	UGT85	1630	UGT2_GARJA ame: Full=7-deoxyloganetin glucosyltransferase ame: Full=Genipin glucosyltransferase ame: Full=UDP-glucose glucosyltransferase 2 Short= 2 ame: Full=UDP-glycosyltransferase 85A24	7-deoxyloganetin glucosyltransferase
**contig_23826**	0.001	15	0	6	UGT76	1597	U76B1_ARATH ame: Full=UDP-glycosyltransferase 76B1	UDP-glycosyltransferase 76C2
contig_329	0.012	2	7	15	UGT76	2943	UGT7_CATRO ame: Full=UDP-glucose iridoid glucosyltransferase ame: Full=UDP-glucose glucosyltransferase 7 Short= 7 ame: Full=UDP-glycosyltransferase 76A2	UDP-glucose iridoid glucosyltransferase
**contig_20911**	0.007	4	67	237	UGT75	1978	UGT1_GARJA ame: Full=Crocetin chloroplastic ame: Full=UDP-glucose glucosyltransferase 1 Short= 1 ame: Full=UDP-glycosyltransferase 75L6 Flags: Precursor	crocetin chloroplastic
contig_11576	0.045	3	1	5	UGT75	1121	UGT1_GARJA ame: Full=Crocetin chloroplastic ame: Full=UDP-glucose glucosyltransferase 1 Short= 1 ame: Full=UDP-glycosyltransferase 75L6 Flags: Precursor	crocetin chloroplastic
contig_5159	0.000	3	17	54	UGT73	1855	SCGT_TOBAC ame: Full=Scopoletin glucosyltransferase ame: Full=Phenylpropanoid:glucosyltransferase 1	scopoletin glucosyltransferase
contig_5158	0.000	5	8	44	UGT73	1042	SCGT_TOBAC ame: Full=Scopoletin glucosyltransferase ame: Full=Phenylpropanoid:glucosyltransferase 1	scopoletin glucosyltransferase
contig_9912	0.045	2	3	8	UGT73	1858	SCGT_TOBAC ame: Full=Scopoletin glucosyltransferase ame: Full=Phenylpropanoid:glucosyltransferase 1	scopoletin glucosyltransferase
contig_9005	0.021	3	3	7	UGT83	1627	U83A1_ARATH ame: Full=UDP-glycosyltransferase 83A1	UDP-glycosyltransferase 83A1
contig_8815	0.003	2	21	47	UGT89	1638	U89A2_ARATH ame: Full=UDP-glycosyltransferase 89A2	UDP-glycosyltransferase 89A2-like

All contigs of the *de novo* assembly encoding glycosyltransferase family 1 type glycosyltransferases with a minimum sequence length of 1000 bp that are significantly upregulated upon herbivore damage ($p<0.05$, fold change ≥ 2) are listed. Sequences chosen for further characterization are in bold. Selection was based on UGT subfamily, RPKM values and fold change in herbivore-damaged samples.

Supplemental Table S2: Growth conditions for *Tococa quadrialata.*

**Greenhouse conditions**	
Temperature<br>Day:<br>Night:	 23-25 °C<br>16-18 °C
Humidity	70%
Light/dark cycle	16 h/8 h
**composition of the soil**	
Percentage	*Material*
25%	Klasmann potting substrate (Klasman-Deilmann, Geeste, Germany)
25%	Latvian white peat
25%	pine bark (7-15 mm)
12.5%	Legaton (5-7 mm)
12.5%	sand
Inoculated with	BioMyc™ Vital Mykorrhiza **(**BioMyc, Brandenburg, Germany).

Supplemental Table S3: Chromatographic gradients for the analysis of jasmonates, aldoximes, and prunasin using an LC-TripleQuad-MS system.

**Gradient**	**HPLC**	**Solvents** (A/B)	**Flow** (µl/min)	**Time** (min)	**Temperature** (°C)	**Solvent A** (%)	**Solvent B** (%)
A1	Agilent 1200	0.2% FA in ddH_2O (A), Acetonitrile (B)	1100	0<br>4<br>4.1<br>5<br>5.1<br>8	25 °C	90<br>30<br>0<br>0<br>90<br>90	10<br>70<br>100<br>100<br>10<br>10
A2	Agilent 1260	0.05% FA in ddH_2O, Acetonitrile (B)	1100	0<br>4<br>4.1<br>5<br>5.1<br>8	20 °C	90<br>30<br>0<br>0<br>90<br>90	10<br>70<br>100<br>100<br>10<br>10
B	Agilent 1260	0.05% FA in ddH_2O, Acetonitrile (B)	1100	0<br>0.5<br>4<br>4.1<br>4.5<br>4.6<br>7	20 °C	95<br>95<br>50<br>0<br>0<br>95<br>95	5<br>5<br>50<br>100<br>100<br>5<br>5
C	Agilent 1260	0.05% FA in ddH_2O, Acetonitrile (B)	1100	0<br>0.5<br>6<br>6.02<br>7.5<br>9.5<br>9.52<br>12	20 °C	95<br>95<br>62.6<br>80<br>0<br>0<br>95<br>95	5<br>5<br>37.4<br>80<br>100<br>100<br>5<br>5

All separations were achieved on a Zorbax Eclipse XDB C18 column (50 mm 4.6 mm, 1.8 µm, Agilent Technologies). For MS parameters, see Supplemental Table S4.

Supplemental Table S4: MRM parameters for the LC-MS/MS analysis of jasmonates, aldoximes, and prunasin using an LC-TripleQuad-MS system.

compound	Mass spectrometer	mode	HPLC gradient	Q1	Q3	DP (V)	CE (V)	Retention time (min)
(*E*/*Z*)-phenyl-acetaldoxime	API5000	pos	A1	136.1	119.1	56	17	3.5; 3.7
	API6500	pos	A2	136.1	119.1	30	17	3.4; 3.6
Phenylacetald-oxime glucoside	API5000	pos	A1	298.0	136.0	70	13	2.5
	API6500	pos	A2	298.0	136.0	30	13	2.5
Prunasin	API6500	neg	B	340.0	59.0	-40	-56	3.4
JA	API6500	neg	C	209.1	59.0	-30	-24	7.2
D_6-JA	API6500	neg	C	215.1	59.0	-30	-24	7.2
D_5-JA	API6500	neg	C	214.1	59.0	-30	-24	7.2
JA-Ile	API6500	neg	C	322.2	130.1	-30	-30	7.3
D_6-JA-Ile	API6500	neg	C	328.2	130.1	-30	-30	7.3
D_5-JA-Ile	API6500	neg	C	327.2	130.1	-30	-30	7.3

All separations were achieved according to Supplemental Table S3.

Supplemental Table S5: Non-target metabolite analysis by LC-ESI-Q-ToF-MS.

**Chromatographic gradients for untargeted metabolite analysis using ultra-high-performance liquid chromatography–electrospray ionization– high resolution mass spectrometry (UHPLC–ESI–HRMS)**							
**HPLC**	**column**	**Solvents** (A/B)	**Flow** (µl/min)	**Time** (min)	**Temperature** (°C)	**Solvent A** (%)	**Solvent B** (%)
Dionex Ultimate 3000 series UHPLC (Thermo Scientific)	Zorbax Eclipse XDB-C18 column (100 mm × 2.1 mm, 1.8 µm, Agilent)	0.1% FA in ddH_2O (A), Acetonitrile (B)	300	0<br>0.5<br>11<br>11.1<br>12<br>12.1<br>15	25 °C	95<br>95<br>40<br>0<br>0<br>95<br>95	5<br>5<br>60<br>100<br>100<br>5<br>5
**Parameters of the Bruker timsToF mass spectrometer** (Bruker Daltonics, Bremen, Germany)							
**Mode**	**Capillary voltage (**kV)	**End plate offset** (V)	**Nebulizer pressure** (bar)	**Drying gas**	**Acquisition** (Hz)	**Mass range** (*m*/*z*)	**Data processing software**
positive	4.5	500	2.8	Nitrogen (8L/min, 280°C)	12	50-1500	MetaboScape (Bruker Daltonics)
negative	-3.5	500	2.8	Nitrogen (8L/min, 280°C)	12	50-1500	MetaboScape software (Bruker Daltonics)

At the beginning of each chromatographic analysis 10µL of a sodium formate-isopropanol solution (10 mM solution of NaOH in 50/50 (*v*/*v*) isopropanol- water containing 0.2% formic acid) was injected into the dead volume of the sample injection for recalibration of the mass spectrometer using the expected cluster ion *m*/*z* values.

Supplemental Table S6: Temperature profile of benzyl cyanide measurements by GC-FID and GC-MS.

	hexane extracts	
**Initial temperature**	60°C	2 min
**Ramp I**	60°C<br>↓<br>160°C	6°C/min
**Ramp II**	160°C<br>↓<br>300°C	60°C/min
**Final temperature**	300 °C	2 min

Supplemental Table S7: Parameters used for the *de novo* assembly.

**Assembly parameters**	
Word size	35
Bubble size	650
**Quality of the *de novo* assembly**	
total contigs	49,957
N50	1,659
Complete BUSCOs*	78.5%
Missing BUSCOs*	10.2%
**Annotation**	
BLAST2GO#	Swiss-Prot, NCBI

*Benchmarking Universal Single-Copy Orthologs (BUSCO) analysis (https://busco.ezlab.org/, last accessed on 04.02.2022; (Simao et al., 2015))

#(Gotz et al., 2008)

Supplemental Table S8: Primers used for cloning.

Name	Sequence	Usage
CYP79A206_subc_fwd	ATGAATATTTCTGCTTCCGCC	cloning, in pJET1.2/blunt
CYP79A206_subc_rev	CTAGAATGACGGGTAAAGGTG	cloning, in pJET1.2/blunt
CYP79A207_subc_fwd	ATGAATATTTCCGCTTACGC	cloning, in pJET1.2/blunt
CYP79A207_subc_rev	CTAGAATGATGGGTAAAGGTG	cloning, in pJET1.2/blunt
CYP71E76_subc_fwd	ATGTCTTCTACAATTATTTCCTTC	cloning, in pJET1.2/blunt
CYP71E76_subc_rev	TTAATTATATTTAGTTGGAACCAAACG	cloning, in pJET1.2/blunt
UGT85A123_subc_fwd	ATGAGTTCTGAACAAGAACAGAGC	cloning, in pJET1.2/blunt
UGT85A123_subc_rev	TCATTGCTCGGAAAGAAGCAGTG	cloning, in pJET1.2/blunt
CYP79A206_ *Not*I_fwd	**CAA**GCGGCCGCAATGAATATTTCTGCTTCCG	cloning, in pESC-Leu2d
CYP79A206_*Pac*I_rev	**GTC**TTAATTAACTAGAATGACGGGTAAAGGTG	cloning, in pESC-Leu2d
CYP79A207_ *Spe*I_fwd	**AAG**ACTAGTAATGAATATTTCCGCTTACGC	cloning, in pESC-Leu2d
CYP79A207_*Sac*I_rev	**ACA**GAGCTCCTAGAATGATGGGTAAAGGTG	cloning, in pESC-Leu2d
CYP71E76_*Spe*I_fwd	**TAA**ACTAGTAATGTCTTCTACAATTATTTCCTTC	cloning, in pESC-Leu2d
CYP71E76_*Sac*I_rev	**ACG**GAGCTCTTAATTATATTTAGTTGGAACCAAAC	cloning, in pESC-Leu2d
CYP79A206_USER_fwd	**GGCTTAAU**ATGAATATTTCTGCTTCCGCCG	cloning, in pCAMBIA2300U
CYP79A206_USER_rev	**GGTTTAAU**CTAGAATGACGGGTAAAGGTGC	cloning, in pCAMBIA2300U
CYP79A207_USER_fwd	**GGCTTAAU**ATGAATATTTCCGCTTACGCCG	cloning, in pCAMBIA2300U
CYP79A207_USER_rev	**GGTTTAAU**CTAGAATGATGGGTAAAGGTGC	cloning, in pCAMBIA2300U
CYP71E76_USER_fwd	**GGCTTAAU**ATGTCTTCTACAATTATTTCCTTCCC	cloning, in pCAMBIA2300U
CYP71E76_USER_rev	**GGTTTAAU**TTAATTATATTTAGTTGGAACCAAACGC	cloning, in pCAMBIA2300U
UGT85A123_USER_fwd	**GGCTTAAU**ATGAGTTCTGAACAAGAACAGAGC	cloning, in pCAMBIA2300U
UGT85A123_USER_rev	**GGTTTAAU**TCATTGCTCGGAAAGAAGCAGTG	cloning, in pCAMBIA2300U
UGT76AH1_pET100_fwd	CACCATGGGCACAACCTCAAGTCAAG	cloning, in pET100
UGT76AH1_pET100_rev	TTATAATTTTTCCGGTTGGCATGAG	cloning, in pET100

Primer name indicates the gene to be amplified. The important sequence motif for cloning in the respective vector is underlined or in bold

Supplemental Table S9: Codon-optimized ORF of tococa UGTs for expression in *Escherichia coli*.

Encoded enzyme	Codon optimized nucleotide sequence
UGT85A122	ATGGCACGTGATAGCGCAGTTGAAGCACTGCCTGGCACCAAACCGCATGCAATTATTGTTCCGTATCCG<br>GCACAGGGTCATATTAATCCGCTGCTGAAACTGGCAAAAATTCTGCATGGTCGTGGTGGTTTTCATGTGA<br>CCTTTGTTAATACCGAATATAACGCACGTCGTCTGCTGCGTAGCCGTGGTCCGACCAGCCTGGATGATAT<br>GCCAGGTTTTCATTTTGAAACCATTCCGGATGGTCTGCCTCCGGTTGATGCAGATGTTATGCAGGATGTT<br>CCGGCACTGAGCGATAGCCTGAGCCGTACCTGTTTTGAACCGTTTGTTGAACTGGTTAGCAAAGTGAATC<br>AGCATGGTGGTCCGCCTGCAACCTGCTTTGTTTATGATGCAATGATGCCGTTTGTGGCAGATGCAGCAG<br>AAAAATTTGGTCTGCCAGCAGTTGCATTTTGGCCTCCGGCAGCATGTGCAATTTGGGGTGTTGCACAGTA<br>TCCGAAACTGATTGAAAAAGGTCTGGTTCCGCTGAAAGATAGCAGTTATCTGAGCAATGGTTTTCTGGAT<br>ACCGTGATTGAATGGATTCCGGGTATTGATAATTTTCGCCTGCGTGATATTCCGACCTTTATTCAGACCAC<br>CGATCCGAATGATATTATGGTGCATTACATGATTCGTAGCGTTGAAGTGAGCAGCAAAAAAGCATGTGCC<br>GTTGTGTTTAATACCTTTGATGCACTGGAAGCAGATGTTCTGCGTGCACTGAAAAGCATTTATCCGAGCAT<br>TTATACCGTTGGTCCGCTGAATCTGGTTCTGGATCGTTATCCGGAAGAAGAACTGACCGCAGTTGGTAGC<br>AATCTGTGGAAAGAAGATAATAGCTGTCTGAAATGGCTGGATAGCCAAGAACATGGTAGCGTTGTTTATG<br>TGAATTTTGGTAGCATTACCGTTGCAACCGCAGAGCAGATGACCGAATTTGCATGGGGTTTAGCAAATAG<br>CCGTATGCCGTTTCTGTGGGTTATTCGTCCGGATCTGGTTGTTGGTGAAAGCGCAGTGCTGCCACCGGG<br>TTTTACCGATGAAACCAGCGATCGTTGTATGATTAGCGGTTGGTGTCCGCAAGAAGAAGTTCTGAAACAT<br>CCGAGTATTGGTGGCTTTCTGACCCATAGTGGTTGGAATAGCACCCTGGAAAGCATGAGTGCCGGTGTT<br>CCGATGGTTTGTTGGCCGTTTTTTGCCGATCAGCAGACCAATTGTTGGTATGGTAAAAATAGCTGGGGTA<br>TCGGCATGGAAATTGATCATGATGTGAAACGCGATAAAGTGGAAGGTATGGTTAAAGAACTGATGGAAGG<br>TGAAAAGGGCAAAGAAATGAAACGTCGTGCAGCAGGTTGGCGTAGCGCAGCCGAAAAAGCAGTTGCCC<br>CTGGTGGTAGCAGCTATAAAAACCTGGAAAAACTGCTGAGCCTGCTGCTGCCGAAAAAATGA
UGT85A123	ATGAGCAGCGAACAAGAACAGAGCATGAAAACCCATGTTCATCCGAATCCGAAACCGCATGCAGTTTTTA<br>TTCCGTATCCGGCACAGGGTCATATTAATCCGATTCTGAAACTGGCAAAACTGCTGCATCGTTGTCATGG<br>CTTTCATGTTACCTTTGTGCATACCGAATATAACCATCGTCGTCTGCTGCGTAGCCGTGGTCCGGATAGC<br>CTGCTGGGTCTGCCTGGTTTTCGTTTTGAAACCATTCCGGATGGTCTGCCTCCGACACCGGAAGATGCA<br>AGTGATGATGTTACCCAGGATATTCCGAGCCTGTGTGATAGCACCAGCCGTACCTGTACCGCACCGTTTA<br>TTAGCCTGGTGCGTCGTCTGAATGCCGAAGAAGGTGGTCCGCCTGTTAGCTGTGTTGTTTTTGATGGTG<br>CAATGAGCTTTGCACTGGATGCAGCCGAAGAATTTGGTGTTCCGGGTGTTGCATATTGGACCCCGAGCG<br>CATGTGGTGTTCTGGCATATAGCTATTATCATAAGCTGGTCGAAAAAGGTCTGAGTCCGCTGAAAGATAG<br>CAGTTATCTGACCAATGGTTATCTGGATACCGTTGTTGATTGGATTCCGGGTATGAAAAAAAACATTTGTC<br>TGCGTGATCTGCCGAGCTTTATTCGTACCACCGATCCGAATGATGTGATGGTGAATTATGTGATTGGCGA<br>AATTCAGCGTACCAGCCAGAAAAGCAGCGCACTGGTTCTGAATACCTTTGACAAACTGGAAAAAGATGTT<br>CTGGATGCACTGAGCGCAATTTTTCCGAGCGTTTATGCAATTGGTCCGATTAATCTGATGCTGGATCGTG<br>TTAGTGATGAAGATCTGGATAGCATTGGTAGCAATCTGTGGAAAGAAGAAAATTGGTGTCTGCATTGGCT<br>GGATAGCCAGGATCTGGGTAGCGTTATTTATGTTAATTTTGGCAGCATTACCGTGGCAACCAAAGAGCAG<br>ATTACCGAATTTGCATGGGGTTTAGCAGATAGCAAAAAACCGTTTCTGTGGGTTATTCGTCCGGATCTGG<br>TTATTGGTGAAAGCGCAGTTCTGCCTCCAGGTTTTGGTGATGCAACCGAAGGTCGTGGTATTCTGAGCG<br>GTTGGTGTCCGCAAGAACTGGTGCTGAAACATCCGAGCATTGGTGGTTTTCTGACCCATTGTGGTTGGAA<br>TAGCATGATGGAAAGCGTTTGTAGCGGTGTGCCGGTTATTTGTTGGCCGTTTTTTGCAGAACAGCAGACC<br>AATTGTTGGTATGGTAAAAATGCCTGGGGTATTGGCATGGAAATTGATAATGAAGTGAAACGCGACGAAG<br>TTGAAGGTATGGTTCGTGAACTGATGGATGGCGAAAAAGGCAAAGAAATGAAACGTCGTGCAGCAGAAT<br>GGAAAGCAGCCGCAGAAGAGGCAGCAGCACCTGGTGGTAGCAGCCATCAGAATCTGGAAAAACTGGTT<br>GCACTGCTGCTGAGCGAACAGTAA
UGT75AB1	ATGGCACCGCCTAATTTTCTGCTGGTTACCTTTCCTGGTCAGGGTCATATTAATCCGAGCCTGCGTTTTG<br>CAGAACGTCTGATTCGTATTGGTTGTCATGTTACCTTTACCACCGCACTGAGCGCACGTCGTCGTATGAG<br>CGATAGCAAAAGCCCTCCGCCTGAAGGTCTGAGTTTTGCAACCTTTAGTGATGGTTATGATGAGGGCATT<br>AAAGAAGCAGAACTGGATCTGGATGTGTATATGAAAGAAATTACCCGTCGTGGTCCGGAAACACTGCGT<br>GAACTGATTCTGGAAAAACGTGATCGTGGCACCAATTTTACCCACATCTTTTTTACCATTCTGATGCCGTG<br>GGCAGCAGATGTTGCACAGAGCCTGGGTCTGCGTAGCACCCTGGTTTGGATTCAGCCTGCAACCGTTTT<br>TGATATCTACTACTATCACTTCAACGGCTATGACCAGCTGATTCGTTCAAGCGCAGATGCAGCAGCAGCC<br>GATAATGGTGATAGCCGTGAAATTCGTCTGCCTGGTATGCTGCCGATGACCAGCAGCTATTTTCCGAGCT<br>TTCTGGCAAGCGGTAATCAGTATCATTTTTCACTGCCGGTGATCAAACGCCATTTTGAAATTCTGAATAGC<br>GAAAAAACCGGCACCATGAAACCGAAAGTTCTGGTTAATACCTTTGAAGAACTGGAAGCAGAAGCCGTTA<br>AAGCAATTGATGAACTGAATGTTATTCCGGTGGGTCCGTTTATTCCGCTGGCATTTCTGGATGAACAGCA<br>TCCGACCGATACCAGCTTAGGTGGTGACCTGTTTCAGAAAAGCCGTGATCTGGATTATATTGACTGGCTG<br>AATAAACAGCAGGCAGCAAGCGTTATCTATATTAGCTTTGGTAGCCTGAGCCTGTTTAGCCGTCCGCAGA<br>AAGAAGAAATGGCAAAAGCACTGATTGCAATGGGTCGTCCGTTTCTGTGGGTTATTCGTAAACGTATGGG<br>TGAAGAAGAGGAAGAGGACGATAAACTGAGCTATGAAGAAGAACTGAGCAAACTGGGTATGATTGTTCC<br>GTGGTGTAGCCAGGTTGAAGTTCTGAGCAATCCGAGCGTTGGTTGTTTTGTTACCCATTGTGGTTGGAAT<br>AGCACCAGCGAAAGCCTGGTTTGTGGTGTTCCGATGGTTGGTTTTCCGCAGTGGTCAGATCAGCAGACC<br>AATGCAAAACTGGTTGAAGAGGTTTGGCGTACCGGTGTTCAGGTTGGTAAAGGCAATGGTGAAGGTGTT<br>GTTGAAGCCGGTGAAATTGAACGTTGTCTGGAAGTTGTTTTAGGTGATGGTGAAAAAGGTCGTGAACTGC<br>GTGGTAATGCAAAAAAATGGGGTGAACTGGCAAAAAAAGCAGCAAAAGATGGTGGTAGCAGCGATAATA<br>ATCTGCGTCGTTTTGTTGATGGTCTGGTTAAAGGCACCGCAAGCAGCGAATAA

Supplemental Table S10: Buffers.

Name	Ingredients	pH
UGT resuspension buffer	50 mM disodium hydrogen phosphate	7.4
	500 mM sodium chloride	
	10 mM imidazole	
	10% glycerol	
	10^{-2} *v/v* protease inhibitor Mix HP (SERVA, Heidelberg, Germany)	
	10^{-4} *v/v* Novagen® Benzonase® Nuclease (Merck)	
	25,000 U/ml lysozyme (AppliChem, Darmstadt, Germany)	
UGT assay buffer	50 mM Tris-HCl	7.5
	10% glycerol	
Gut protein extraction buffer	50 mM citric acid	6.5
	100 mM disodium hydrogen phosphate	
	5×10^{-3} *v/v* protease inhibitor Mix HP (SERVA)	
Tococa protein extraction buffer	25 mM HEPES	7.2
	3% PVPP	
	1% PVP	
	4% Amberlite XAD4	
	1 mM EDTA	
	5 mM Na_2HSO_3	
	5 mM DTT	
	5×10^{-3} *v/v* protease inhibitor cocktail III (Calbiochem)	
Glucosidase assay buffer	50 mM citric acid	6.5
	100 mM disodium hydrogen phosphate	
Infiltration buffer	10mM MES	5.7
	10mM $MgCl_2$	
	100 µM acetosyringone	
LB selection media	LB media	n.a.
	50 µg/ml kanamycin	
	10 µg/ml rifampicin	
	25 µg/ml gentamicin	

Supplemental Table S11: Thermocycler program for RT-qPCR.

Phase	Time		Temperature	
	Figure S24	Figure S13		
Initial denaturation	3 min	3 min	95 °C	
denaturation	10 s	20 s	95 °C	40×
Annealing and extension	10 s	20 s	60 °C	
Plate read				
Final denaturation	1 min	1 min	95 °C	
Melting curve	5 s	5 s	55 °C→ 95 °C	

Supplemental Table S12: List of primers used for RT-qPCR.

Name	Sequence	Amplicon length
CYP79A206_qPCR_fwd	CGACCTTCGTGTTACTTTACGC	140 bp
CYP79A206_qPCR_rev	ACGGGCTTGTTAGAGAGCATC	140 bp
CYP79A207_qPCR_fwd	ACCTTCGTCGTGCTTTACGC	150 bp
CYP79A207_qPCR_rev	TCCAACGGAACACTGGCTTG	150 bp
CYP71E76_qPCR_fwd	CGGAAACCTTCACCAACTCGG	140 bp
CYP71E76_qPCR_rev	GCACCTCCTTAGCCATCTCG	140 bp
UGT85A123_qPCR_fwd	CCCCCGTTAGTTGCGTAGTG	200 bp
UGT85A123_qPCR_rev	CGACCGTGTCCAAGTATCCGT	200 bp
UGT85A122_qPCR_fwd	ATCCCCACATTCATCCAGACGACC	140 bp
UGT85A122_qPCR_rev	CGACTTCAAGGCACGCAGAAC	140 bp
UGT75AB1_qPCR_fwd	TTCAACCCGCCACCGTCTTC	300 bp
UGT75AB1_qPCR_rev	GCCTTCACCGCTTCTGCCT	300 bp
Actin_fwd	CTCTGGTGATGGTGTCAGTCAC	140 bp
Actin_rev	TGTAACCCCTTTCGGTGAGG	140 bp

Supplemental Table S13: Accession numbers of known enzymes that were used for the construction of the phylogenetic trees.

Name	Accession number	Plant species	Name	Accession number	Plant species
**CYP79 enzymes**					
AtCYP79A2	ANM70027.1	*Arabidopsis thaliana*	MeCYP79D1	AAF27289	*Manihot esculenta*
AtCYP79B2	AEE87143.1	*Arabidopsis thaliana*	PtCYP79D6	AHF20912	*Populus trichocarpa*
AtCYP79F1	AEE29448	*Arabidopsis thaliana*	PtCYP79D7	AHF20913	*Populus trichocarpa*
AtCYP79F2	AAG24796	*Arabidopsis thaliana*	JaCYP79A68	BAP15883.1	*Prunus mume*
EcoCYP79D62	AOW44274	*Erythroxylum coca*	JaCYP79D16	BAP15884.1	*Prunus mume*
EfCYP79D60	KX344462	*Erythroxylum fischeri*	SaCYP79B1	AAD03415	*Sinabis alba*
EcCYP79A125	AYN73067	*Eucalyptus cladocalyx*	SbCYP79A1	AAA85440.1	*Sorghum bicolor*
LjCYP79D4	AAT11921.1	*Lotus japonicus*	TmCYP79E1	AAF66543	*Triglochin maritima*
MeCYP79D2	AAV97888.1	*Manihot esculenta*	TmCYP79E2	AAF66544	*Triglochin maritima*
**CYP71 enzymes**					
EcCYP706C55	AYN73068	*Eucalyptus cladocalyx*	PtCYP71B40v3	AIU56748	*Populus trichocarpa*
FsCYP71AT96	BAU59406	*Fallopia sachalinensis*	PtCYP71B41v2	AIU56747	*Populus trichocarpa*
HvCYP71C113	AK250744	*Hordeum vulgare L.*	PmCYP71AN24	BAP15888.1	*Prunus mume*
HvCYP71L1	AK248375	*Hordeum vulgare L.*	SbCYP71E1	AAC39318	*Sorghum bicolor*
MeCYP71E7	AAP57704.1	*Manihot esculenta*			
**UGT enzymes**					
AtUGT76E1	AED97208.1	*Arabidopsis thaliana*	AtUGT75B1	AEE27854.1	*Arabidopsis thaliana*
AtUGT76C1	AAP21281.1	*Arabidopsis thaliana*	AtUGT75B2	AEE27849.1	*Arabidopsis thaliana*
CrUGT76A2	BAO01108.1	*Catharanthus roseus*	AtUGT75C1	AAM47973.1	*Arabidopsis thaliana*
SlUGT76E1	NP001348276.1	*Solanum lycopersicum*	PfA5GT	Q9ZR27	*Perilla frutescens*
SrUGT76G1	AAR06912.1	*Stevia rebaudiana*	RcUGT75L20	AWU66062.1	*Rubus chingii*
AtUGT85A2	BAA34687	*Arabidopsis thaliana*	LuUGT85Q1	ADV36300	*Linum unsitatissimum*
AtUGT85A5	AAF87255	*Arabidopsis thaliana*	LjUGT85K3	CM0241.610 (gene)	*Lotus japonicus*
AtUGT85A7	AAF87257	*Arabidopsis thaliana*	MeUGT85K4	AEO45781.1	*Manihot esculenta*
AtUGT85A1	AAF18537	*Arabidopsis thaliana*	MeUGT85K5	AEO45782.1	*Manihot esculenta*
CrUGT85A23	BAK55749.1	*Catharanthus roseus*	MtUGT85H2	ABI94024.1	*Medicago truncatula*
EcUGT85A59	AYN73070.1	*Eucalyptus cladocalyx*	PlUGT85K31	VVC51334.1	*Phaseolus lunatus*
GjUGT85A24	BAK55737.1	*Gardenia jasminoides*	PdUGT85A19	ABV68925.1	*Prunus dulcis*
GjUGT94E5	BAK55744.1	*Gardenia jasminoides*	PmUGT85A47	BBE52863.1	*Prunus mume*
HvUGT85F22	AK252107	*Hordeum vulgare L.*	SbUGT85B1	AAF17077.1	*Sorghum bicolor*
HvUGT85F23	AK250769	*Hordeum vulgare L.*	SrUGT85C2	AAR06916.1	*Stevia rebaudiana*

Sequence data for all characterized enzymes can be found under the respective Genbank identifier.

Literature:

Bak S, Kahn RA, Nielsen HL, Moller BL, Halkier BA (1998) Cloning of three A-type cytochromes P450, CYP71E1, CYP98, and CYP99 from *Sorghum bicolor* (L.) Moench by a PCR approach and identification by expression in *Escherichia coli* of CYP71E1 as a multifunctional cytochrome P450 in the biosynthesis of the cyanogenic glucoside dhurrin. Plant Mol Biol **36:** 393-405

Durst F, Nelson DR (1995) Diversity and Evolution of Plant P450 and P450-Reductases. Drug Metabolism and Drug Interactions **12:** 189-206

Gachon CMM, Langlois-Meurinne M, Saindrenan P (2005) Plant secondary metabolism glycosyltransferases: the emerging functional analysis. Trends in Plant Science **10:** 542-549

Gotz S, Garcia-Gomez JM, Terol J, Williams TD, Nagaraj SH, Nueda MJ, Robles M, Talon M, Dopazo J, Conesa A (2008) High-throughput functional annotation and data mining with the Blast2GO suite. Nucleic Acids Res **36:** 3420-3435

Karabatsos GJ, Hsi N (1967) Structural studies by nuclear magnetic resonance—XI: Conformations and configurations of oxime o-methyl ethers. Tetrahedron **23:** 1079-1095

Luck K, Jirschitzka J, Irmisch S, Huber M, Gershenzon J, Kollner TG (2016) CYP79D enzymes contribute to jasmonic acid-induced formation of aldoximes and other nitrogenous volatiles in two *Erythroxylum* species. BMC Plant Biol **16:** 215

Pfaffl MW (2001) A new mathematical model for relative quantification in real-time RT-PCR. Nucleic Acids Res **29:** e45

Simao FA, Waterhouse RM, Ioannidis P, Kriventseva EV, Zdobnov EM (2015) BUSCO: assessing genome assembly and annotation completeness with single-copy orthologs. Bioinformatics **31:** 3210-3212
